# N-doped carbon quantum dots for the selective detection of OCl^−^ ions, bioimaging, and the production of Fe_3_O_4_ nanoparticles utilized in the synthesis of substituted imidazole†

**DOI:** 10.1039/d4ra06474g

**Published:** 2024-11-06

**Authors:** Namrata Priyadarshini Hota, Sathiyanarayanan Kulathu Iyer

**Affiliations:** a Department of Chemistry, School of Advanced Sciences, Vellore Institute of Technology Vellore-632 014 India sathiyanarayanank@vit.ac.in

## Abstract

Nitrogen-doped quantum dots (NCQD) were synthesized by solvothermal means using *o*-phenylenediamine and l-tartaric acid. The resultant NCQD produced a high quantum yield (40.3%) and a vivid green fluorescence. They were about 6 nm in size. The NCQD is useful in HeLa cell bioimaging investigations and is used for the fluorescence detection of OCl^−^ ions. The quantum dots' Limit of Detection (LoD) was discovered to be 40 nM. Additionally, cytotoxicity testing was conducted, and we found out that HeLa cells safely endured up to 6.5 mg ml^−1^ of NCQD. Furthermore, NCQDs were employed to synthesize Fe_3_O_4_ nanoparticles, with the quantum dots acting as a reducing and stabilizing agent. The nanoparticles exhibited remarkable catalytic activity towards organic processes due to their size of 11 nm and surface area of 67.360 m^2^ g^−1^. Excellent yields of tri-substituted imidazole derivatives were produced using Fe_3_O_4_ nanoparticles as nanocatalysts in a solvent-free method.

## Abstract 2

Nitrogen-doped quantum dots (NCQD) were synthesized by solvothermal means using *o*-phenylenediamine and l-tartaric acid.

## Graphical abstract

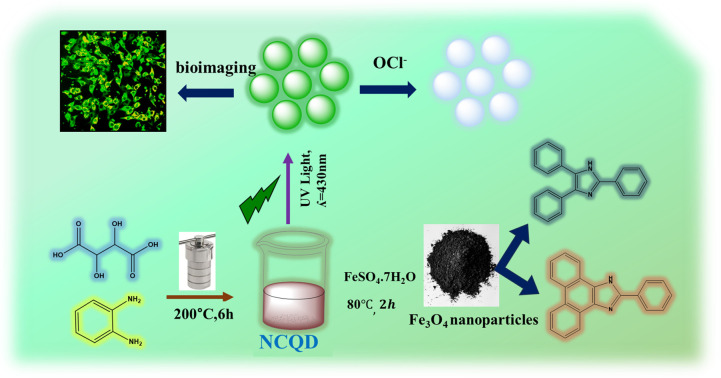

## 1 Introduction

Graphite, fullerenes, carbon nanotubes, and nanofibers are examples of carbon nanomaterials with excellent applications in drug delivery, biosensing,^1,2^ and medicine.^3^ According to a recent study, fluorescent carbon dots, a novel carbon material, have drawn increased interest because of their water solubility, high quantum yield, photoluminescence, and biocompatibility. They are a substitute for fluorescent organic molecules, electroluminescence metal–organic frameworks,^4,5^*etc.*, in several applications, including biocatalysis, bioimaging, and sensing.^6^ In 2004, Xu *et al.* accidently discovered carbon dots during the purification of single-walled carbon nanotubes. However, Sun *et al.* introduced the term “Carbon quantum dots” (CQD) in 2006 after successfully demonstrating the synthesis of fluorescent emissive carbon dots with surface passivation.^7,8^ These CQDs are zero-dimensional, quasispherical nanocrystals that are less than 10 nm (ref. 9) in size. CQDs can be produced in several ways: top-down techniques such as arc discharge,^10^ laser ablation,^11^ electrochemical exfoliation,^12^ plasma treatment,^13^ and chemical oxidation,^14^ and bottom-up techniques such as hydrothermal,^15^ solvothermal,^16^ pyrolysis,^17^ and microwave irradiation.^18^ Bottom-up synthesis is categorized as a green synthetic technique because of its low cost, ease of synthesis, and environment friendliness.^19^ CQDs are frequently used in the field of sensors because they can sense a wide range of analytes *via* quenching techniques, including Ni^2+^, Pd^2+^, Fe^3+^, Fe^2+^, Mg^2+^, K^+^, Cu^2+^, CN^−^, OCl^−^, NO_2_^−^, and many others. The different functional groups that are present on the surface of the quantum dots contribute to the CQDs' ability to detect different analytes.^10^

In aerobic organisms, Reactive Oxygen Species (ROS) can produce byproducts, as part of their physiological processes. One of the naturally occurring reactive oxygen species (ROS) is hypochlorous acid/hypochlorite ion (HOCl/OCl^−^), which is produced by the myeloperoxidase (MOP) enzyme catalyzing the peroxidation of chloride ions.^20^ An excess of OCl^−^ is harmful to health and can cause several illnesses, including cancer, atherosclerosis, renal disease, and reproductive disorders.^21^ Therefore, creating a sensor to identify OCl^−^ ions is crucial.

Bangda Yin *et al.* (2013) synthesized carbon dots from sweet red pepper to detect hypochlorite (OCl^−^) ions.^22^ Lu-Shuang Li *et al.* produced carbon dots from Hongcaitai in 2018 to detect both Cr^3+^ and OCl^−^ ions.^23^ Zhenni Wei *et al.* created carbon dots in 2019 using a solvothermal technique from 2,5-Diaminobenzenesulfonic acid in ethanol to detect ascorbic acid and OCl^−^ ions.^21^ Because of the functional groups that remain on their surface after heteroatom doping, a variety of heteroatom-doped quantum dots have also been used in the field of sensing with high quantum yields.^11^ To selectively detect OCl^−^ ions, Linlin Wang *et al.* produced N-doped carbon dots in 2021.^24^

Electron-donating and electron-withdrawing properties of these quantum dots are well established. It has can receive electrons from the species that has plenty of them and can also give electrons to species that lack electrons.^25^ This CQD can be employed for the synthesis of metal oxide nanoparticles in addition to sensing and bioimaging investigations since its surface is coated in a variety of functional groups that can serve as a source for metal nanoparticle synthesis and these CQDs function as stabilizing and reducing agents. There are several uses for these metal nanoparticles in spectroscopy, electronic devices, biology, and particularly in catalytic performances.^25,26^

In addition to producing high yields in shorter reaction times, with reduced energy usage, metal nanoparticles are difficult to remove from the reaction mixture due to their small size. Several methods have been proposed to address these issues specifically.^27^ It would be considerably simpler to separate the catalyst using an external magnetic field if the catalyst is magnetic. Recent research has revealed that iron oxide nanoparticles are supermagnetic and readily separated by an external magnetic field.^28^ Metal oxide nanoparticles has various applications in biomedical applications which includes *in vitro* and *in vivo* drug delivery^29^ as well as cancer cell detection and treatement.^30^

Multi-substituted imidazoles are one of the most important groups of pharmaceutical chemicals because they can be used for treating a wide range of disorders in clinical studies.^31^ Since these imidazole scaffolds contain carbon and nitrogen, they can bind with a variety of proteins and enzymes, increasing therapeutic efficiency.^32^ Imidazoles can be synthesized using a variety of reagents, including silica sulphuric acid (SSA),^33^ boric acid,^34^ phosphomolybdic acid,^35^ H_2_SO_4_,^36^ H_3_PO_4_,^37^ oxalic acid,^38^ and *p*-toluene sulphonic acid.^39^

In this work, we present, the creation of nitrogen-doped carbon quantum dots (NCQD) utilizing l-tartaric acid and *o*-phenylenediamine. This NCQD is used as a fluorescent probe for the selective detection of the OCl^−^ ion, bioimaging studies of HeLa cells, and also acts as a reducing and stabilizing agent in the synthesis of iron oxide nanoparticles (Fe_3_O_4_ nanoparticles). These Fe_3_O_4_ nanoparticles derived from NCQDs were used as magnetically separable catalysts in the synthesis of substituted imidazole scaffold.

## 2 Experimental

### 2.1 Material

All necessary chemicals and solvents were purchased from chemical suppliers such as Avra, Sigma Aldrich, and TCI Chemicals. Using a Bruker Ascent 400 Hz spectrometer, the chemical structure of the produced molecule was examined using ^1^H (100 MHz) spectrum. Tetramethylsilane, or TMS, was used as the internal standard reference, and DMSO as the solvent when measuring the chemical shift. To record FTIR data, a JASCO 4100 spectrometer was utilized. The UV-visible absorption studies were measured on Hitachi (model 2910) instrument and the FL emission studies were recorded on the Hitachi (model F-7000) instrument. Bruker, D8-Advance P-XRD was used for the powder XRD analysis of Fe_3_O_4_ nanoparticles. FEI – TECNAI Model G2-20 TWIN was used for the HRTEM analysis. Quantachrome USA Model AutosorbiQ was used for the surface area analysis.

### 2.2 Preparation of NCQD

**Fig. 1:**
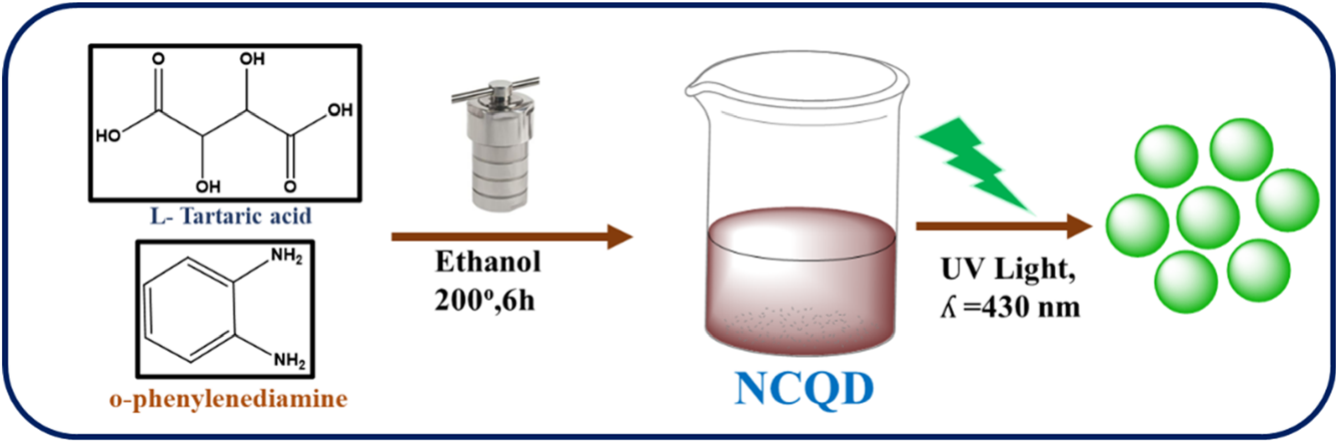
Preparation of NCQD.

l-Tartaric acid (TA) and *o*-phenylenediamine (*o*-PD) were used to synthesize the NCQD. To prepare NCQD, 1 mmol of TA and 2 mmol of *o*-PD were combined with 40 ml of ethanol and agitated for 10 minutes. The mixture was then moved to an autoclave lined with Teflon and heated to 200 °C for 6 hours. Following the completion of the reaction, the mixture yielded a yellowish-green fluorescence coloured solution when it naturally cooled to room temperature, as shown in Fig. 1. After that, Whatman filter paper was used to filter it and was then centrifuged at 4000 rpm for 10 minutes. After that, it was kept in a glass vial so that it can be used later in the preparation of Fe_3_O_4_ nanoparticles, detection of OCl^−^ ion, and bioimaging studies.

### 2.3 Fluorescence detection of OCl^−^

Double-distilled water was used to prepare 1 mmol of stock solutions for each of the anions, which were subsequently diluted to a 100 μM concentration. After keeping the pH at 7, 2 ml of 50 times diluted NCQD were placed in a cuvette, and the emission wavelength was recorded. The fluorescence intensity was then evaluated after adding 100 μl of each anion, one at a time, to the 2 ml NCQD. We chose 430 nm as the excitation wavelength for the fluorescence measurement.

### 2.4 Protocols for MTT assay

The cervical cancer cell line (HeLa) was obtained from the National Centre for Cell Science (NCCS), Pune, and cultured in Dulbecco's Modified Eagles Minimum (DMEM) with 10% fetal bovine serum. The cells were kept at 37 °C, 5% CO_2_, and 95% air. Each received 15 μl of MTT (3-[4,5-dimethylthiazol-2-yl] 2,5-diphenyltetrazolium bromide) (5 mg ml^−1^) in phosphate-buffered saline (PBS) after 48 hours of incubation and the wells were then incubated at 370 °C for 4 hours. After turning off the MTT medium, the formazan crystals were dissolved in 100 μl of DMSO, and the absorbance at 570 nm was measured with a microplate reader.

### 2.5 Procedure for bioimaging studies

HeLa cells were seeded in a 24-well tissue culture plate at a density of 5 × 10^5^ cells per ml. The cells were then treated with 50 and 100 μl of NCQD in DMEM medium without serum. The plate was incubated for 24 hours at 37 °C with 5% CO_2_. Following the incubation period, 50 μl each of ethidium bromide and acridine orange (1 mg ml^−1^) were added to the wells and gently mixed. Ultimately, the plate was centrifuged for two minutes at 800 rpm, assessed right away within an hour, and at least 100 cells were viewed under a fluorescent microscope with a fluorescent filter.

### 2.6 Synthesis of Fe_3_O_4_ nanoparticles

**Fig. 2:**
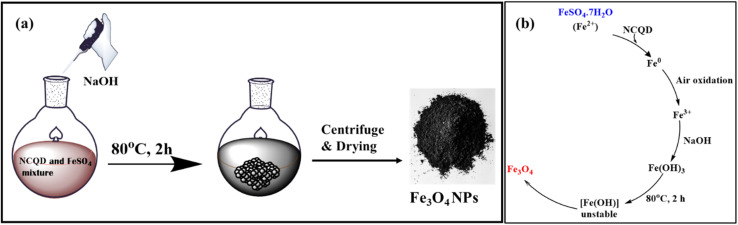
(a) Preparation of Fe_3_O_4_ nanoparticles, (b) mechanism for synthesis of nanoparticles.

Fe_3_O_4_ nanoparticles were synthesized using NCQD, which served as both stabilizing and reducing agent. A 0.1 M FeSO_4_·7H_2_O (2.79 g) solution was prepared in 100 ml of double-distilled water while stirring continuously. After adding 20 milliliters of NCQD to the mixture, it was placed in an oil bath at 80 °C. Then, 0.2 M of NaOH was added dropwise to keep the pH between 11 and 12, and the mixture was stirred for two hours, as given in Fig. 2(a). The mixture was found to be brown at first, but after NaOH was added, it turned black, signifying the creation of Fe_3_O_4_ nanoparticles. The mechanism of synthesis of Fe_3_O_4_ nanoparticles^40^ is given in Fig. 2(b). The mixture was centrifuged multiple times using acetone, ethanol, and water after it reached room temperature. After being dried in an oven and verified by several characterization methods, the Fe_3_O_4_ nanoparticles were utilized as a catalyst to create substituted imidazole scaffolds.

## 3 Results and discussion

### 3.1 Characterization of NCQD and Fe_3_O_4_ nanoparticles

#### 3.1.1 Characterization and optical behavior of NCQD

**Fig. 3:**
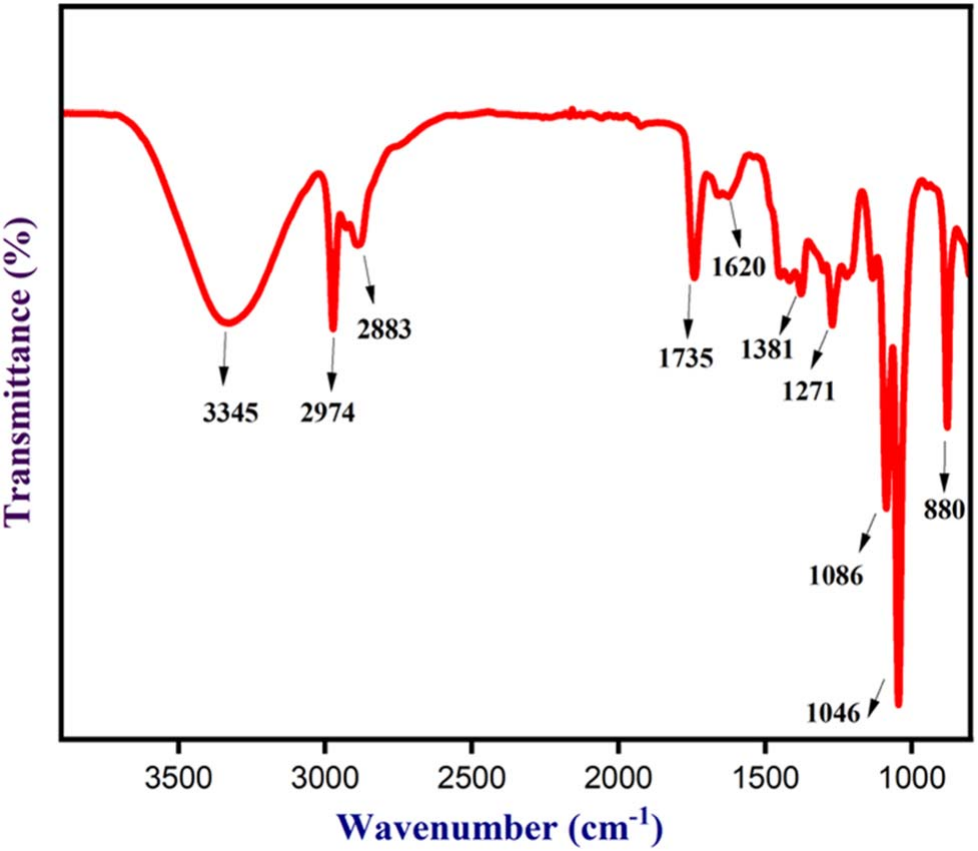
FT-IR spectrum of NCQD.

The figure (Fig. 3) depicts the FTIR spectrum of NCQD, which demonstrates the presence of functional groups. The noticeable peaks at 3345, 2883, 1735, 1620, 1381, 1271, 1086, 1046, and 880 cm^−1^ are attributable to the presence of stretching/bending vibration of –OH/–NH groups,^41^ aromatic/aliphatic –C–H of alkane, –C–H stretching of aldehyde, –C=O of aldehyde, –C=C of unsaturated ketones, –C–H bending of aldehyde, –OH of carboxylic acid, –C–O of primary alcohol, –C–O of ether, and –C=C of alkene. HRTEM analysis was used to investigate the size and morphology of the NCQD.

**Fig. 4:**
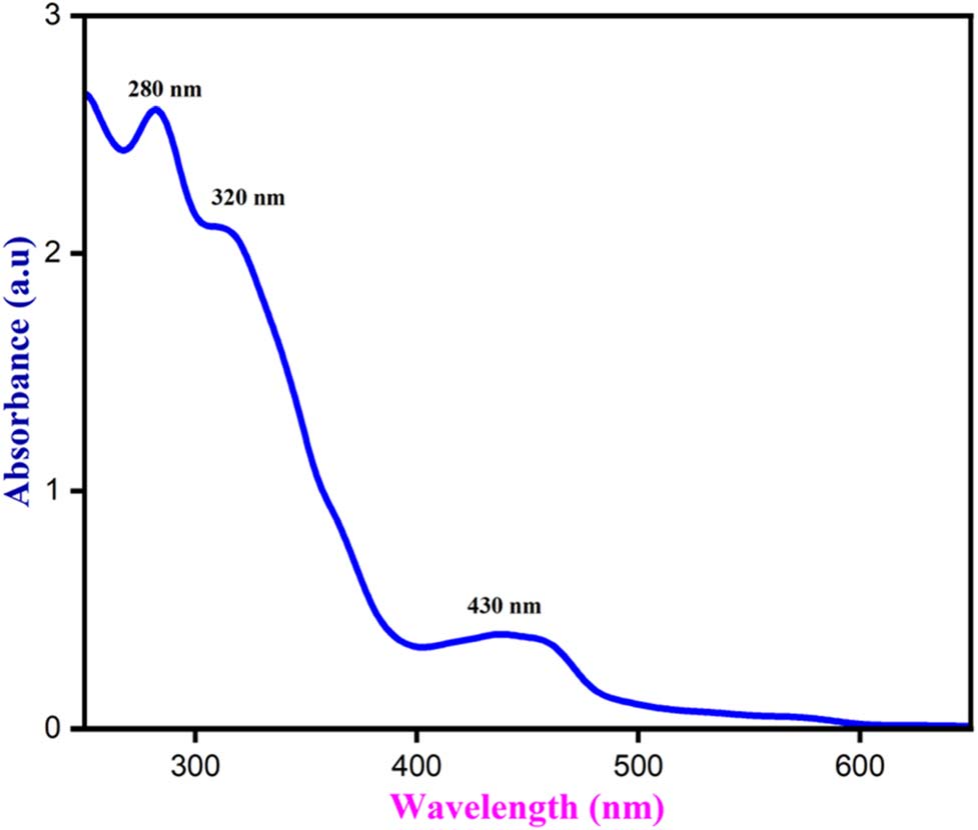
UV-visible spectrum of NCQD.

When exposed to UV light, the NCQD emitted a bright green color and displayed a bright yellow color visible to the naked eyes. Both, UV-visible and fluorescence spectra, were obtained to investigate the optical characteristics of the NCQD. As seen in Fig. 4, the absorption spectra exhibited three distinct peaks at 280 nm, 350 nm, and 430 nm. The transition, known as π–π* from C=C and C=N (resulting from nitrogen doping) is responsible for the absorption peak at 280 nm. Peaks at 370 and 430 nm are ascribed to the N=C and C=O n–π* transitions. The *o*-phenylenediamine serves as a nitrogen supply for the carbon dots' doping, which is responsible for the absorption band at 430 nm. The NCQD displayed bright green emission at 500 nm when excited at 430 nm, suggesting that it possesses a commonly observed luminous characteristic. Perhaps due to surface and NCQD size defects, this NCQD, like other carbon dots, displays excitation-dependent photoluminescence (PL) behavior (Fig. S1(a)†).

Fig. S1(b)† depicts the pH effect of the NCQD at various pH levels (2 to 12). To prepare the pH solution, 0.1 M HCl and NaOH were used and adjusted using a pH meter to the desired pH. Lower pH 2 causes a decrease in FL intensities, which continue to increase until pH 7. This could be caused by protonation taking place at a lower pH because the functional groups (–NH, –OH, *etc.*) are present on the NCQD surface.^42–44^ Basic pH exhibits a minor decrease in intensity and remains constant, but neutral pH has a high intensity compared to acidic or basic pH. These pH experiments indicate that neutral pH will be suitable for sensing and bioimaging studies.

##### 3.1.1.1 Selective and sensitive detection of OCl^−^

**Fig. 5:**
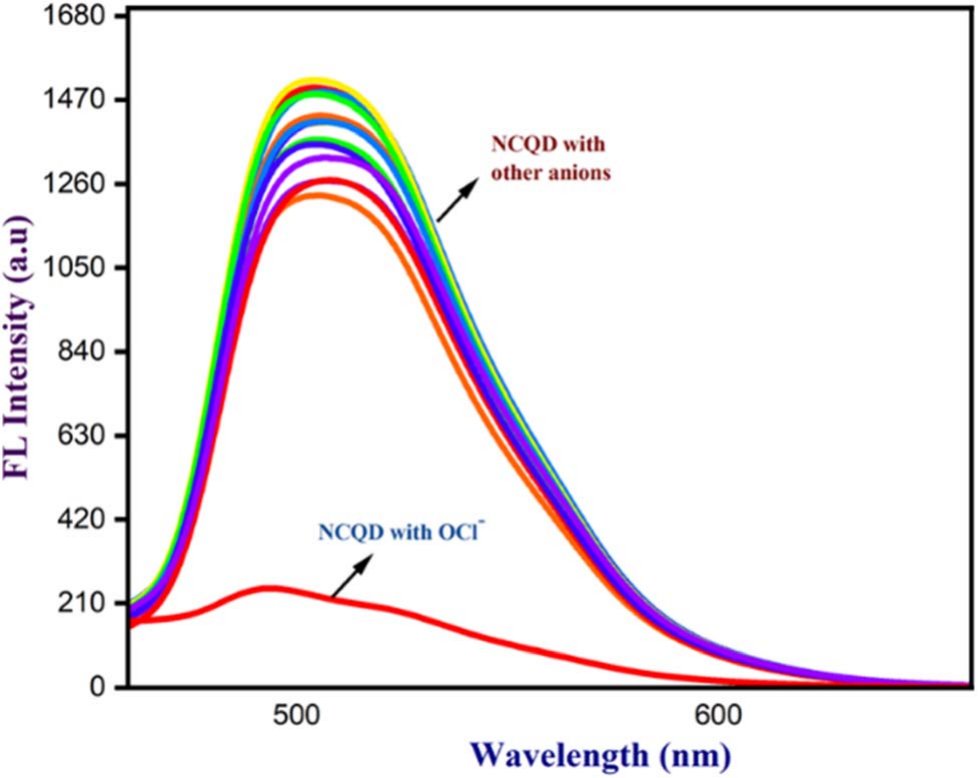
Selectivity of NCQD with various anions.

The synthesized NCQD served as a fluorescent probe for the selective detection of OCl^−^ ion. Several competitive anions, including CN^−^, CO_3_^2−^, F^−^, Cl^−^, Br^−^, HCO_3_^−^, CH_3_COO^−^, NO_2_^−^, NO_3_^−^, SO_4_^2−^, I^−^, PO_4_^−^, OH^−^, and HSO_4_^−^, were employed to evaluate the interaction with NCQD. To investigate the selectivity, 2 ml of 50 times diluted NCQD (pH = 7) was combined with 100 μm of each anion at a time. Fluorescence spectra were taken at 500 nm after exciting to 430 nm. Apart from the OCl^−^ ion, Fig. 5 shows that other anions have a negligible effect on the fluorescence spectra. The addition of OCl^−^ ion causes a considerable change, indicating that this fluorescence probe is only selective for OCl^−^ ion. The probe emitted bright green fluorescence under the UV light before adding OCl^−^ ions, and the addition of OCl^−^ ions instantly quenches the fluorescence of the solution.

**Fig. 6:**
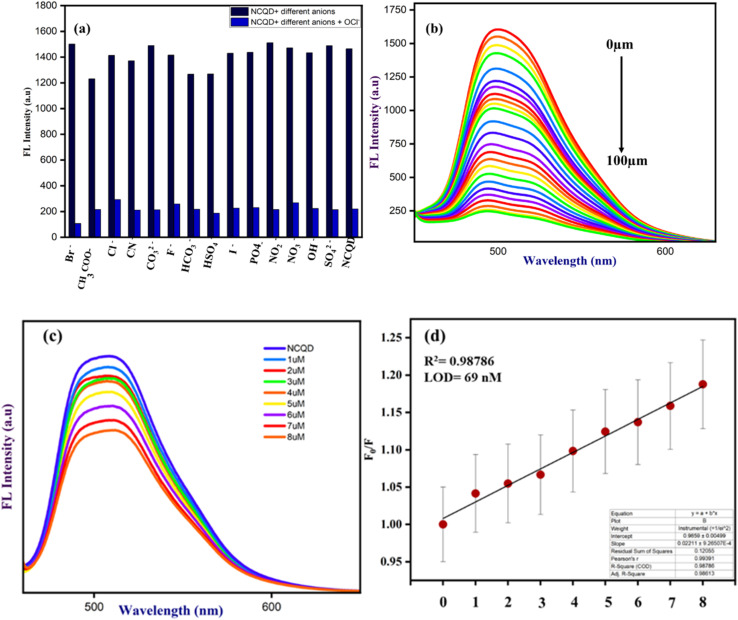
(a) The interference of OCl^−^ ion with different anions, (b) the titration of different concentrations of OCl^−^ ion with NCQD, (c) titration of NCQD, and (d) calibration plot of NCQD with varying concentrations of OCl^−^(0–8 μM).

Additionally, the most interfering anions, such as CN^−^, CO_3_^2−^, F^−^, Cl^−^, Br^−^, HCO_3_^−^, CH_3_COO^−^, NO_2_^−^, NO_3_^−^, SO_4_^2−^, I^−^, PO_4_^−^, OH^−^, and HSO_4_^−^, were added in the presence of OCl^−^ ions to assess the impact of other anions on OCl^−^ bonded with NCQD. The addition of OCl^−^ ion to NCQD resulted in a change in fluorescence intensity, which was maintained when additional anions were added to NCQD in addition to the OCl^−^ ion as shown in Fig. 6(a). This implies that the detection of OCl^−^ ion is not affected by these anions. The NCQD's fluorescence intensity rapidly decreased as OCl^−^ ion was added, as seen in Fig. 6(b). The fluorescence intensity of the NCQD decreased as the OCl^−^ ion concentration increased. The titration process continued until the NCQD solution contained 100 μm OCl^−^. Within five seconds, the NCQD's emission intensity dropped with the addition of OCl^−^ as shown in Fig. S2(a).†

The relative fluorescence response of NCQD (*F*_0_/*F*) *vs.* the concentration of OCl^−^ (in μm) plot, as shown in Fig. S2(b),† was used to determine the limit of detection (LOD) for NCQD. where *F*_0_ and *F* denote the fluorescence intensity before and after adding OCl^−^. The correlation coefficient (*R*^2^) which was 0.99627 indicated a good linear relationship, with a linear range of 0 to 8 μm. Based on the formula given below, the LOD was calculated as 40 nM.LOD = 3*σ*/SlopeThe symbol *σ* denotes standard deviation. The LOD for this one is comparable to the others. Table S1† contains a comparison table for several carbon quantum dots.

###### 3.1.1.1.1 Mechanism of OCl^−^ sensing

From the FTIR data, it is clear that functional groups like –OH, –NH, *etc.* are present on the surface of NCQD which stabilizes the carbon dots. The carbon dots are sensitive to reactive oxygen species (ROS) like OCl^−^ which is widely used as an oxidant. It can be assumed that the functional groups of the carbon dots interact with the ROs and get oxidized which causes the quenching of the emission spectra. In this way, functional groups on the surface of NCQDs are the primary cause of OCl^−^ ion sensing.^21^

###### 3.1.1.1.2 MTT assay and bioimaging studies

**Fig. 7:**
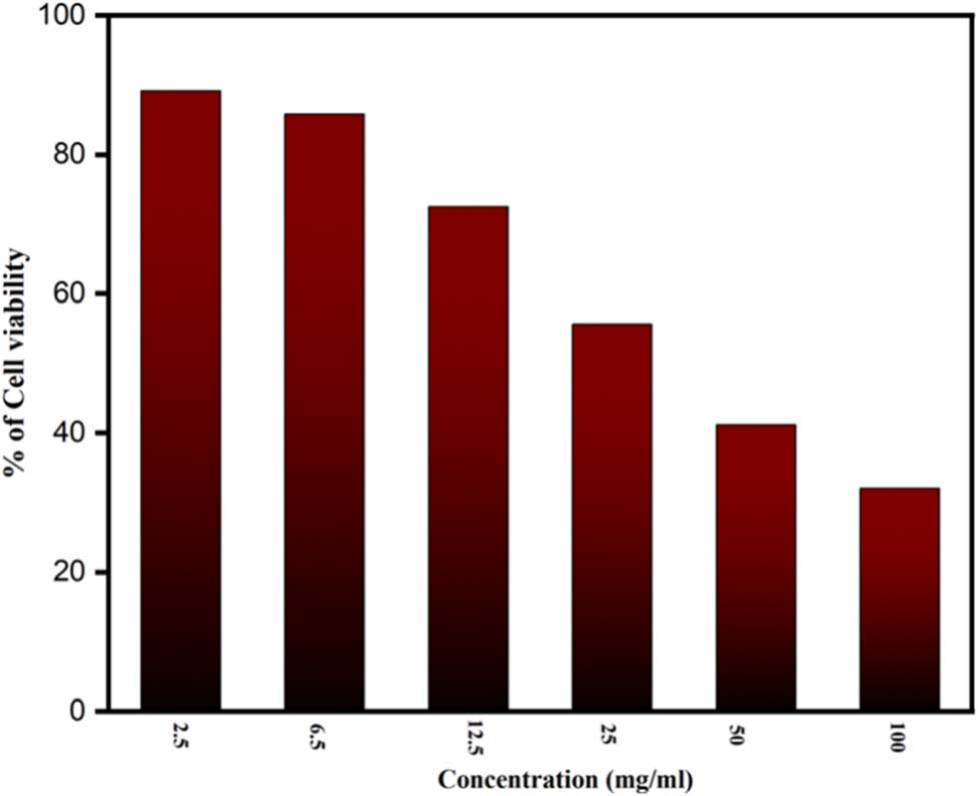
The % of cell viability of NCQD estimated from the MTT assay.

The MTT test was conducted, as indicated in Fig. 7, to investigate cytotoxicity and cell viability. It shows how different NCQD concentrations were made and examined for cell viability. Comparing the 2.5 mg ml^−1^ concentration of NCQD to the control, the survival rate was 89.11%, which is acceptable. However, the survival rate of the cells dropped slightly when the dose was increased to 6.5 mg ml^−1^. Additionally, the survival rate continued to decline until it reached 38% when the NCQD concentration rose from 12.5 mg ml^−1^ to 100 mg ml^−1^. It implies that the concentrations in the range of 2.5–6.5 mg ml^−1^ are safe for HeLa cells. This investigation indicated that the material exhibited good biocompatibility behavior against HeLa cells.

**Fig. 8:**
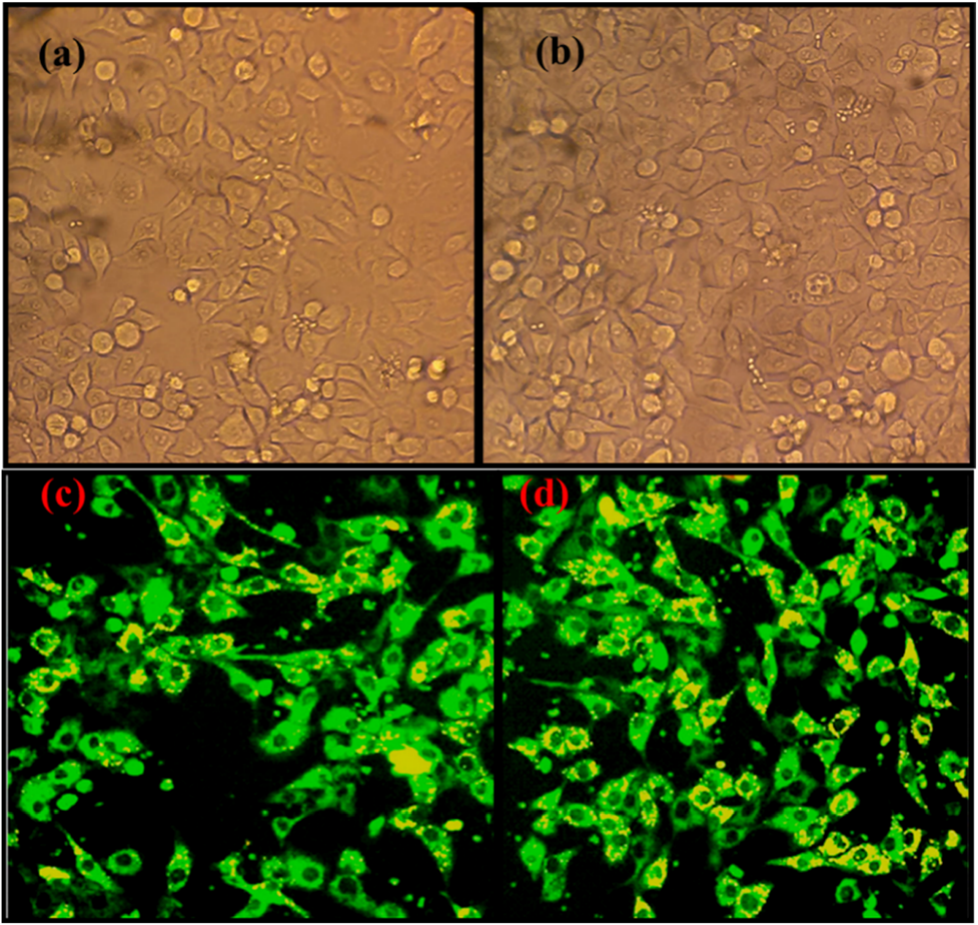
The HeLa cells (a and b) the bright field image without the NCQD, and (c and d) fluorescence image in the presence of NCQD with 50 μl and 100 μl concentration respectively.

Additionally, NCQD was used only for fluorescent bio-imaging of the HeLa cell and the resultant image is displayed in Fig. 8. The addition of various NCQD concentrations to HeLa cells demonstrates the variation in the fluorescence picture. Because of the great penetration of NCQD into the HeLa cells, the 100 μl of the CQD exhibits bright fluorescence cell pictures (Fig. 8d) in comparison to the 50 μl (Fig. 8c) of the CQD.

#### 3.1.2 Characterization and application of Fe_3_O_4_ nanoparticles

Various characterization techniques were used to investigate the synthesized Fe_3_O_4_ nanoparticles, including Fourier Transform Infrared Spectroscopy (FT-IR), Powder X-ray diffraction (p-XRD), Field Emission Scanning Electron Microscope (FESEM), Energy Disperse X-ray (EDS), High-Resolution Transmission Electron Microscope (HRTEM), Brunauer–Emmitt–Teller (BET) studies, Thermogravimetric Analysis (TGA), vibrating sample magnetometer (VSM) test and Induced Coupled Plasma-Optical emission Spectroscopy (ICP-OES).

**Fig. 9:**
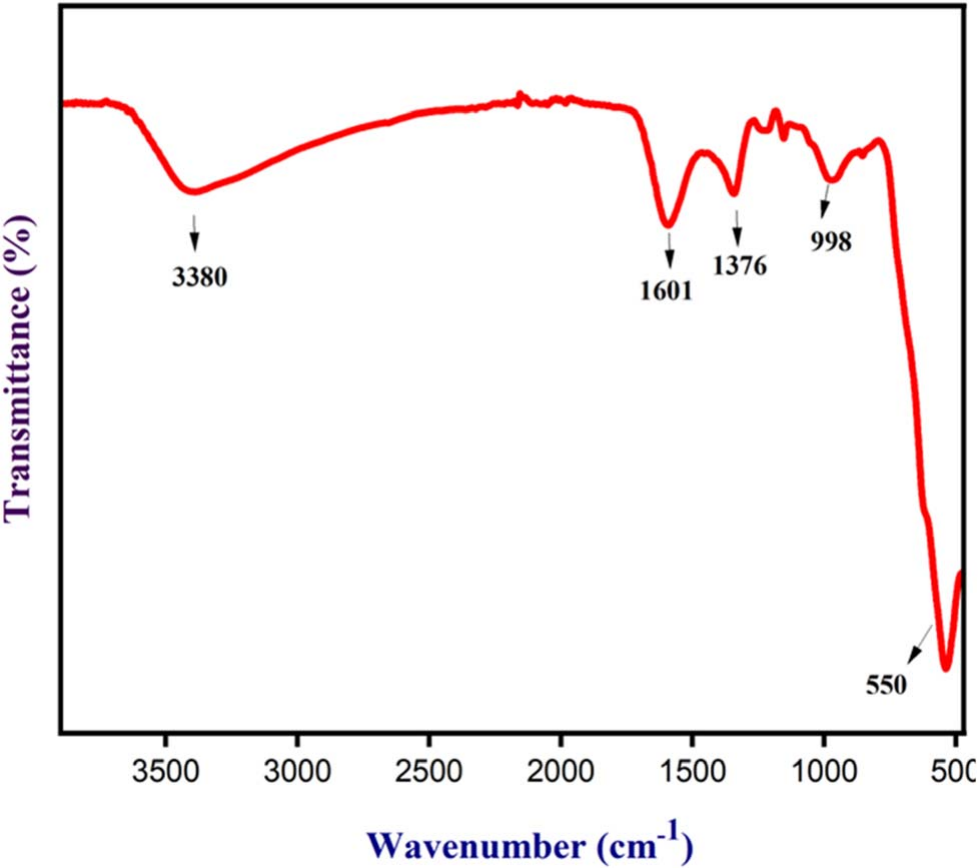
The FT-IR analysis of Fe_3_O_4_ nanoparticles.

The FT-IR analysis of Fe_3_O_4_ nanoparticles was done to determine their functional group, as shown in Fig. 9. Fe_3_O_4_ nanoparticles showed a peak at 3380 cm^−1^ that can be ascribed to the –OH/–NH groups. Further, Fe_3_O_4_ nanoparticles exhibited peaks at 1601, 1373, and 998 cm^−1^ due to C=C of unsaturated ketones, –C–H of aldehyde, and –C–O of ether, respectively, resulting from the functional group present in the NCQD absorbed by Fe_3_O_4_ nanoparticles. Another significant peak at 550 cm^−1^ was caused by the Fe–O bond, indicating the existence of Fe_3_O_4_ nanoparticles. The interaction of the metal and functional groups caused a minor shift in the spectra.

**Fig. 10:**
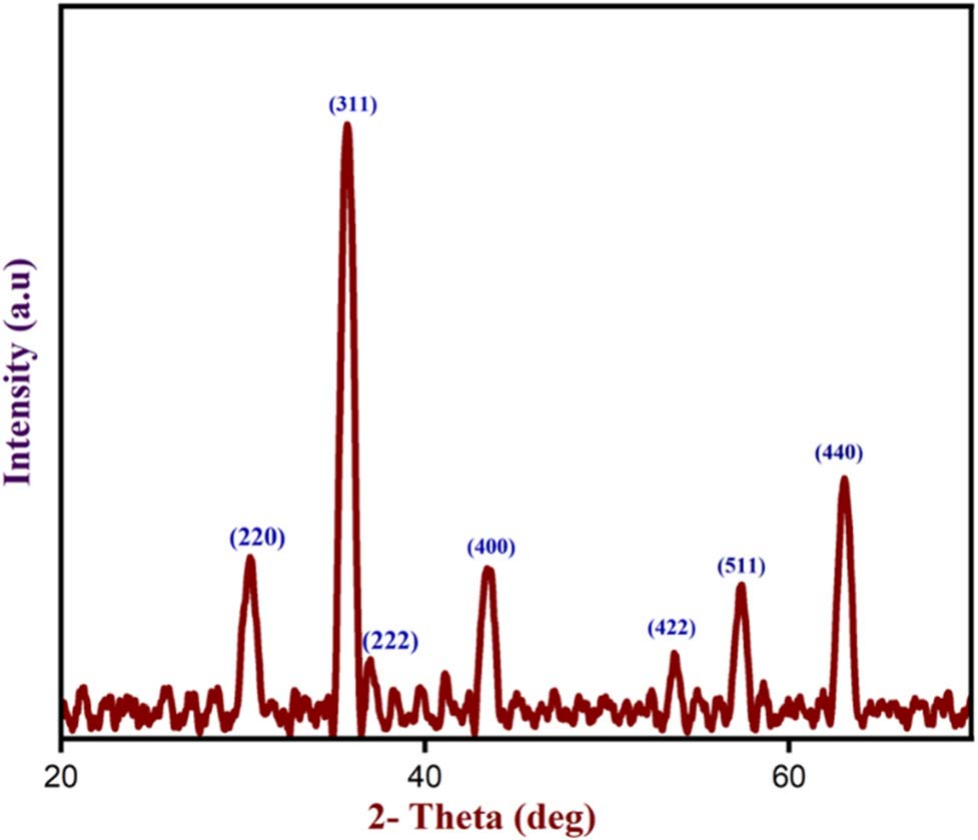
The XRD analysis of Fe_3_O_4_ nanoparticles.

Besides, p-XRD analysis was performed to determine the crystallinity and phase composition of the produced Fe_3_O_4_ nanoparticles. The diffraction peaks of Fe_3_O_4_ nanoparticles at 2*θ* values of 30.27°, 35.71°, 36.93°, 43.39°, 53.68°, 57.30°, and 63.03° correspond to the (220), (311), (222), (400), (422), (511), and (440) crystalline planes respectively as shown in Fig. 10. These planes were fully indexed to the Fe_3_O_4_ nanoparticles's structure, which corresponded with JCPDS card number 11-0614. The average size of Fe_3_O_4_ nanoparticles is 13 nm, estimated with the formula *D* = (0.94*λ*)/*β* cos *θ*.

**Fig. 11:**
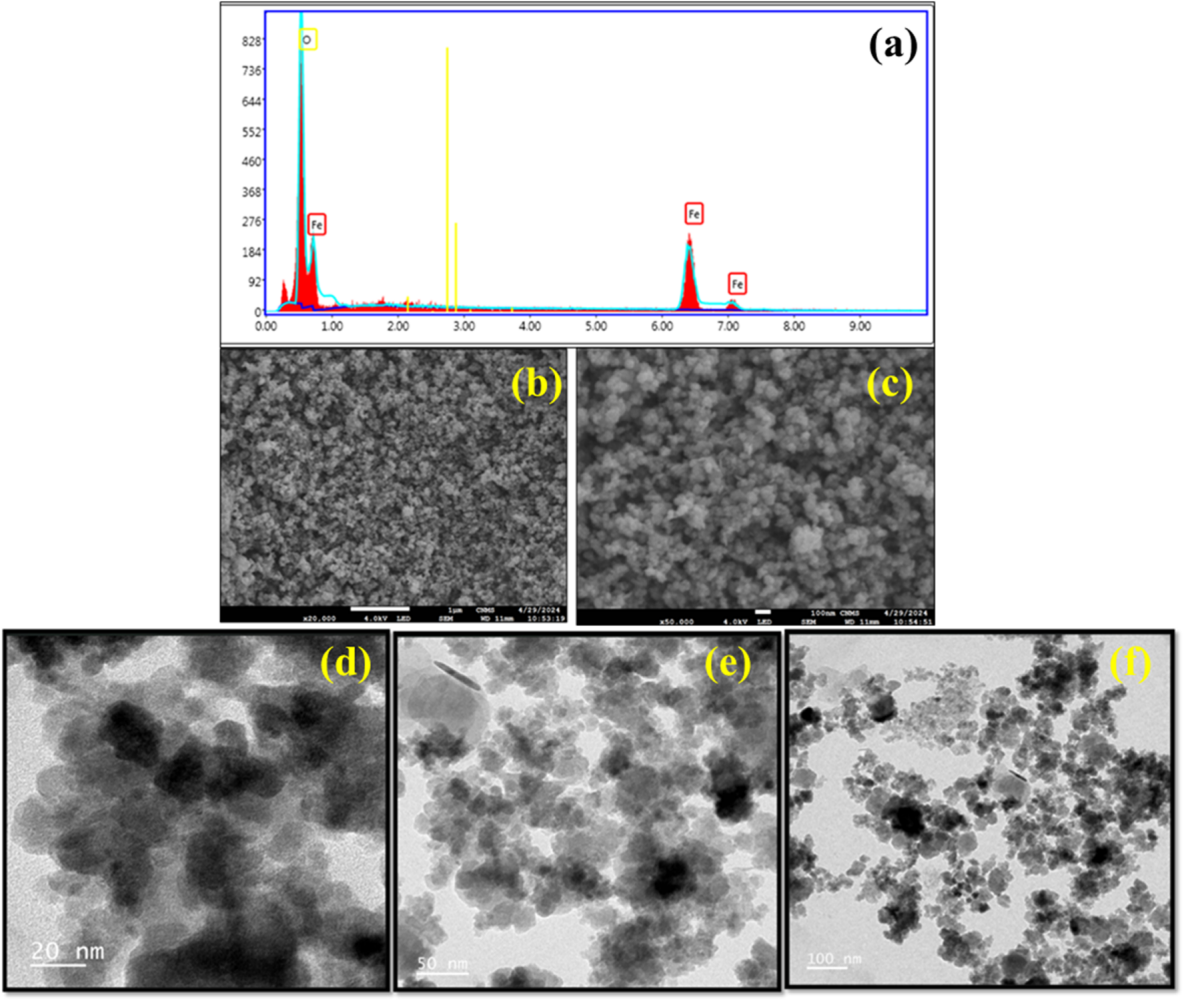
(a) The EDAX analysis, (b and c) FESEM analysis, and (d–f) HRTEM analysis of Fe_3_O_4_ nanoparticles.

FESEM analysis was performed to determine the surface morphology and shape of the produced Fe_3_O_4_ nanoparticles. The results demonstrated that the presence of functional groups adhered to the catalyst's surface inducing Fe_3_O_4_ nanoparticles had an agglomerated spherical morphology Fig. 11(b and c). Additionally, an EDAX analysis revealed the elemental composition of iron and oxygen in the Fe_3_O_4_ nanoparticles, as seen in Fig. 11(a), which shows that the catalyst contains 68% iron (Fe). After that, an ICP-OES analysis revealed that the catalyst contained 43.7% Fe.

HRTEM examination was performed to confirm the enlarged shape and size of the Fe_3_O_4_ nanoparticles. The HRTEM examination verified that the Fe_3_O_4_ nanoparticles had an agglomerated spherical morphology and varied in size from 6 to 15 nm, as depicted in Fig. 11(d–f). The average particle size was determined to be 11 nm, which is in good agreement with the XRD analysis.

**Fig. 12:**
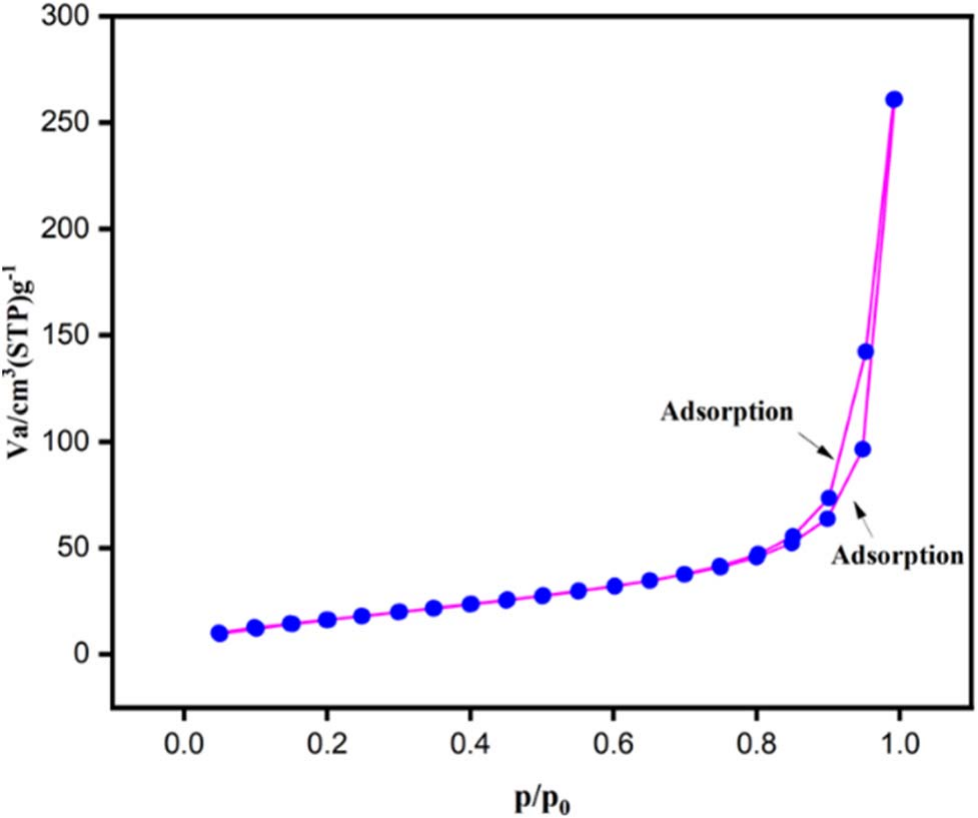
The BET analysis of Fe_3_O_4_ nanoparticles.

The surface area and porosity of Fe_3_O_4_ nanoparticles were determined using BET analysis, it was discovered that it had a pore diameter of 1.431 nm and a surface area of 67.360 m^2^ g^−1^. The Fe_3_O_4_ nanoparticles' N_2_ adsorption–desorption isotherm is displayed in Fig. 12, which shows the features of a mesoporous material with a type II isotherm and hysteresis loop. It served as an excellent nanocatalyst because of its greater surface area.

**Fig. 13:**
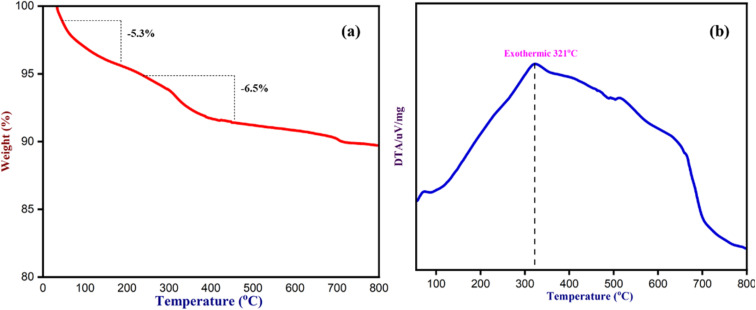
(a) The TGA and (b) The DTA of Fe_3_O_4_ nanoparticles.

To determine the thermal stability of Fe_3_O_4_ nanoparticles and the chemicals absorbed by NCQD on Fe_3_O_4_ nanoparticles, a thermogravimetric (TG) analysis was carried out (Fig. 13). The TG analysis was performed in a nitrogen atmosphere between 35 and 800 °C, heating at a rate of 10 °C per minute. The initial 5–6% weight loss that happened up to 150 °C was due to the moisture absorbed by the catalyst during the synthesis process. From 200 °C to 800 °C, there was a drastic weight loss of over 6% because the volatile organic molecules in the NCQD were removed. The whole functional groups on the surface of NCQD that were absorbed by the Fe_3_O_4_ nanoparticles were responsible for a total weight loss of around 11% at about 800 °C, suggesting that the catalyst was stable even in higher temperatures. The DTA/TG graph indicates that the maximum weight loss occurred at 321 °C, confirming the sustainability of Fe_3_O_4_ nanoparticles even at higher temperatures.

**Fig. 14:**
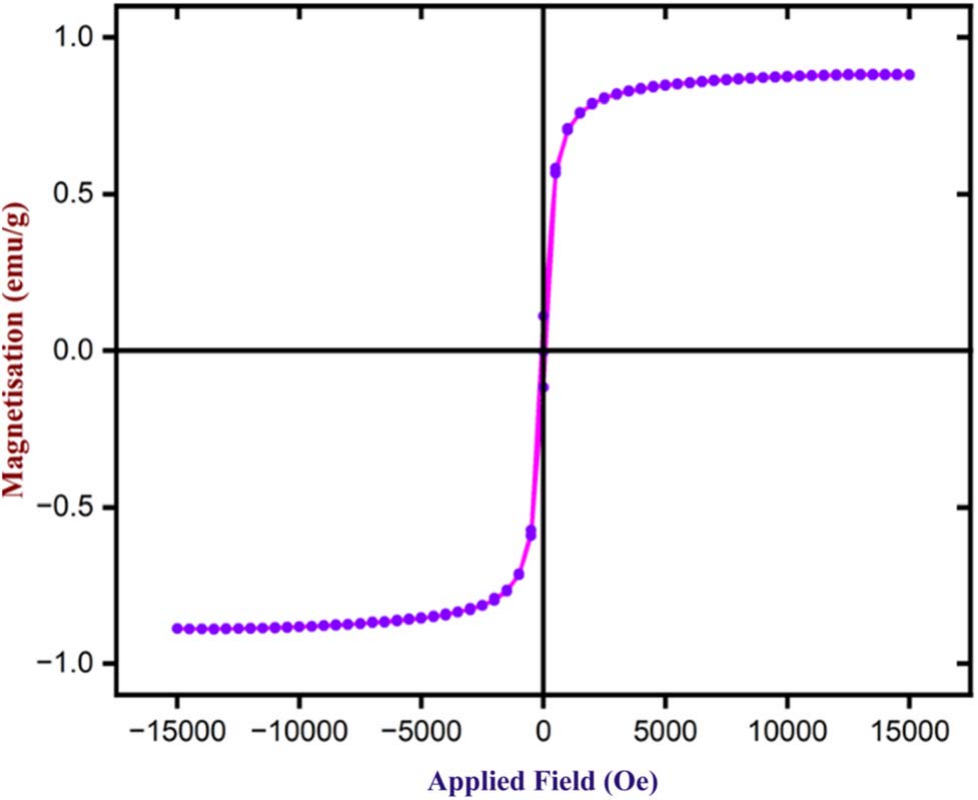
The VSM analysis of Fe_3_O_4_ nanoparticles.

To determine the magnetic characteristics of Fe_3_O_4_ nanoparticles, VSM analysis was performed using a magnetic field ranging from −1500 to +1500 Oe at ambient temperature. Fig. 14 illustrates the sample's hysteresis loops. Fe_3_O_4_ nanoparticles exhibit magnetic saturation (Ms) of 88.5 emu g^−1^. Further, the Coercivity (Hci) and Retentivity (Mr) are 83.46 Oe and 0.11 emu, indicating that they exhibit supermagnetic properties. As a result, Fe_3_O_4_ nanoparticles may be easily isolated from the reaction mixture using an external magnetic bar after the completion of the reactions.

##### 3.1.2.1 Application of Fe_3_O_4_ nanoparticles in substituted 2,4,5-triphenyl-1*H*-imidazole reaction (4)

The Fe_3_O_4_ nanoparticles' catalytic activity was ascertained after effective synthesis and characterization of substituted 2,4,5-triphenyl-1*H*-imidazole. To optimize the synthesis, 4-cyanobenzaldehyde, benzil, and NH_4_OAc as a nitrogen source were combined as a model reaction. An external magnetic bar was utilized to remove the catalyst when the reactions were finished, and thin-layer chromatography (TLC) was employed to monitor the reaction process. After the reaction mixture was extracted with ethyl acetate and brine solution, the ethyl acetate layer was gathered and dried with sodium sulfate. Next, H^1^ NMR was used to confirm the products after they had been purified using column chromatography. Fe_3_O_4_ nanoparticles recycled for the 4th time after this reaction is shown in Fig. S4.†

The synthesis of substituted 2,4,5-triphenyl-1*H*-imidazole was optimized using a range of solvents, including water, ethanol, acetonitrile, THF, DMF, toluene, and so on, along with Fe_3_O_4_ catalyst, as Table S3† illustrates. However, the yields obtained were not adequate. Additionally, entries 10 and 11 in Table S3†, show that the reactions were conducted without catalysts, which resulted in a very low yield. With a catalyst, ethanol as a solvent (Table S3†, entry 9) produced an excellent yield; however, the yield was relatively low in the absence of the catalyst. Then, solvent-free reaction conditions were selected after experimenting with various reaction conditions. The yield in the solvent-free condition was the best compared to ethanol solvent. The reaction was evaluated in solvent-free conditions both with and without a catalyst, and the results showed that even after holding the reaction longer, the yield was lower in the absence of a catalyst. Next, the catalyst's loading was examined, indicating that a decrease in catalyst loading led to a lower yield. The plausible mechanism for this reaction is shown below.
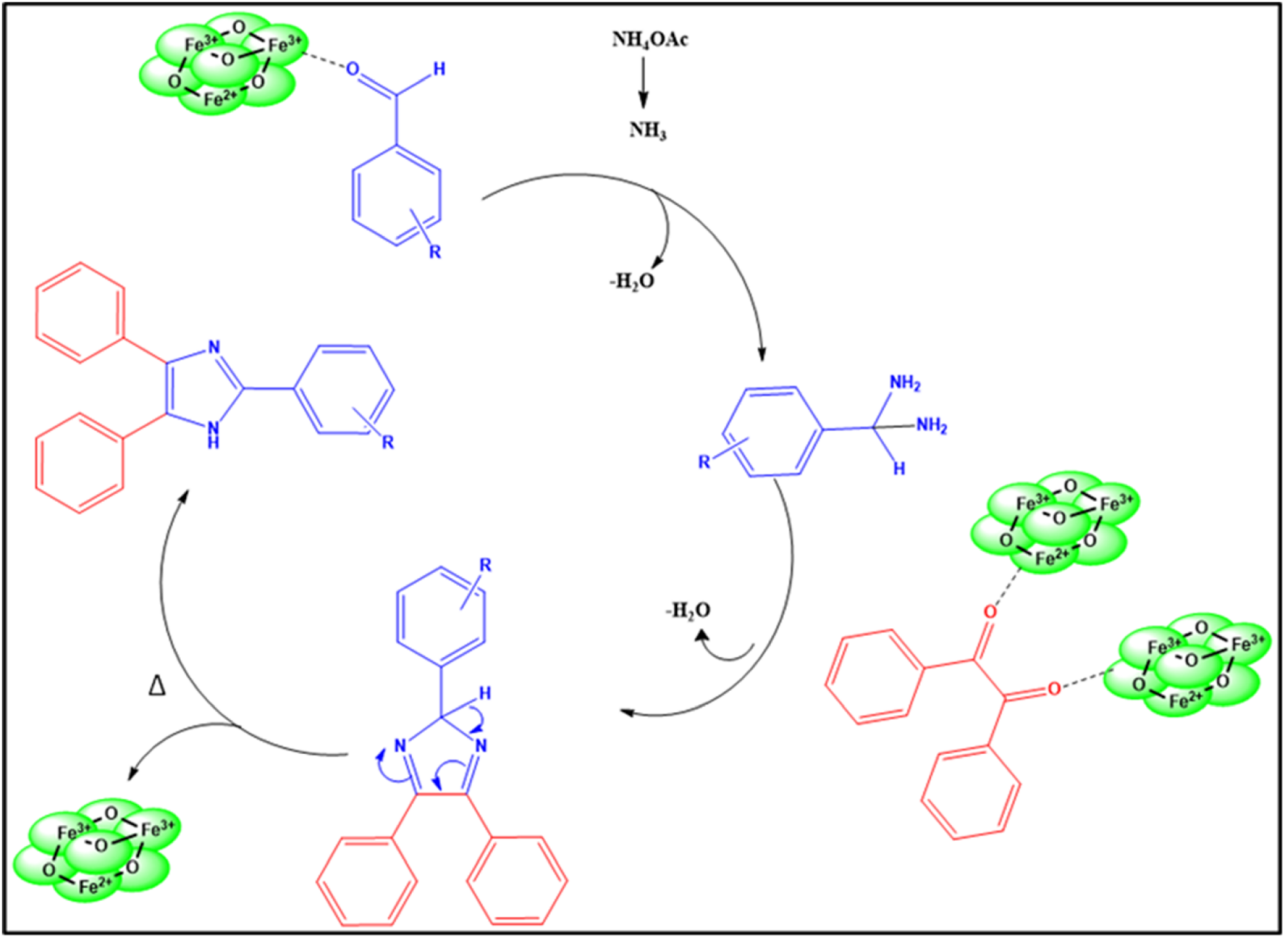


**Scheme 1:**
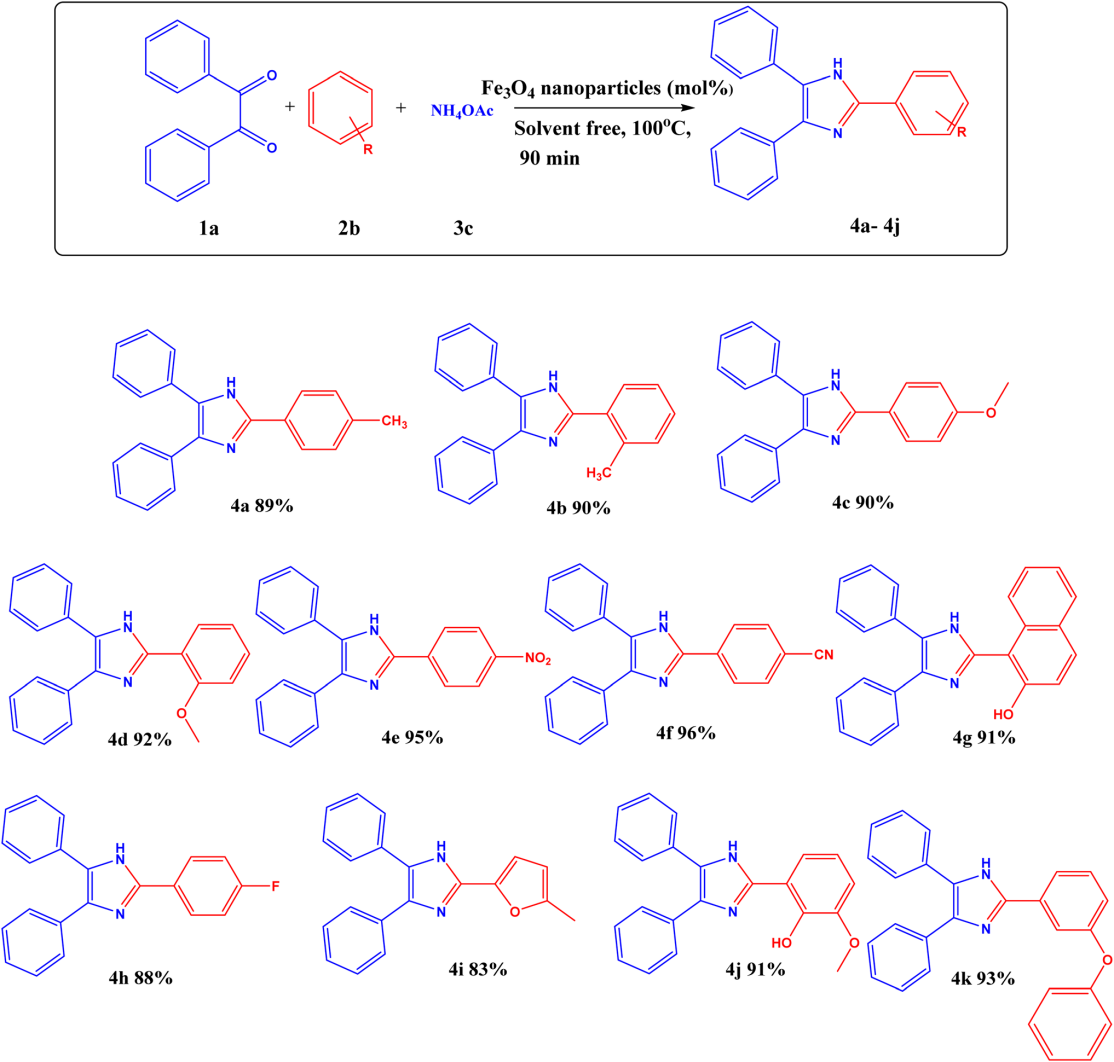
Reaction conditions: benzil (1 eq.), aldehyde (1.2 equivalents), NH_4_OAc (2.5 equivalents), Fe_3_O_4_ nanoparticles (3.3 mol% respect to the benzil), solvent-free at 100 °C for 90 min, yield (%) isolated yield after separation by column chromatography.

Additionally, the ideal conditions for synthesizing substituted 2,4,5-triphenyl-1*H*-imidazole with Fe_3_O_4_ nanoparticles are as follows: benzil (1 equivalent), substituted aldehyde (1.2 equivalents), NH_4_OAc (2.5 equivalents), and Fe_3_O_4_ nanoparticles (3.3 mol%) at 100 °C for 90 minutes in solvent-free condition. This is listed in (Table S3†, entry 15). A variety of substituted aldehydes, including 2-tolualdehyde (4b), 4-tolualdehyde (4a), 2-anisaldehyde (4d), 4-anisaldehyde (4c), 4-fluorobenzaldehyde (4h), 4-cyanobenzaldehyde (4f), 4-nitrobenzaldehyde (4e), 2-vannilin (4j), 5-methylfuraldehyde (4i), 3-benzyloxybenzaldehyde (4k) and 2-hydroxynapthaldehyde (4g) were use to synthesize variety of substituted imidazole in good yields (Scheme 1).

##### 3.1.2.2 Application of Fe_3_O_4_ nanoparticles in substituted 2-phenyl-1*H*-phenanthro[9,10-*d*] imidazole reaction (8)

After getting an excellent yield from the substituted 2,4,5-triphenyl-1*H*-imidazole reactions, Fe_3_O_4_ nanoparticles were explored for the production of substituted 2-phenyl-1*H*-phenanthro[9,10-*d*] imidazole reaction. A model reaction was employed to maximize the synthesis with 3-anisaldehyde as the substituted aldehyde, 9,10-phenanthrenequinone, and NH_4_OAc as a nitrogen supply. Once the reactions were finished, the catalyst was removed with the aid of an external magnetic bar, and TLC was used to track the progress of the reactions. Next, ethyl acetate and brine solution were used to extract the reaction mixture, and sodium sulfate was used to dry the ethyl acetate layer. Then, column chromatography was employed to purify the products, and ^1^H NMR was used to confirm them. The efficiency of Fe_3_O_4_ nanoparticles recycled for the 4th time after this reaction is shown in Fig. S4.†

vTo optimize the reaction conditions, a variety of solvents were utilized, including acetone, H_2_O, EtOH, EtOH:H_2_O, THF, acetonitrile, DMF, and toluene in the presence of Fe_3_O_4_ nanoparticles, as in earlier imidazole (4) procedures. None of them produced the expected yield due to solvent effects. The reaction was carried out using EtOH solvent with and without a catalyst (Table S4†, entries 9 and 10). The reaction with the catalyst in EtOH solvent produced a higher yield than without the catalyst. Then the reactions were carried out in solvent-free conditions, with and without catalyst, and with different catalyst concentrations. These circumstances indicated that, in solvent-free conditions, it produced higher yields than in the EtOH medium. The reaction without a catalyst under solvent-free conditions yields less than those with a catalyst (Table S4†, entries 11–15). The catalyst's loading was then investigated, which indicated that a decrease in catalyst loading reduced the yield. The plausible mechanism for this reaction is shown in Fig. S3.†

**Scheme 2:**
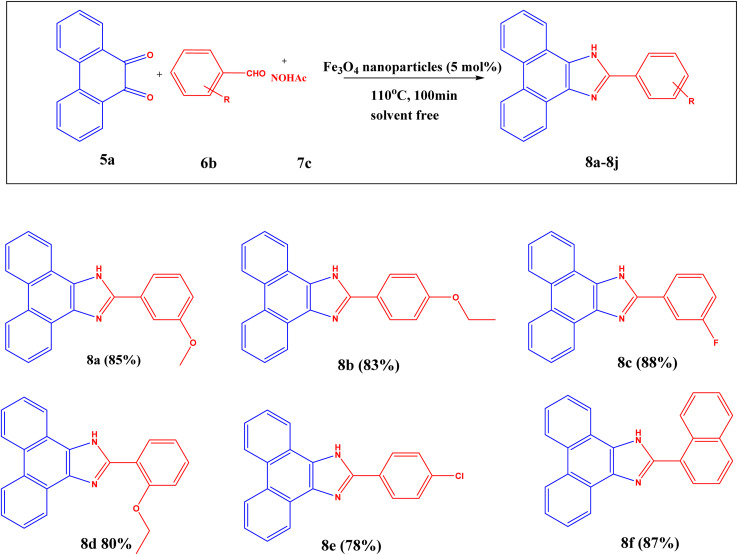
Reaction conditions: 9,10-phenanthrenequinone (1 eq.), aldehyde (1.25 equivalents), NH_4_OAc (3 _equivalents_), Fe_3_O_4_ nanoparticles (5 mol% respect to the 9,10-phenanthrenequinone), solvent-free at 110 °C for 100 min, yield (%) isolated yield after separation by column chromatography.

The optimization for synthesizing substituted 2-phenyl-1*H*-phenanthro[9,10-*d*] imidazole reaction is as follows: 9,10-phenanthrenequinone (1 equivalent), substituted aldehyde (1.25 equivalents), NH_4_OAc (3 equivalents), and Fe_3_O_4_ nanoparticles (5 mol%) are allowed at 110 °C for 100 min as given in Table S4† (entry 15) with excellent yield (Scheme 2).

## 4 Conclusions

l-Tartaric acid and *o*-phenylenediamine were successfully combined to create the NCQD, using a solvothermal technique. The resulting NCQD's many functional groups exhibited intense green fluorescence with high quantum yields (40.3%). This NCQD can be used in the bioimaging of HeLa cells and in the fluorescent detection of OCl^−^ ions. To synthesize the catalyst, the NCQD was also employed in the production of Fe_3_O_4_ nanoparticles, where it served as a reducing and stabilizing agent. Further, the catalyst was subjected to various characterizations, which confirmed the presence of Fe_3_O_4_ nanoparticles. The size of the catalyst was 11 nm, and it had a high surface area of 67.360 m^2^ g^−1^. On account of these characteristics, Fe_3_O_4_ nanoparticles can be used as a catalyst to create various organic molecules in a solvent-free condition. These nanocatalysts produced outstanding yields when they were used for preparing substituted imidazoles.

## Data availability

The data supporting this article have been included as part of the ESI.†

## Conflicts of interest

There is no conflict of declaration.

## Supplementary Material

RA-014-D4RA06474G-s001
