# Supplementary material for: N-doped carbon quantum dots for the selective detection of OCl− ions, bioimaging, and the production of Fe3O4 nanoparticles utilized in the synthesis of substituted imidazole
Source: RSC Adv. 2024 Nov 6;14(48):35448–59. doi: 10.1039/d4ra06474g (PMC11538961; doi:10.1039/d4ra06474g)
Supplement: RA-014-D4RA06474G-s001 [file RA-014-D4RA06474G-s001.pdf]

Supplementary Information (SI) for RSC Advances.
This journal is © The Royal Society of Chemistry 2024

N-doped carbon quantum dots for the selective detection of OCl^- ion, bioimaging, and the production of Fe_3O_4 nanoparticles utilized in the synthesis of substituted imidazole

Namrata Priyadarshini Hota and Sathiyanarayanan Kulathu Iyer*

Department of Chemistry, School of Advanced Sciences, Vellore Institute of Technology, Vellore-632 014, India. *E-mail: sathiyanarayanank@vit.ac.in

Table of Contents:

1. Quantum Yield Measurement———————————————————————— **S1**

2. Different wavelength and PH of NCQD——————————————————— Figure **S1**

3. Time response of NCQD with OCl^- and HRTEM image of NCQD -------------------------Figure **S2**

4. Comparison Table of different sensors for OCl^- detection -------------------- Table **S1**

5. Comparison table of catalytic activity of Fe_3O_4 NPs imidazole (4) Reaction --------Table **S2**

6. Comparison table of catalytic activity of Fe_3O_4 NPs imidazole (8) Reaction -------- Table **S3**

7. Optimization Table for imidazole Reactions (4) ------------------------ Table **S4**

7. Optimization Table for imidazole Reactions (8) ------------------------- Table **S5**

8. Recyclability of Fe_3O_4NPs ———————————————————————— **S2,**

9. Recyclability of Fe_3O_4NPs ————————————————————————**Figure S3**

10. H^1 and C^{13} NMR analysis of imidazole Reactions (4) ------------------------------------**S3**

11. H^1 and C^{13} NMR analysis of imidazole Reactions (8) ------------------------------------**S4**

12. H^1 NMR spectra and C^{13} of the products ---------------------------- **S5**

S1 Quantum Yield Measurement:

Using quinine sulfate (φ= 0.54 in 0.1M H_2SO_4) as a standard reference, we determined the quantum yield of carbon dots. Using the following formula, the quantum yield was determined.

$$\Phi_{CQD} = \Phi_{R} \times \frac{ICQD}{IR} \times \frac{AR}{ACQD} \times \frac{ŋ2CQD}{ŋ2R}$$

The carbon dot and reference are denoted by CQD and R, respectively, in the equation above. " ŋ " indicates the refractive index of the solvent medium (ethanol has a refractive index of 1.37), "I" stands for the integrated fluorescence intensity, and "A" for the absorbance value at the exiting wavelength. After calculating every value, the quantum Yield was found to be **40.5%**.

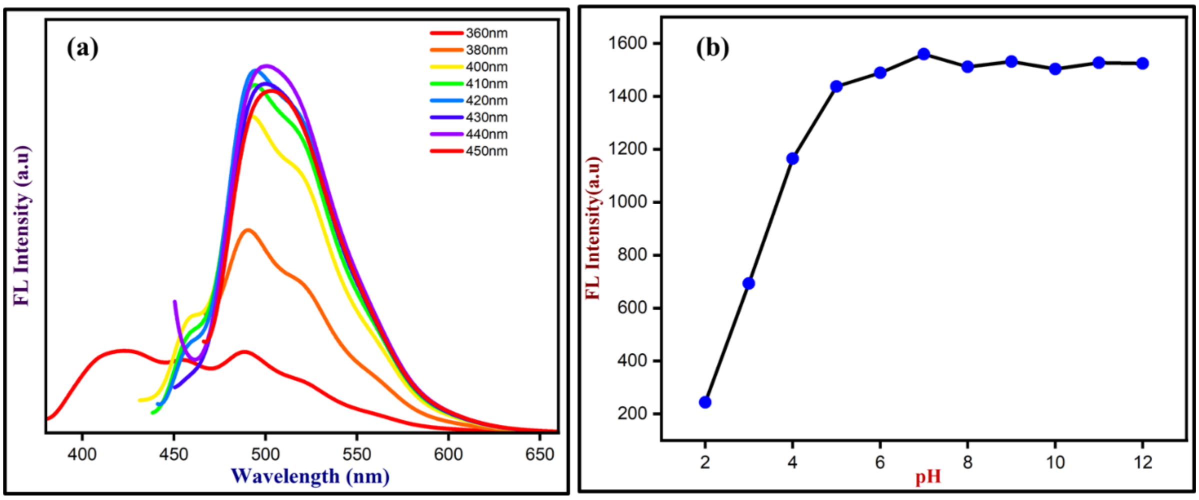

Figure S1. (a) represents the NCQD with different wavelengths, and (b) pH studies of the NCQD calibration plot of NCQD with varying concentrations of OCl- ion (0-8µm).

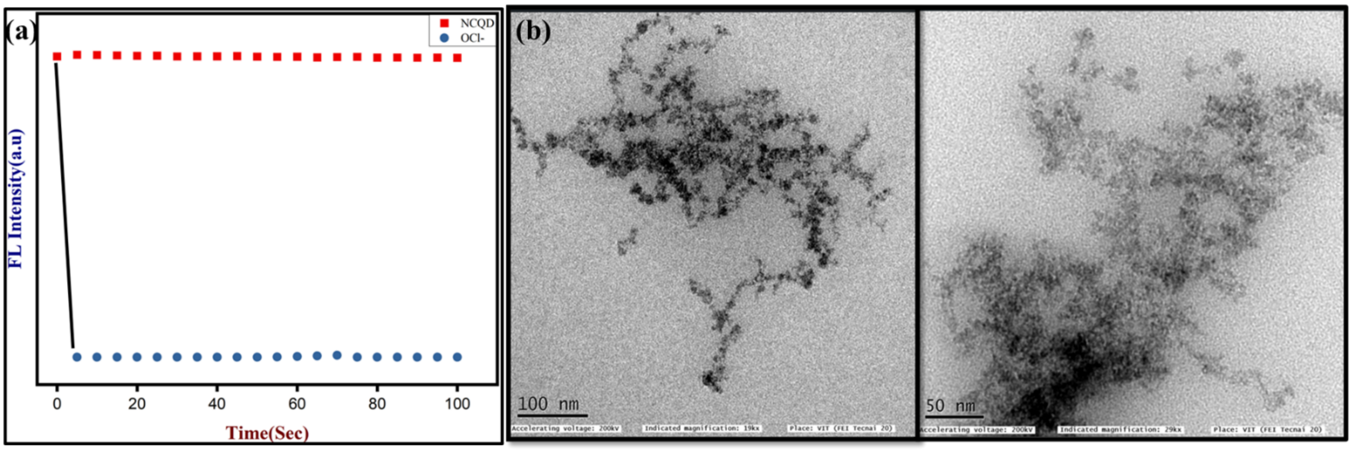

Figure S2. (a) Time response of NCQD with OCl^-, and (b) calibration plot of NCQD with different concentrations of OCl^- ion (0-8μm).

Table S1: Comparison Table of different sensors for OCl^- detection:

**Sensing Probe for OCl^-**	**Linear range (μm)**	**Lod (μm)**	**Ref.**
CA and EDA/N-doped CQDs	1.0–10.0	0.43	[*Spectrochimica Acta Part A: Molecular and Biomolecular Spectroscopy*, *279*, 121456]
MoO_3 NPs-G-CDs	0.010–200	0.068	[*Spectrochimica Acta Part A: Molecular and Biomolecular Spectroscopy*, *279*, 121456]
Citric acid, GSH	0.1–0.8	0.016	[*Spectrochimica Acta Part A: Molecular and Biomolecular Spectroscopy*, *242*, 118721]
Red Pepper-based CQD	0.1 -300	0.06	[*Analyst*, *138*(21), 6551-6557]
ammonium citrate and urea	10-150	1.82	[*Analytical and Bioanalytical Chemistry*, *414*(8), 2651-2660]
**L- Tartaric acid and o-phenylenediamine-based CQD**	**1-8**	**0.04**	**This work**

Table S2: Comparison table of catalytic activity of Fe_3O_4 NPs for imidazole (4) Reaction:

**Entry**	**Catalyst**	**Solvent**	**Temperature (°C)**	**Time (min)**	**Yield (%)**	**Ref.**
**1**	Cr_2O_3 nanoparticle	H_2O	MW, 200–500 W	6	97	[*Scientific Reports*, *12*(1), 19942]

**2**	[P4-VP]-Fe_3O_4	Solvent-free	100	35	91	[*Research on Chemical Intermediates*, *44*, 6995-7011]
**3**	Cu2O/ Fe_3O_4@guarana	EtOH	70	20	97	[*Inorganic Chemistry Communications*, *125*, 108465]
**4**	Fe_3O_4/SiO_2/urea	EtOH	Reflux	50	90	[*Catalysis Communications*, *69*, 29-33]
**5**	SA–MNPs	Dry petroleum ether	100, Microwave	35	88	[*Journal of Chemical Sciences*, *125*, 835-841]
**6**	**Fe_3O_4 nanoparticles**	**Solvent-free**	**100**	**90**	**94**	**This Work**

Table S3: Comparison table of catalytic activity of Fe_3O_4 NPs for imidazole (8) Reaction:

**Entry**	**Catalyst**	**Solvent**	**Temperature (°C)**	**Time (min)**	**Yield (%)**	**Ref.**
1	Ruthenium (II) catalyst	H_2O	100	5	66	[*89(9), 6016-6026*]
2	Iodine	EtOH	Reflux	60	80	[*Chinese Chemical Letters, 20(1), 5-8*]
3	TiO_2	Solvent-free	120	45	88	[*39(1), 142-154.*]
	**Fe_3O_4 nanoparticles**	**Solvent-**	**110**	**100**	**88**	**This**

	free			Work

Table S4: Optimization table for substituted imidazole (4) Reaction:

Entry	Solvent	Nitrogen Source	Temp (℃)	Fe_3O_4(mol%)	Time(min)	Yield (%)
1	H_2O	NH_4OAc	100	3.3	90	45
2	DMF	NH_4OAc	100	3.3	90	Trace
3	Acetone	NH_4OAc	100	3.3	90	Trace
4	Acetonitrile	NH_4OAc	100	3.3	90	40
5	Toluene	NH_4OAc	100	3.3	90	20
6	EtOH	NH_4OAc	100	3.3	90	85
7	EtOH	NH_4OAc	100	3.3	90	80
8	THF	NH_4OAc	100	3.3	90	35
9	EtOH	NH_4OAc	100	3.3	90	75
10	EtOH	NH_4OAc	100	-	90	41
11	NEAT	NH_4OAc	100	-	90	40
12	NEAT	NH_4OAc	100	2	90	88
13	NEAT	NH_4OAc	100	4	90	96
14	NEAT	NH_4OAc	100	5	90	96
15	**NEAT**	**NH_4OAc**	**100**	**3.3**	90	**96**

Table S5: Optimization table for substituted imidazole (8) Reaction:

Entry	Solvent	Nitrogen Source	Temp (℃)	Fe_3O_4(mol%)	Time(min)	Yield (%)
**1**	THF	NH_4OAc	110	5	100	Trace
**2**	Acetone	NH_4OAc	110	5	100	20
**3**	H_2O	NH_4OAc	110	5	100	38
**4**	EtOH: H_2O	NH_4OAc	110	5	100	70
**5**	Acetonitrile	NH_4OAc	110	5	100	20
**6**	EtOH	NH_4OAc	110	5	100	78
**7**	EtOH	NH_4OAc	110	6	100	80
**8**	Tolune	NH_4OAc	110	5	100	35
**9**	EtOH	NH_4OAc	110	5	100	75
**10**	EtOH	NH_4OAc	110	-	100	39
**11**	NEAT	NH_4OAc	110	-	100	53
**12**	NEAT	NH_4OAc	110	4	100	82
**13**	NEAT	NH_4OAc	110	6	100	89
**14**	NEAT	NH_4OAc	110	5.5	100	88
**15**	**NEAT**	**NH_4OAc**	110	**5**	100	**88**

S2: Recyclability of Fe_3O_4 nanoparticles:

After the reaction was complete, the reaction mixture was allowed to cool to room temperature and moved to a centrifuge tube. After centrifuging for 10 minutes at 4000 RPM, the Fe_3O_4 NPs that had settled to the bottom of the reaction mixture were cleaned several times with water, ethanol, and acetone. They were then ground into a fine powder and allowed to dry in an oven. After that, the reactions were conducted using the catalyst once more till 4 cycles.

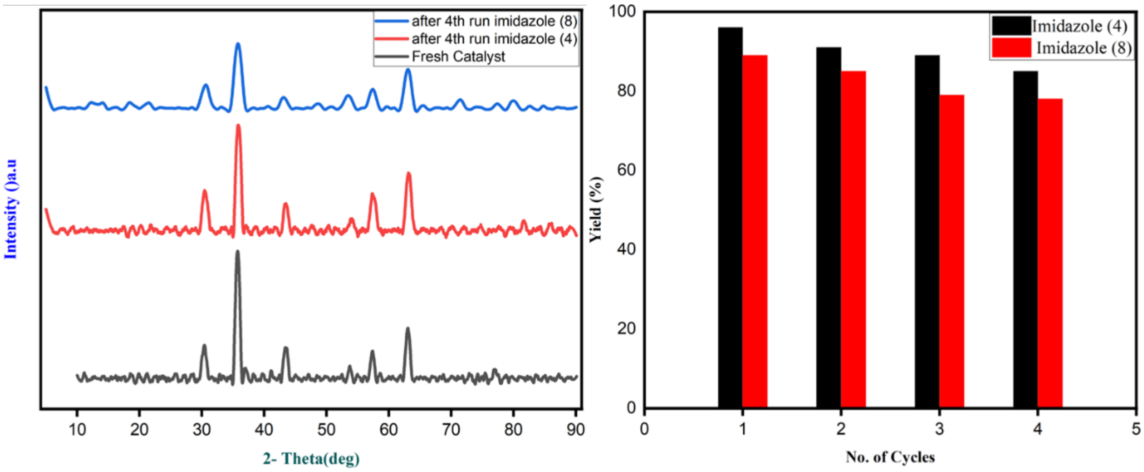

Figure S3: Recyclability study of the Fe_3O_4 NPs

S3: H^1 and C^{13} NMR analysis result of substituted Imidazole (4) Reaction:

1. 4,5-diphenyl-2-(p-tolyl)-1H-imidazole (4a): Off -White Powder. H^1 NMR (400 MHZ, DMSO), δ (PPM) = 12.59 (s, 1H), 7.90 (d, J= Hz, 2H), 7.52 (m, 6H), 7.30 (t, 8 Hz, 6H), 2.36 (s, 3H). C13 NMR (100 MHz, $CDCl_3$) δ 21.39, 125.21, 126.48, 126.56, 127.37, 127.82, 128.17, 128.59, 129.59, 138.85, 146.21.
2. 4-(4,5-diphenyl-1H-imidazol-2-yl) benzonitrile (4f): white Powder.H^1 NMR (400 MHZ, DMSO), δ (PPM) = 13.01 (s, 1H), 8.26 (d, J=8Hz, 2H), 7.96 (d, 8.4Hz, 2H), 7.56 (m, 4H), 7.49 (t, J=7.4Hz, 2H), 7.43(t, J= 8Hz, 1H), 7.34(t, J= 7.2Hz, 2H), 7.27 (t, J= 7.2Hz, 1H).C13 NMR (100 MHz, $CDCl_3$) δ 117.8, 118.69, 125.41, 127.84, 128.78, 132.76, 133.78.
3. 4,5-diphenyl-2-(o-tolyl)-1H-imidazole (4b): White powder. H^1 NMR (400 MHZ, DMSO), δ (PPM) = 12.46 (s, 1H), 7.72 (t, J= 8.4 Hz, 1H), 7.56 (d, J= 7.2 Hz, 2H), 7.52 (d, J = 7.2 Hz, 2H), 7.45 (t, J= 7.2 Hz, 2H), 7.38 (m, 6H), 7.23 (t, J= 7.2 Hz, 1H),2.64 (s, 3H). C13 NMR (100 MHz, $CDCl_3$) δ 21.19, 126.06, 127.76, 128.65, 128.87, 129.74, 131.35, 136.43, 146.31.
4. 2-(4-methoxyphenyl)-4,5-diphenyl-1H-imidazole (4c): White Powder. H^1 NMR (400 MHZ, DMSO), δ (PPM) = 12.50 (s, 1H), 8.03 (d, *J*= 8.4Hz, 2H), 7.53 (d, *J*=8 Hz, 4H), 7.37 (s, 6H), 7.96 (d, J= 8.8 Hz, 2H), 3.82 (s, 3H). C13 NMR (100 MHz, $CDCl_3$) δ 55.39, 114.32, 122.73, 126.75, 127.37, 127.78, 128.61, 146.07, 160.21.
5. 2-(2-methoxyphenyl)-4,5-diphenyl-1H-imidazole(4d): white Powder. H^1 NMR (400 MHZ, DMSO), δ (PPM) = 11.89 (s, 1H), 8.05 (m, 1H), 7.53 (m, 8H), 7.31 (t, J= 7.2 Hz, 2H), 7.23 (d, J= 8Hz, 1H), 7.18 (d, J= 8Hz, 1H), 7.09 (t, J= 7.2 Hz, 1H), 3.92 (s, 3H). C13 NMR (100 MHz, $CDCl_3$) δ 55.91, 111.21, 118.18, 121.71, 127.81, 128.66, 129.53, 144.65, 155.74.
6. 2-(4-nitrophenyl)-4,5-diphenyl-1H-imidazole (4e): Yellow Powder. H^1 NMR (400 MHZ, DMSO), δ (PPM) = 13.16 (s, 1H), 8.37 (m, 4H), 7.57 (m, 4H), 7.49 (t, J= 8 Hz, 2H), 7.43 (d, J= 6.8 Hz, 1H), 7.35 (t, J= 7.2 Hz, 2H1), 7.28 (t, J= 8Hz, 1H). C13 NMR (100 MHz, $CDCl_3$) δ 124.41, 125.51, 127.88, 128.01, 128.77, 135.52, 143.47, 147.41.
7. 4-(4,5-diphenyl-1H-imidazol-2-yl) benzonitrile (4f), white powder. H^1 NMR (400 MHZ, DMSO), δ (PPM) = 13.01 (s, 1H), 8.26 (d, j= 8 Hz,2H), 7.96 (d, J= 8.4 Hz, 2H), 7.56 (m, 4H), 7.49 (t, J= 7.2 Hz), 7.43 (d, J= 7.2 Hz, 1H), 7.34 (t, J= 8Hz, 2H), 7.27 (t, J= 7.2 Hz, 1H). C13 NMR (100 MHz, $CDCl_3$) δ
8. 1-(4,5-diphenyl-1H-imidazol-2-yl) naphthalen-2-ol (4g): Yellow Powder. H1 NMR (400 MHZ, DMSO), δ (PPM)= 12.5-11.5 (2H), 8.19 (d, J= 8Hz, 1H), 7.91 (t, J= 8.8Hz, 2H), 7.57 (d, J=

7.2Hz, 4H), 7.51 (t, J= 7.6 Hz, 1H), 7.41 (m, 5H), 7.34 (m, 3H). C13 NMR (100 MHz, $CDCl_3$) δ 115.87, 116.09, 126.24, 127.21, 127.55, 127.80, 128.65, 145.18, 161.89.

9. 2-(4-fluorophenyl)-4,5-diphenyl-1H-imidazole (4h), Brown solid. H^1 NMR (400 MHZ, DMSO), δ (PPM) = 12.69 (s, 1H), 8.14 (m, 2H), 7.55 (d, J= 7.2 Hz, 2H), 7.51 (t, J=8Hz, 2H), 7.47 (t, J= 7.2Hz, 2H), 7.40 (d, J= 7.2Hz, 1H), 7.35 (m, 4H), 7.24 (t, J= 7.6Hz, 1H). 2-(5-methylfuran-2-yl)-4,5-diphenyl-1H-imidazole (4i): brown powder. H^1 NMR (400 MHZ, DMSO), δ (PPM) = 12.68 (s, 1H), 7.52 (t, J=7.8 Hz, 2H), 7.48 (d, J=6.8Hz, 2H), 7.44 (t, J= 7.2 Hz, 2H), 7.37 (t, J= 4.8 Hz, 1H), 7.31 (t, J= 7.2Hz, 2H), 7.23 (t, J= 7.2Hz, 1H), 6.86 (d, J= 4Hz, 1H), 6.25 (t, J= 4Hz, 1H), 2.38 (s,3H).

10. 2-(4,5-diphenyl-1H-imidazol-2-yl)-6-methoxy phenol (4j): white powder. H^1 NMR (400 MHZ, DMSO), δ (PPM) = 13.05 (s, 1H), 13.01 (s, 1H), 7.65 (t, J= 4 Hz, 1 H), 7.54 (m, 5H), 7.47 (t, J= 6.8 Hz, 2H), 7.37 (t, J= 7.6 Hz, 2H), 7.29 (t, J= 7.2 Hz, 1H), 7.00 (d, J= 8Hz, 1H), 6.90 (t, j= 8Hz, 1H), 3,82 (s, 3H). C13 NMR (100 MHz, $CDCl_3$) δ 56.15, 112.07, 112.95, 115.59, 118.84, 127.72, 128.74, 145.54, 147.02, 148.89.

11. 2-(3-phenoxyphenyl)-4,5-diphenyl-1H-imidazole (4k): white powder. H^1 NMR (400 MHZ, DMSO), δ (PPM) = 12.74 (s, 1H), 7.90 (d, J= 8Hz 1H), 7.78 (t, J= 4Hz, 1H), 7.75 (m, 4H), 7.45 (m, 5H), 7.31 (t, J= 7.2 Hz, 2H), 7.24 (t, J= 7.2 Hz, 1H), 7.18 (t, J= 7.6 Hz, 1H), 7.08 (d, J= 7.6 Hz, 2H), 7.04 (m, 1H), C13 NMR (100 MHz, $CDCl_3$) δ 115.64, 118.96, 119.32, 120.24, 123.48, 127.82, 128.74, 129. 87, 130,38, 131.38, 131.69, 157.08, 157.80.

S4: H^1 NMR analysis result of imidazole (8):

1. 2-(3-methoxyphenyl)-1H-phenanthro[9,10-d] imidazole (8a): yellow solid. H^1 NMR (400 MHZ, DMSO), δ (PPM) = 13.42 (s, 1H), 8.89 (m, 2H), 8.61 (m, 2H), 7.92 (t, *J*= 8Hz, 2H), 7.78 (m, 2H), 7.67 (m, 2H), 7.54 (t, *J*=8HZ, 1H), 7.09 (m, 1H), 3.93 (s, 3H). C13 NMR (100 MHz, $CDCl_3$) δ

2. 2-(4-ethoxy phenyl)-1H-phenanthro[9,10-d] imidazole (8b): light brown powder. H^1 NMR (400 MHZ, DMSO), δ (PPM) = 13.29 (s, 1H), 8.86 (d, *J*=8Hz, 2H), 8.56 (s, 2H), 8.25 (d, *J*= 8,8Hz, 2H), 7.75 (t, J= 6.8Hz, 2H), 7.64 (m, 2H), 7.16(d, *J*= 8.8Hz, 2H), 4.16 (m, 2H), 1.40 (t, *J*= 6.8Hz, 3H). C13 NMR (100 MHz, $CDCl_3$) δ

3. 2-(3-fluorophenyl)-1H-phenanthro[9,10-d] imidazole (8c): yellow solid. H^1 NMR (400 MHZ, DMSO), δ (PPM) = 13.54 (s, 1H), 8.89 (m, 2H), 8.61 (d, *J*= 7.8Hz, 1H), 8.55 (d, *J*= 8Hz, 1H), 8.19 (d, *J*= 8Hz, 1H), 8.12 (t, *J*=8.4Hz, 1H), 7.79 (m, 2H), 7.68 (m, 3H), 7.36 (m, 3H). C13 NMR (100 MHz, $CDCl_3$) δ

4. 2-(2-ethoxy phenyl)-1H-phenanthro[9,10-d] imidazole (8d): H1 NMR (400 MHZ, CDCl3), δ (PPM) = 11.40 (s,1H), 8.80 (m, 4H), 7.87 (d, *J*= 8Hz 1H), 7.73 (t, *J*= 6.8 Hz, 1H), 7.63 (t, *J*= 7.2, 3H), 7.38 (m, 1H), 7.19 (t, *J*= 7.6 Hz, 1H), 7.04 (d, *J*= 8Hz, 1H), 4.35 (m, 2H), 1.76 (t, *J*= 7.2 Hz, 3H).

5. 2-(4-chlorophenyl)-1H-phenanthro[9,10-d] imidazole (8e): white solid. H^1 NMR (400 MHZ, DMSO), δ (PPM) = 13.54 (s, 1H), 8.86 (s, 2H), 8.56 (s, 2H), 8.34 (d, *J*= 8.8 Hz), 7.75 (m, 6H). C13 NMR (100 MHz, $CDCl_3$) δ

6. 2-(naphthalen-1-yl)-1H-phenanthro[9,10-d] imidazole (8f): Yellow Powder. H^1 NMR (400 MHZ, DMSO), δ (PPM) = 13.66 (s, 1H), 9.20 (d, *J*= 8.4Hz, 1H), 8.90 (d, *J*= 6.8Hz, 2H), 8.65 (s, 1H), 8.59 (s, 1H), 8.15 (t, *J*=7.6 Hz, 2H), 8.09 (d, *J*= 8Hz, 1H), 7.78 (m, 3H), 7.70 (m, 4H). C13 NMR (100 MHz, $CDCl_3$) δ

S5: ^{1}H NMR spectra (400 MHz) of the products:

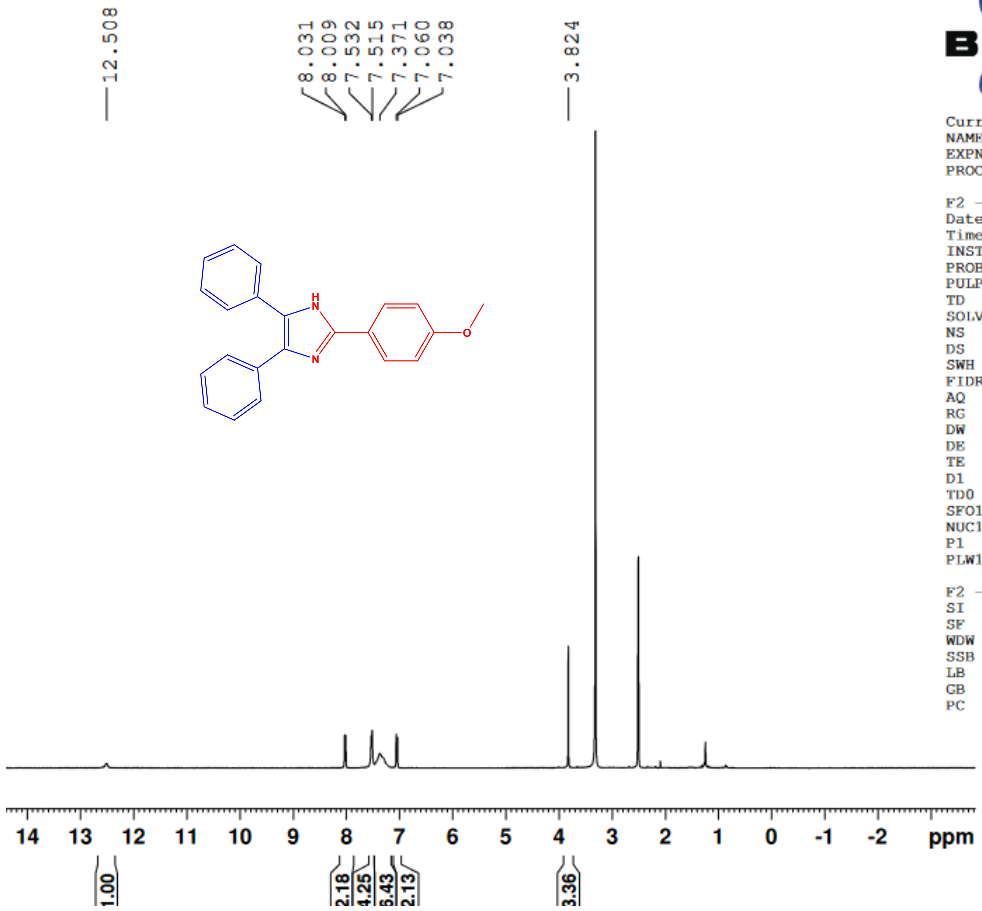

BRUKER

Current Data Parameters

NAME	Dr.SYN060524
EXPNO	4
PROCNO	1

F2 - Acquisition Parameters

Date_	20240506
Time	14.30 h
INSTRUM	spect
PROBHD	Z108618_0505 (
PULPROG	zg30
TD	65536
SOLVENT	DMSO
NS	32
DS	2
SWH	8012.820 Hz
FIDRES	0.244532 Hz
AQ	4.0894465 sec
RG	199.6
DW	62.400 usec
DE	6.50 usec
TE	307.5 K
D1	1.00000000 sec
TD0	1
SFO1	400.2604716 MHz
NUC1	1H
P1	15.00 usec
PLW1	15.21399975 W

F2 - Processing parameters

SI	65536
SF	400.2580000 MHz
WDW	EM
SSB	0
LB	0.30 Hz
GB	0
PC	1.00

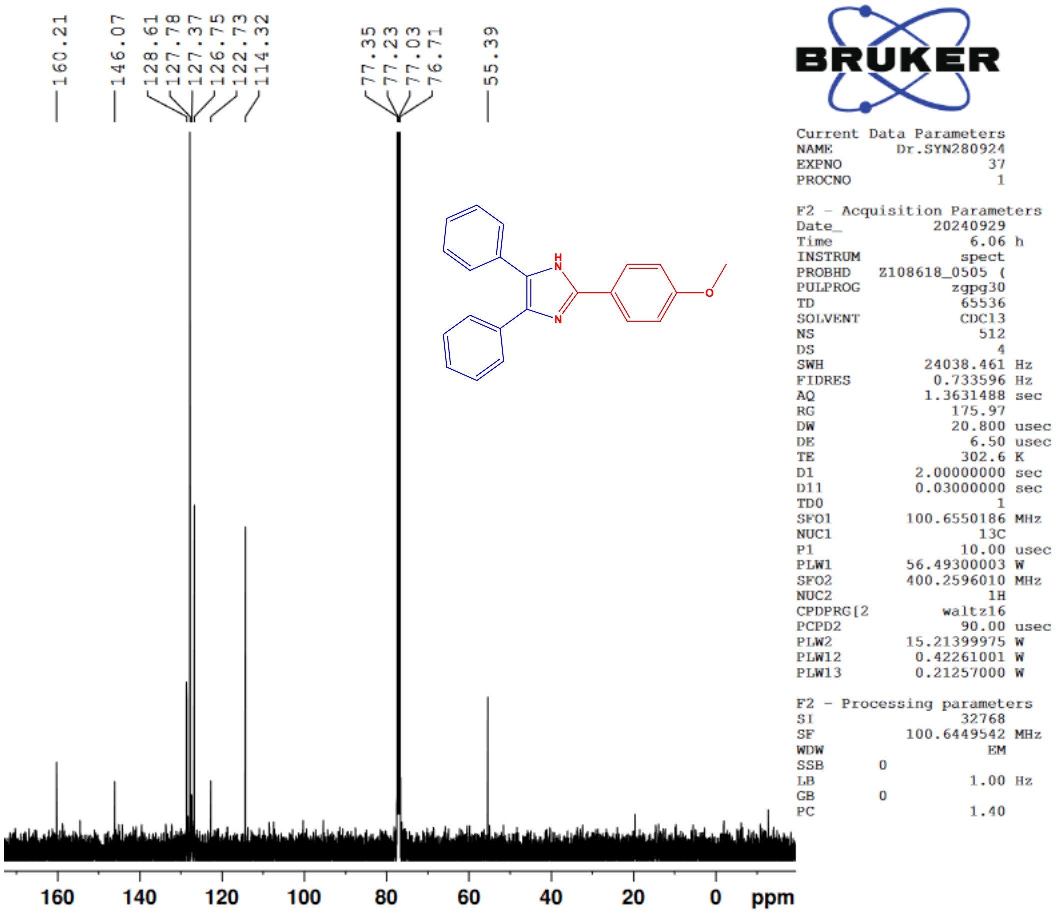
160.21
146.07
128.61
127.78
127.37
126.75
122.73
114.32
77.35
77.23
77.03
76.71
55.39
H
N
N
O
160 140 120 100 80 60 40 20 0 ppm
BRUKER
Current Data Parameters
NAME Dr.SYN280924
EXPNO 37
PROCNO 1
F2 - Acquisition Parameters
Date_ 20240929
Time 6.06 h
INSTRUM spect
PROBHD Z108618_0505 (
PULPROG zgpg30
TD 65536
SOLVENT CDCl3
NS 512
DS 4
SWH 24038.461 Hz
FIDRES 0.733596 Hz
AQ 1.3631488 sec
RG 175.97
DW 20.800 usec
DE 6.50 usec
TE 302.6 K
D1 2.00000000 sec
D11 0.03000000 sec
TD0 1
SFO1 100.6550186 MHz
NUC1 13C
P1 10.00 usec
PLW1 56.49300003 W
SFO2 400.2596010 MHz
NUC2 1H
CPDPRG[2 waltz16
PCPD2 90.00 usec
PLW2 15.21399975 W
PLW12 0.42261001 W
PLW13 0.21257000 W
F2 - Processing parameters
SI 32768
SF 100.6449542 MHz
WDW EM
SSB 0
LB 1.00 Hz
GB 0
PC 1.40

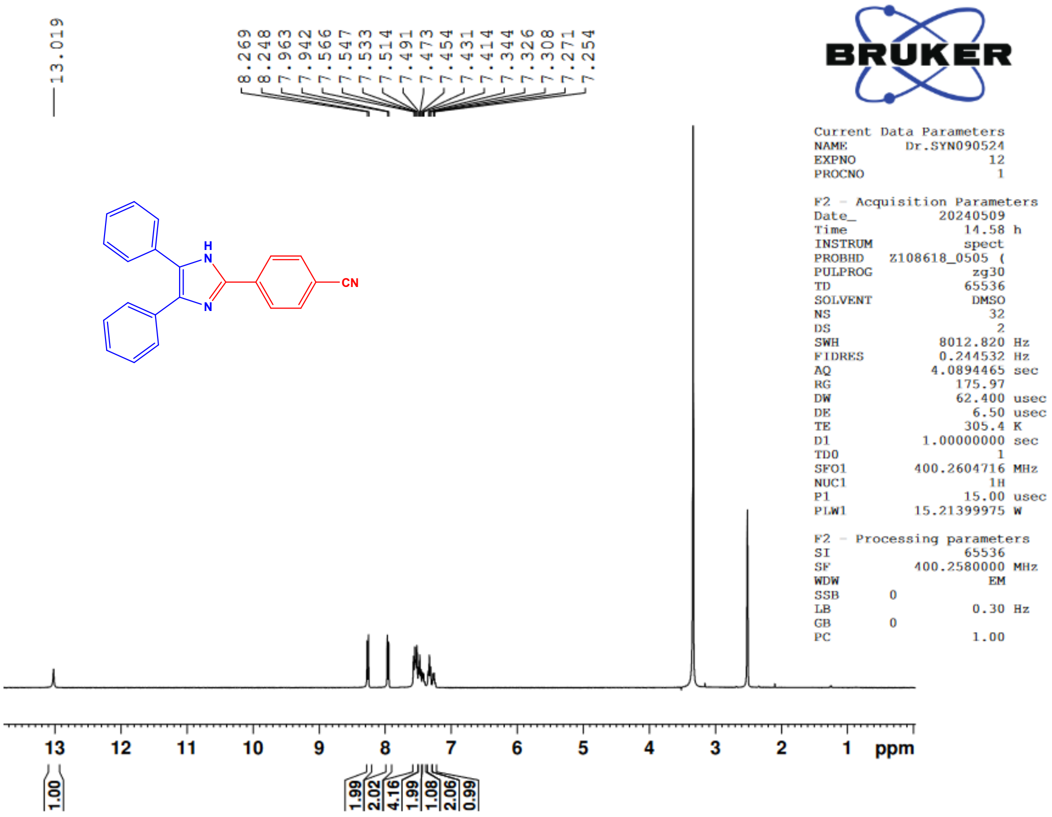
13.019
8.269
8.248
7.963
7.942
7.566
7.547
7.533
7.514
7.491
7.473
7.454
7.431
7.414
7.344
7.326
7.308
7.271
7.254
H
N
N
CN
13 12 11 10 9 8 7 6 5 4 3 2 1 ppm
1.00
1.99
2.02
4.16
1.99
1.08
2.06
0.99
BRUKER
Current Data Parameters
NAME Dr.SYN090524
EXPNO 12
PROCNO 1
F2 - Acquisition Parameters
Date_ 20240509
Time 14.58 h
INSTRUM spect
PROBHD Z108618_0505 (
PULPROG zg30
TD 65536
SOLVENT DMSO
NS 32
DS 2
SWH 8012.820 Hz
FIDRES 0.244532 Hz
AQ 4.0894465 sec
RG 175.97
DW 62.400 usec
DE 6.50 usec
TE 305.4 K
D1 1.00000000 sec
TD0 1
SFO1 400.2604716 MHz
NUC1 1H
P1 15.00 usec
PLW1 15.21399975 W
F2 - Processing parameters
SI 65536
SF 400.2580000 MHz
WDW EM
SSB 0
LB 0.30 Hz
GB 0
PC 1.00

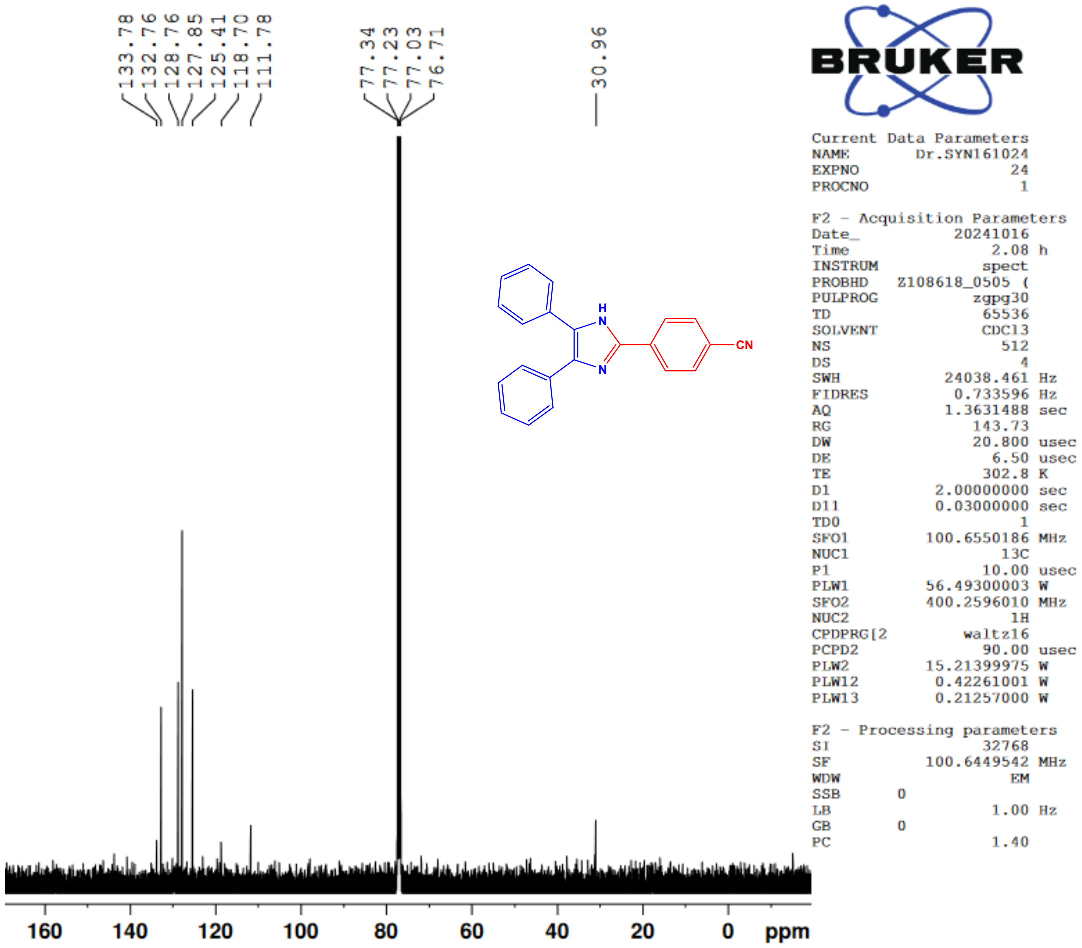
133.78
132.76
128.76
127.85
125.41
118.70
111.78
77.34
77.23
77.03
76.71
30.96
H
N
N
CN
160 140 120 100 80 60 40 20 0 ppm
BRUKER
Current Data Parameters
NAME Dr.SYN161024
EXPNO 24
PROCNO 1
F2 - Acquisition Parameters
Date_ 20241016
Time 2.08 h
INSTRUM spect
PROBHD Z108618_0505 (
PULPROG zgpg30
TD 65536
SOLVENT CDCl3
NS 512
DS 4
SWH 24038.461 Hz
FIDRES 0.733596 Hz
AQ 1.3631488 sec
RG 143.73
DW 20.800 usec
DE 6.50 usec
TE 302.8 K
D1 2.00000000 sec
D11 0.03000000 sec
TD0 1
SFO1 100.6550186 MHz
NUC1 13C
P1 10.00 usec
PLW1 56.49300003 W
SFO2 400.2596010 MHz
NUC2 1H
CPDPRG[2 waltz16
PCPD2 90.00 usec
PLW2 15.21399975 W
PLW12 0.42261001 W
PLW13 0.21257000 W
F2 - Processing parameters
SI 32768
SF 100.6449542 MHz
WDW EM
SSB 0
LB 1.00 Hz
GB 0
PC 1.40

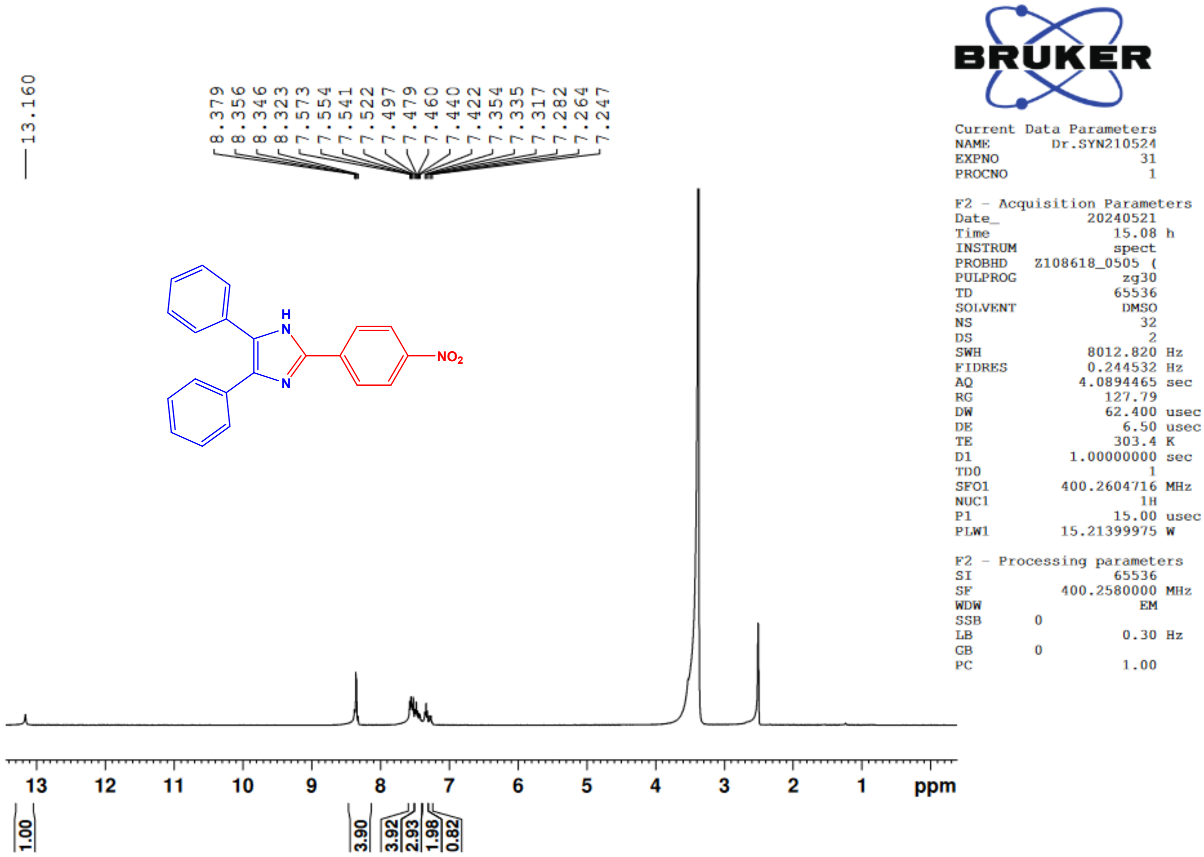
13.160
8.379
8.356
8.346
8.323
7.573
7.554
7.541
7.522
7.497
7.479
7.460
7.440
7.422
7.354
7.335
7.317
7.282
7.264
7.247
H
N
N
NO2
13 12 11 10 9 8 7 6 5 4 3 2 1 ppm
1.00
3.90
3.92
2.93
1.98
0.82
BRUKER
Current Data Parameters
NAME Dr.SYN210524
EXPNO 31
PROCNO 1
F2 - Acquisition Parameters
Date_ 20240521
Time 15.08 h
INSTRUM spect
PROBHD Z108618_0505 (
PULPROG zg30
TD 65536
SOLVENT DMSO
NS 32
DS 2
SWH 8012.820 Hz
FIDRES 0.244532 Hz
AQ 4.0894465 sec
RG 127.79
DW 62.400 usec
DE 6.50 usec
TE 303.4 K
D1 1.00000000 sec
TD0 1
SFO1 400.2604716 MHz
NUC1 1H
P1 15.00 usec
PLW1 15.21399975 W
F2 - Processing parameters
SI 65536
SF 400.2580000 MHz
WDW EM
SSB 0
LB 0.30 Hz
GB 0
PC 1.00

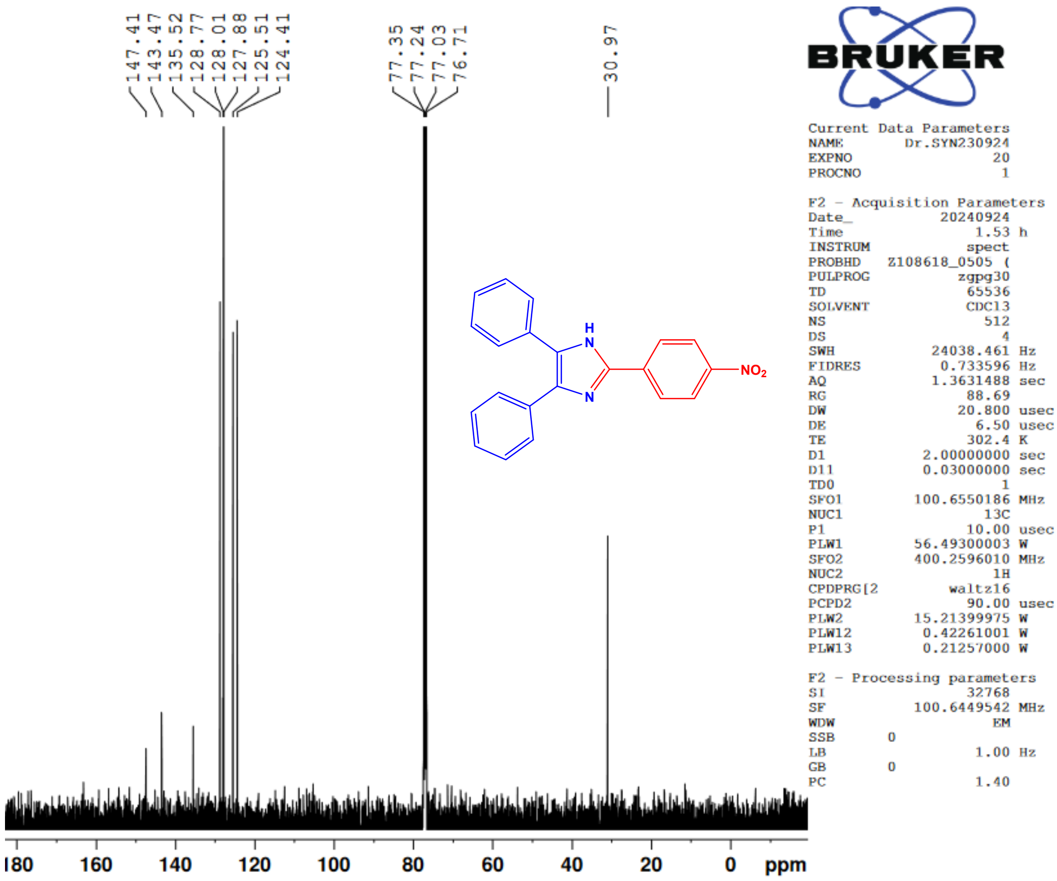
147.41
143.47
135.52
128.77
128.01
127.88
125.51
124.41
77.35
77.24
77.03
76.71
30.97
H
N
N
NO2
BRUKER
Current Data Parameters
NAME Dr.SYN230924
EXPNO 20
PROCNO 1
F2 - Acquisition Parameters
Date_ 20240924
Time 1.53 h
INSTRUM spect
PROBHD Z108618_0505 (
PULPROG zgpg30
TD 65536
SOLVENT CDCl3
NS 512
DS 4
SWH 24038.461 Hz
FIDRES 0.733596 Hz
AQ 1.3631488 sec
RG 88.69
DW 20.800 usec
DE 6.50 usec
TE 302.4 K
D1 2.00000000 sec
D11 0.03000000 sec
TD0 1
SFO1 100.6550186 MHz
NUC1 13C
P1 10.00 usec
PLW1 56.49300003 W
SFO2 400.2596010 MHz
NUC2 1H
CPDPRG[2 waltz16
PCPD2 90.00 usec
PLW2 15.21399975 W
PLW12 0.42261001 W
PLW13 0.21257000 W
F2 - Processing parameters
SI 32768
SF 100.6449542 MHz
WDW EM
SSB 0
LB 1.00 Hz
GB 0
PC 1.40
180 160 140 120 100 80 60 40 20 0 ppm

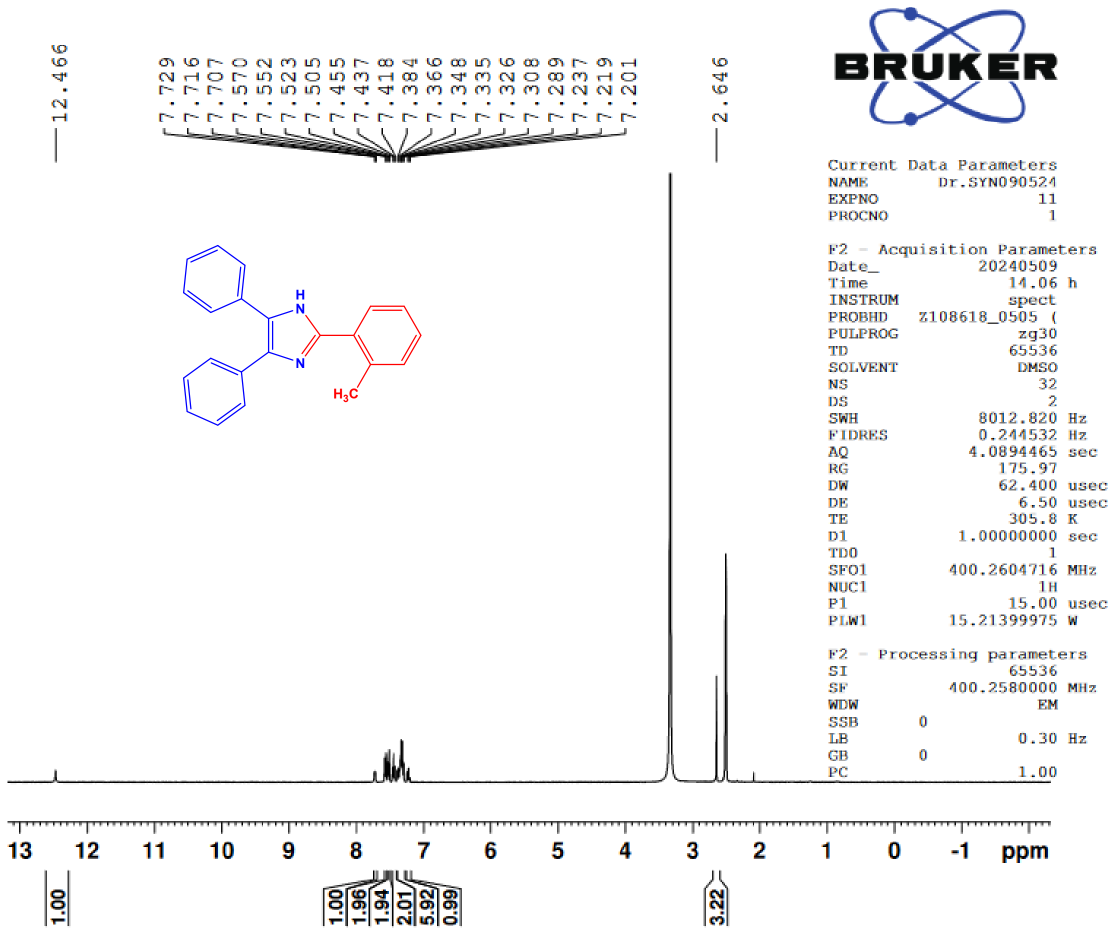
12.466
7.729
7.716
7.707
7.570
7.552
7.523
7.505
7.455
7.437
7.418
7.384
7.366
7.348
7.335
7.326
7.308
7.289
7.237
7.219
7.201
2.646
H
N
N
H3C
BRUKER
Current Data Parameters
NAME Dr.SYN090524
EXPNO 11
PROCNO 1
F2 - Acquisition Parameters
Date_ 20240509
Time 14.06 h
INSTRUM spect
PROBHD Z108618_0505 (
PULPROG zg30
TD 65536
SOLVENT DMSO
NS 32
DS 2
SWH 8012.820 Hz
FIDRES 0.244532 Hz
AQ 4.0894465 sec
RG 175.97
DW 62.400 usec
DE 6.50 usec
TE 305.8 K
D1 1.00000000 sec
TD0 1
SFO1 400.2604716 MHz
NUC1 1H
P1 15.00 usec
PLW1 15.21399975 W
F2 - Processing parameters
SI 65536
SF 400.2580000 MHz
WDW EM
SSB 0
LB 0.30 Hz
GB 0
PC 1.00
13 12 11 10 9 8 7 6 5 4 3 2 1 0 -1 ppm
1.00
1.00
1.96
1.94
2.01
5.92
0.99
3.22

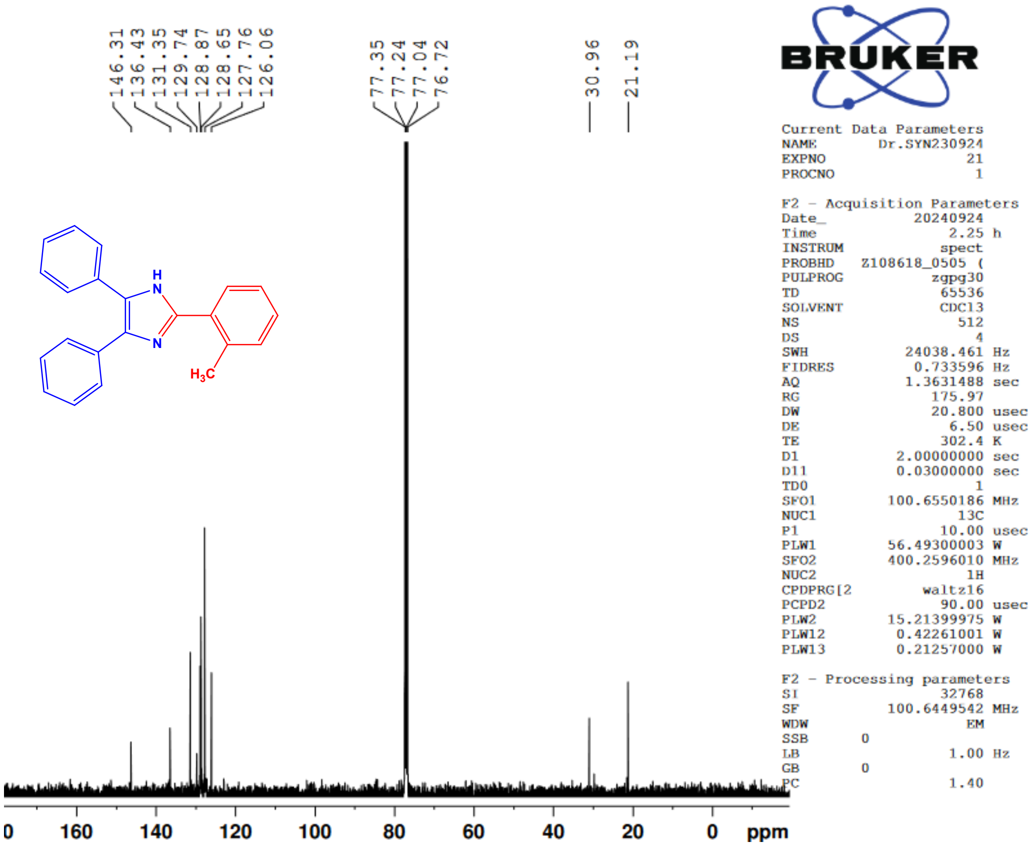
146.31
136.43
131.35
129.74
128.87
128.65
127.76
126.06
77.35
77.24
77.04
76.72
30.96
21.19
H
N
N
H_3C
BRUKER
Current Data Parameters
NAME Dr.SYN230924
EXPNO 21
PROCNO 1
F2 - Acquisition Parameters
Date_ 20240924
Time 2.25 h
INSTRUM spect
PROBHD Z108618_0505 (
PULPROG zgpg30
TD 65536
SOLVENT CDCl3
NS 512
DS 4
SWH 24038.461 Hz
FIDRES 0.733596 Hz
AQ 1.3631488 sec
RG 175.97
DW 20.800 usec
DE 6.50 usec
TE 302.4 K
D1 2.00000000 sec
D11 0.03000000 sec
TD0 1
SFO1 100.6550186 MHz
NUC1 13C
P1 10.00 usec
PLW1 56.49300003 W
SFO2 400.2596010 MHz
NUC2 1H
CPDPRG[2 waltz16
PCPD2 90.00 usec
PLW2 15.21399975 W
PLW12 0.42261001 W
PLW13 0.21257000 W
F2 - Processing parameters
SI 32768
SF 100.6449542 MHz
WDW EM
SSB 0
LB 1.00 Hz
GB 0
PC 1.40
0
160
140
120
100
80
60
40
20
0
ppm

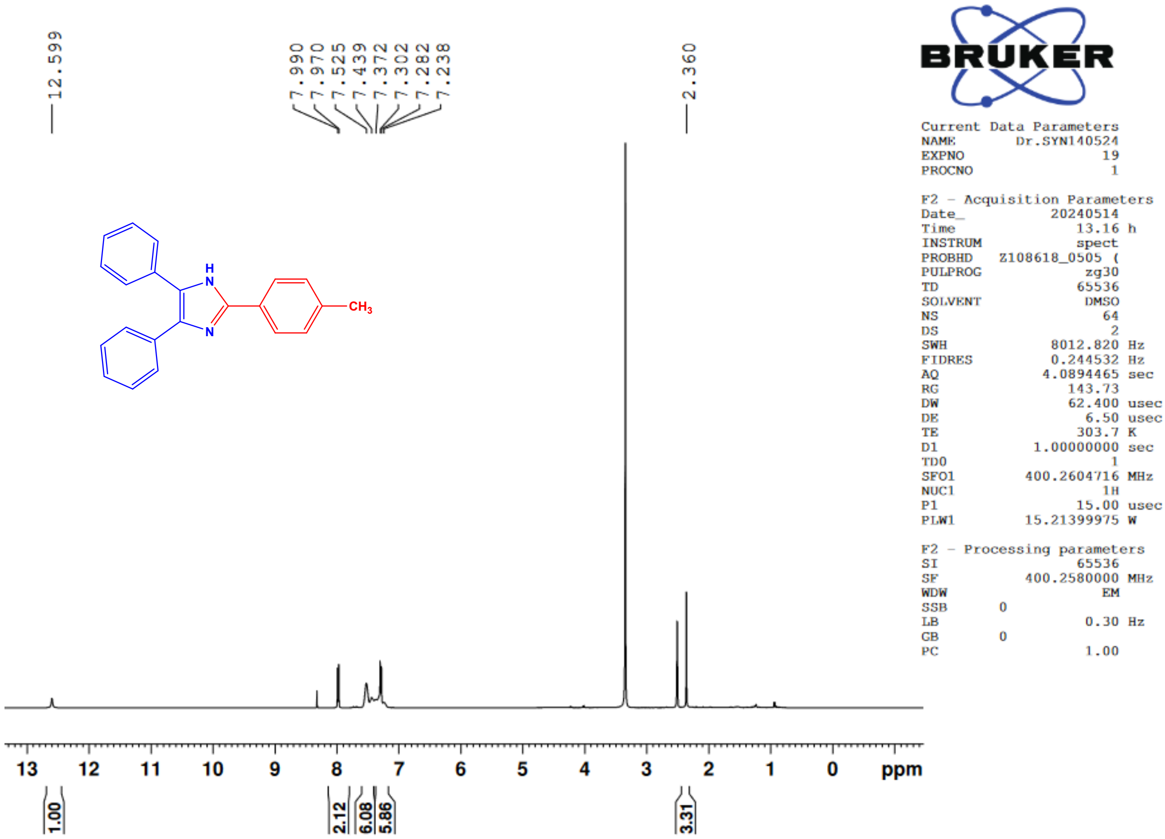
12.599
7.990
7.970
7.525
7.439
7.372
7.302
7.282
7.238
2.360
H
N
N
CH_3
BRUKER
Current Data Parameters
NAME Dr.SYN140524
EXPNO 19
PROCNO 1
F2 - Acquisition Parameters
Date_ 20240514
Time 13.16 h
INSTRUM spect
PROBHD Z108618_0505 (
PULPROG zg30
TD 65536
SOLVENT DMSO
NS 64
DS 2
SWH 8012.820 Hz
FIDRES 0.244532 Hz
AQ 4.0894465 sec
RG 143.73
DW 62.400 usec
DE 6.50 usec
TE 303.7 K
D1 1.00000000 sec
TD0 1
SFO1 400.2604716 MHz
NUC1 1H
P1 15.00 usec
PLW1 15.21399975 W
F2 - Processing parameters
SI 65536
SF 400.2580000 MHz
WDW EM
SSB 0
LB 0.30 Hz
GB 0
PC 1.00
13
12
11
10
9
8
7
6
5
4
3
2
1
0
ppm
1.00
2.12
6.08
5.86
3.31

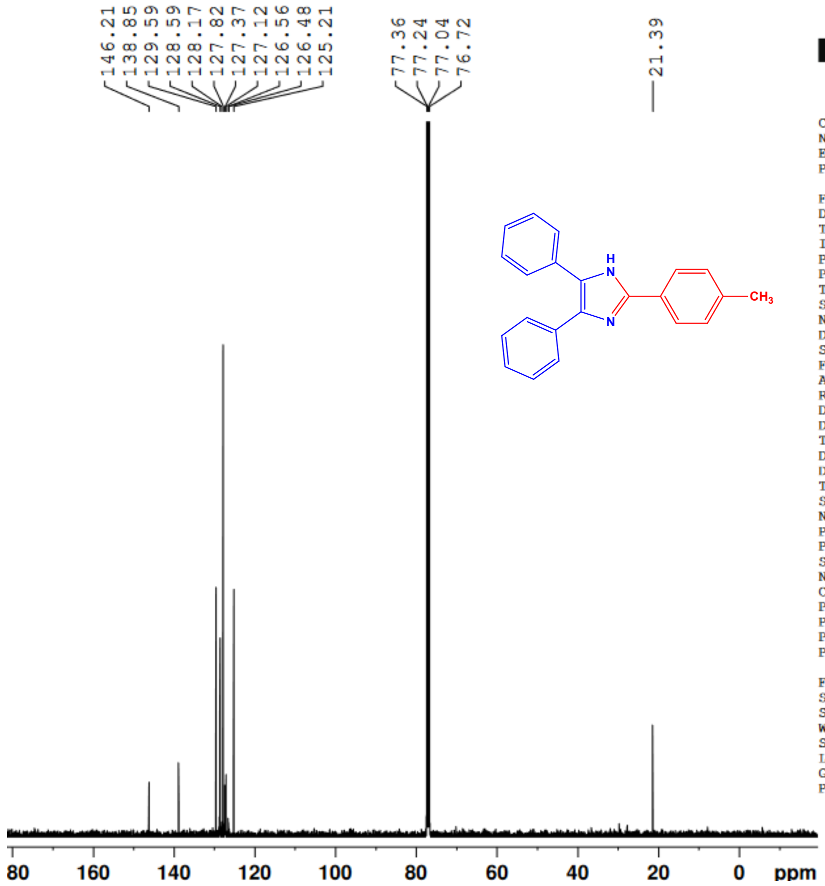

BRUKER

Current Data Parameters
NAME Dr.SYN280924
EXPNO 36
PROCNO 1

F2 - Acquisition Parameters
Date_ 20240929
Time 5.33 h
INSTRUM spect
PROBHD Z108618_0505 (
PULPROG zgpg30
TD 65536
SOLVENT CDCl3
NS 512
DS 4
SWH 24038.461 Hz
FIDRES 0.733596 Hz
AQ 1.3631488 sec
RG 175.97
DW 20.800 usec
DE 6.50 usec
TE 302.8 K
D1 2.00000000 sec
D11 0.03000000 sec
TD0 1
SFO1 100.6550186 MHz
NUC1 13C
P1 10.00 usec
PLW1 56.49300003 W
SFO2 400.2596010 MHz
NUC2 1H
CPDPRG[2 waltz16
PCPD2 90.00 usec
PLW2 15.21399975 W
PLW12 0.42261001 W
PLW13 0.21257000 W

F2 - Processing parameters
SI 32768
SF 100.6449542 MHz
WDW EM
SSB 0
LB 1.00 Hz
GB 0
PC 1.40

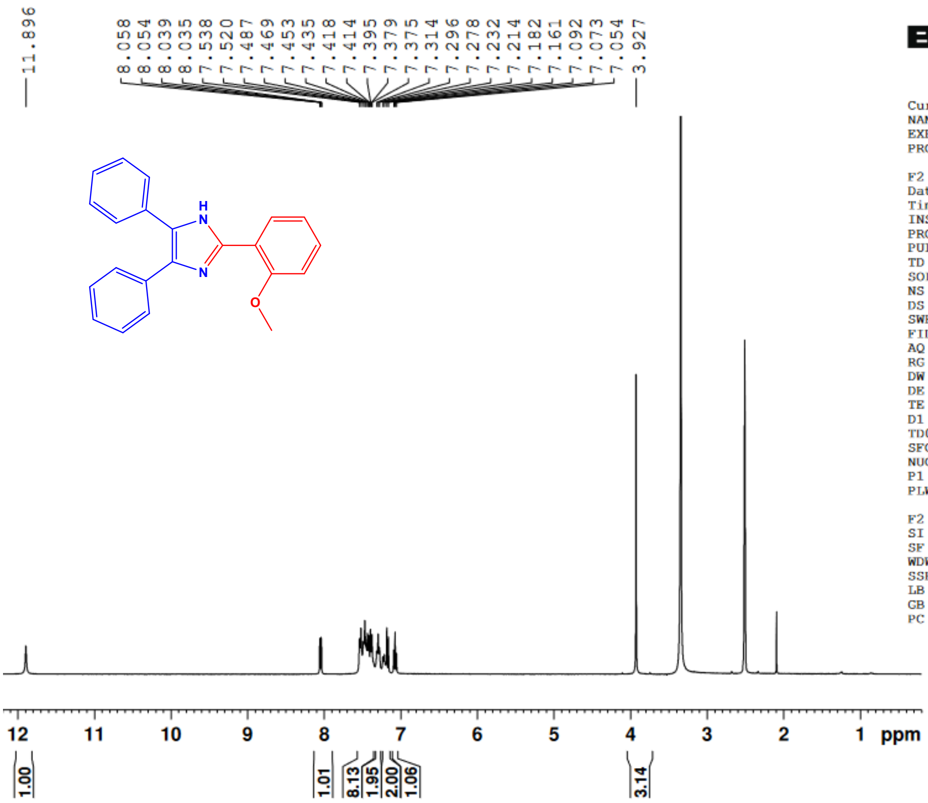

BRUKER

Current Data Parameters
NAME Dr.SYN140524
EXPNO 20
PROCNO 1

F2 - Acquisition Parameters
Date_ 20240514
Time 13.23 h
INSTRUM spect
PROBHD Z108618_0505 (
PULPROG zg30
TD 65536
SOLVENT DMSO
NS 64
DS 2
SWH 8012.820 Hz
FIDRES 0.244532 Hz
AQ 4.0894465 sec
RG 156.91
DW 62.400 usec
DE 6.50 usec
TE 303.7 K
D1 1.00000000 sec
TD0 1
SFO1 400.2604716 MHz
NUC1 1H
P1 15.00 usec
PLW1 15.21399975 W

F2 - Processing parameters
SI 65536
SF 400.2580000 MHz
WDW EM
SSB 0
LB 0.30 Hz
GB 0
PC 1.00

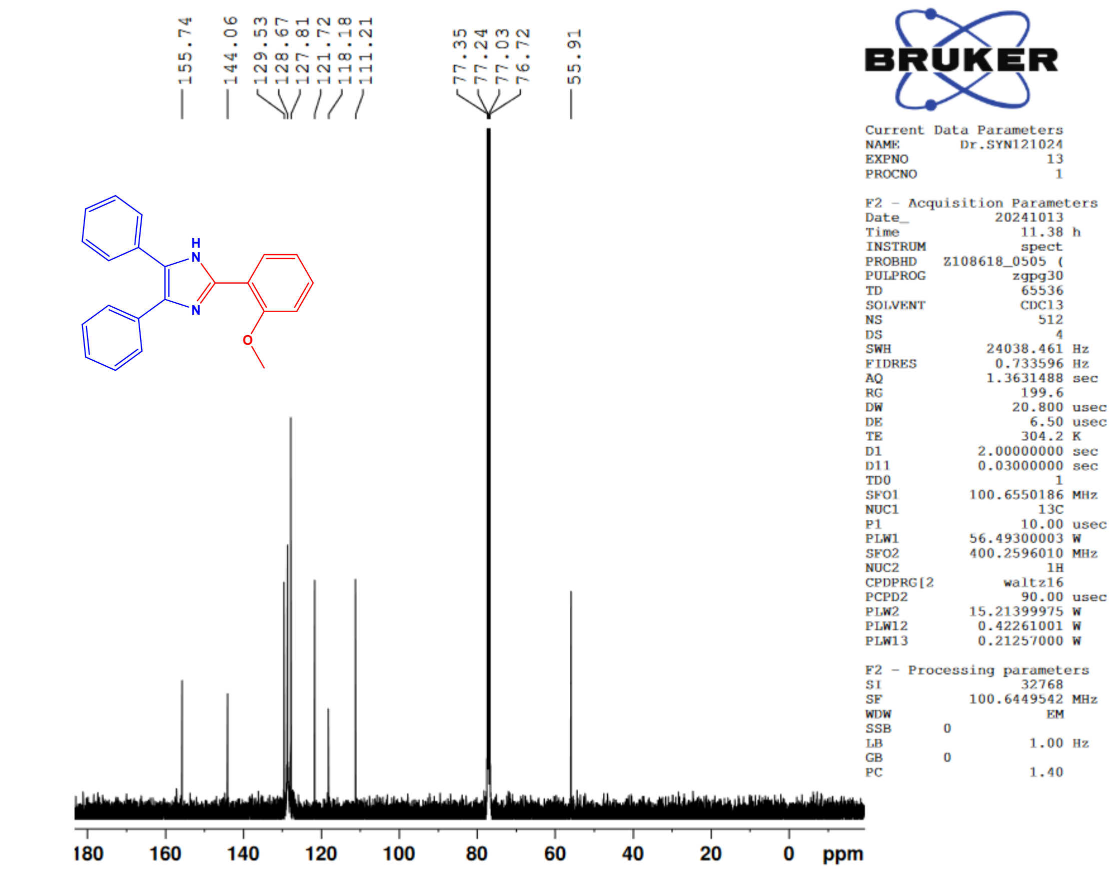
155.74
144.06
129.53
128.67
127.81
121.72
118.18
111.21
77.35
77.24
77.03
76.72
55.91
H
N
N
O
BRUKER
Current Data Parameters
NAME Dr.SYN121024
EXPNO 13
PROCNO 1
F2 - Acquisition Parameters
Date_ 20241013
Time 11.38 h
INSTRUM spect
PROBHD Z108618_0505 (
PULPROG zgpg30
TD 65536
SOLVENT CDCl3
NS 512
DS 4
SWH 24038.461 Hz
FIDRES 0.733596 Hz
AQ 1.3631488 sec
RG 199.6
DW 20.800 usec
DE 6.50 usec
TE 304.2 K
D1 2.00000000 sec
D11 0.03000000 sec
TD0 1
SFO1 100.6550186 MHz
NUC1 13C
P1 10.00 usec
PLW1 56.49300003 W
SFO2 400.2596010 MHz
NUC2 1H
CPDPRG[2 waltz16
PCPD2 90.00 usec
PLW2 15.21399975 W
PLW12 0.42261001 W
PLW13 0.21257000 W
F2 - Processing parameters
SI 32768
SF 100.6449542 MHz
WDW EM
SSB 0
LB 1.00 Hz
GB 0
PC 1.40
180 160 140 120 100 80 60 40 20 0 ppm

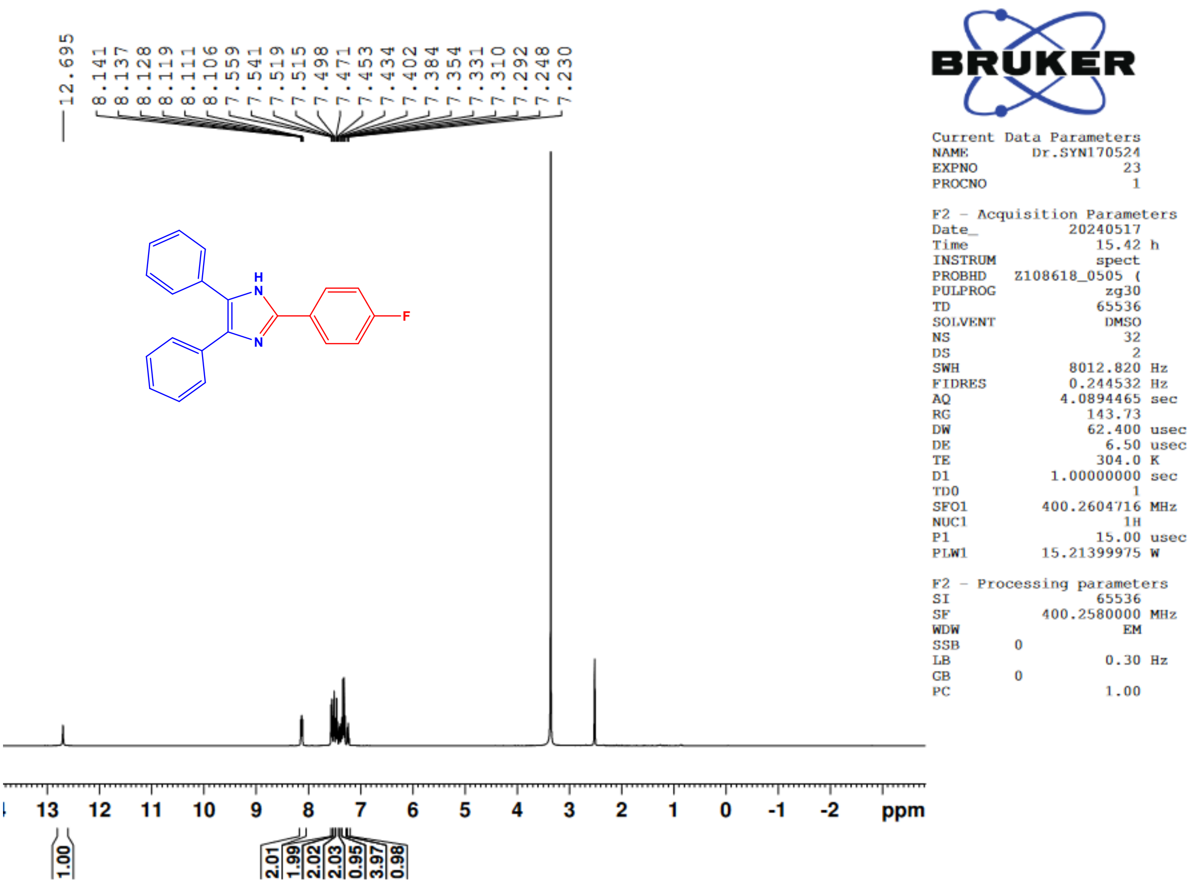
12.695
8.141
8.137
8.128
8.119
8.111
8.106
7.559
7.541
7.519
7.515
7.498
7.471
7.453
7.434
7.402
7.384
7.354
7.331
7.310
7.292
7.248
7.230
H
N
N
F
BRUKER
Current Data Parameters
NAME Dr.SYN170524
EXPNO 23
PROCNO 1
F2 - Acquisition Parameters
Date_ 20240517
Time 15.42 h
INSTRUM spect
PROBHD Z108618_0505 (
PULPROG zg30
TD 65536
SOLVENT DMSO
NS 32
DS 2
SWH 8012.820 Hz
FIDRES 0.244532 Hz
AQ 4.0894465 sec
RG 143.73
DW 62.400 usec
DE 6.50 usec
TE 304.0 K
D1 1.00000000 sec
TD0 1
SFO1 400.2604716 MHz
NUC1 1H
P1 15.00 usec
PLW1 15.21399975 W
F2 - Processing parameters
SI 65536
SF 400.2580000 MHz
WDW EM
SSB 0
LB 0.30 Hz
GB 0
PC 1.00
13 12 11 10 9 8 7 6 5 4 3 2 1 0 -1 -2 ppm
1.00
2.01
1.99
2.02
2.03
0.95
3.97
0.98

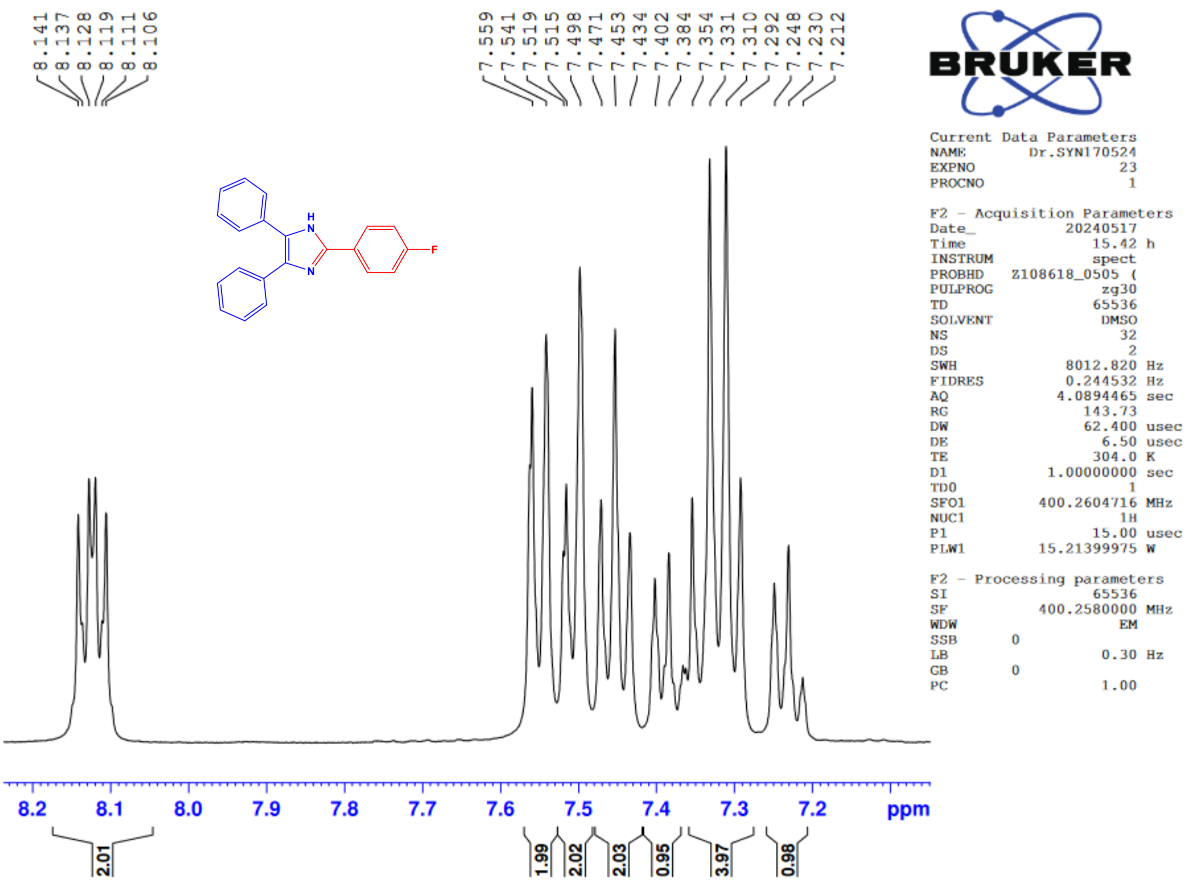
8.141
8.137
8.128
8.119
8.111
8.106
7.559
7.541
7.519
7.515
7.498
7.471
7.453
7.434
7.402
7.384
7.354
7.331
7.310
7.292
7.248
7.230
7.212
H
N
N
F
BRUKER
Current Data Parameters
NAME Dr.SYN170524
EXPNO 23
PROCNO 1
F2 - Acquisition Parameters
Date_ 20240517
Time 15.42 h
INSTRUM spect
PROBHD Z108618_0505 (
PULPROG zg30
TD 65536
SOLVENT DMSO
NS 32
DS 2
SWH 8012.820 Hz
FIDRES 0.244532 Hz
AQ 4.0894465 sec
RG 143.73
DW 62.400 usec
DE 6.50 usec
TE 304.0 K
D1 1.00000000 sec
TD0 1
SFO1 400.2604716 MHz
NUC1 1H
P1 15.00 usec
PLW1 15.21399975 W
F2 - Processing parameters
SI 65536
SF 400.2580000 MHz
WDW EM
SSB 0
LB 0.30 Hz
GB 0
PC 1.00
8.2
8.1
8.0
7.9
7.8
7.7
7.6
7.5
7.4
7.3
7.2
ppm
2.01
1.99
2.02
2.03
0.95
3.97
0.98

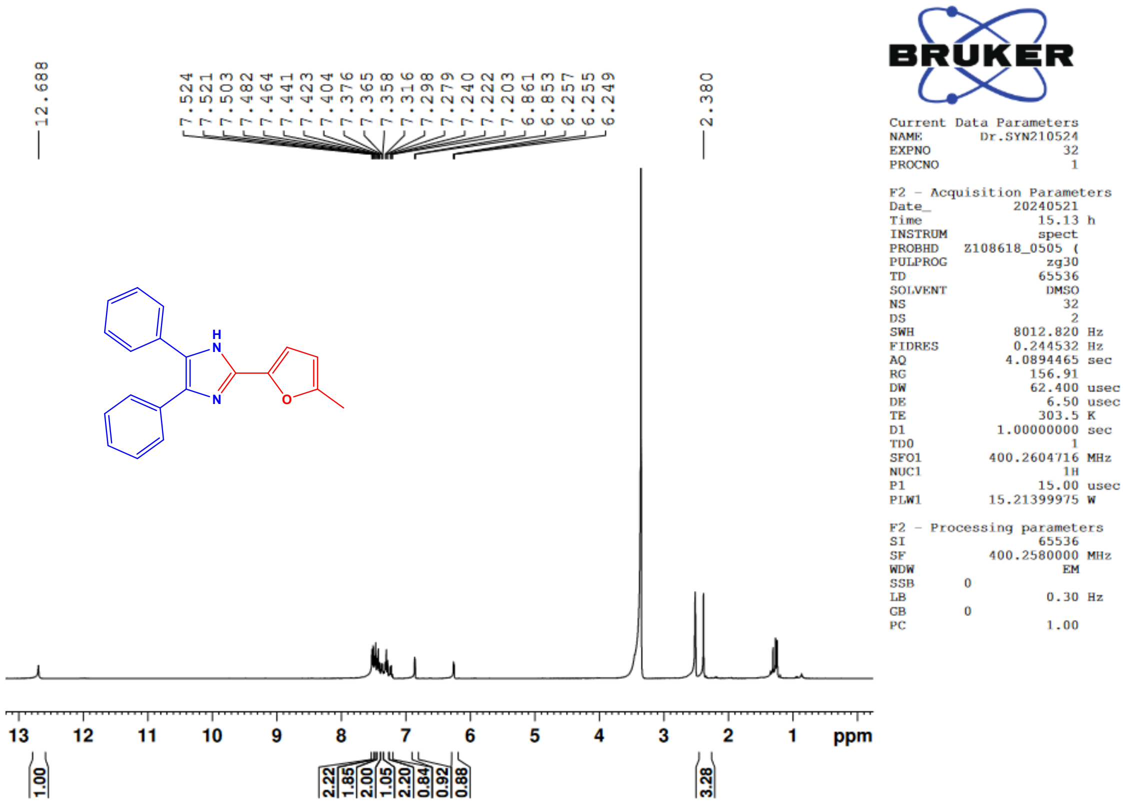
12.688
7.524
7.521
7.503
7.482
7.464
7.441
7.423
7.404
7.376
7.365
7.358
7.316
7.298
7.279
7.240
7.222
7.203
6.861
6.853
6.257
6.255
6.249
2.380
H
N
N
O
BRUKER
Current Data Parameters
NAME Dr.SYN210524
EXPNO 32
PROCNO 1
F2 - Acquisition Parameters
Date_ 20240521
Time 15.13 h
INSTRUM spect
PROBHD Z108618_0505 (
PULPROG zg30
TD 65536
SOLVENT DMSO
NS 32
DS 2
SWH 8012.820 Hz
FIDRES 0.244532 Hz
AQ 4.0894465 sec
RG 156.91
DW 62.400 usec
DE 6.50 usec
TE 303.5 K
D1 1.00000000 sec
TD0 1
SFO1 400.2604716 MHz
NUC1 1H
P1 15.00 usec
PLW1 15.21399975 W
F2 - Processing parameters
SI 65536
SF 400.2580000 MHz
WDW EM
SSB 0
LB 0.30 Hz
GB 0
PC 1.00
13
12
11
10
9
8
7
6
5
4
3
2
1
ppm
1.00
2.22
1.85
2.00
1.05
2.20
0.84
0.92
0.88
3.28

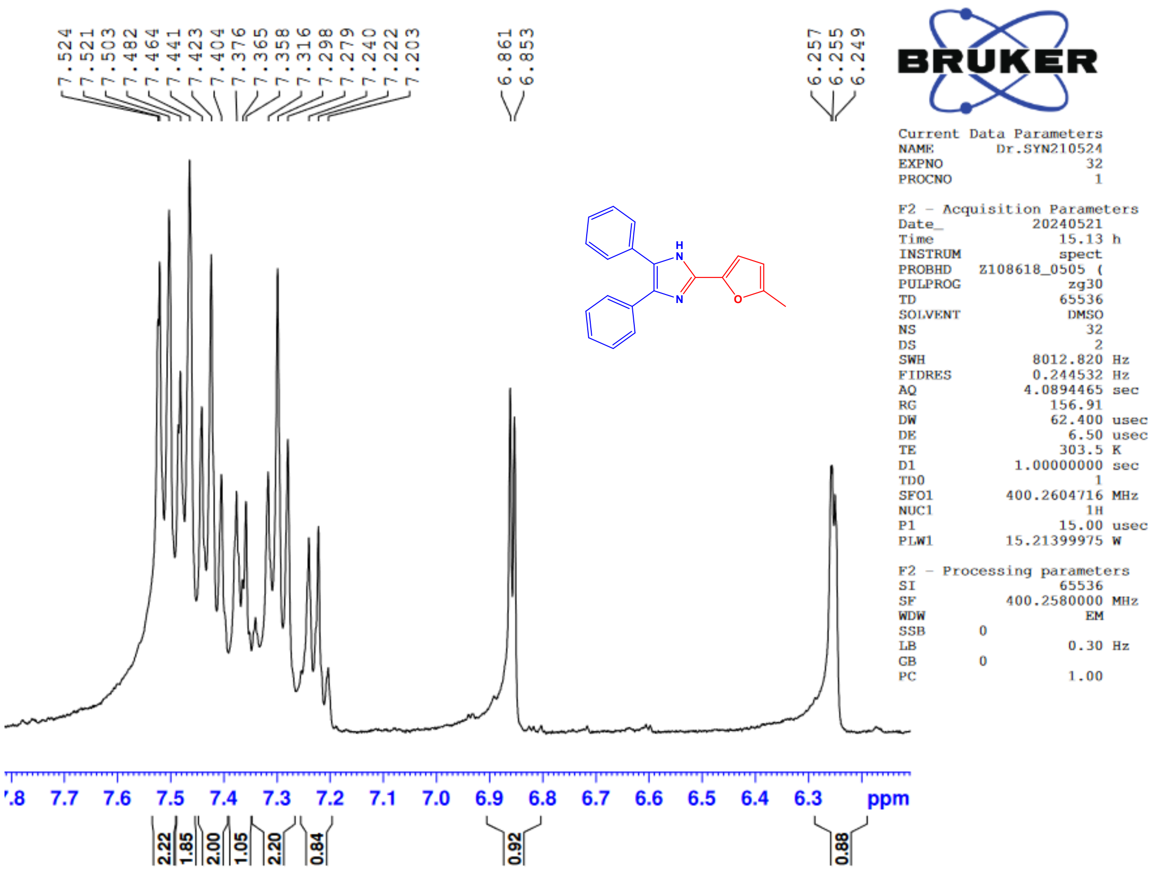
7.524
7.521
7.503
7.482
7.464
7.441
7.423
7.404
7.376
7.365
7.358
7.316
7.298
7.279
7.240
7.222
7.203
6.861
6.853
6.257
6.255
6.249
BRUKER
Current Data Parameters
NAME Dr.SYN210524
EXPNO 32
PROCNO 1
F2 - Acquisition Parameters
Date_ 20240521
Time 15.13 h
INSTRUM spect
PROBHD Z108618_0505 (
PULPROG zg30
TD 65536
SOLVENT DMSO
NS 32
DS 2
SWH 8012.820 Hz
FIDRES 0.244532 Hz
AQ 4.0894465 sec
RG 156.91
DW 62.400 usec
DE 6.50 usec
TE 303.5 K
D1 1.00000000 sec
TD0 1
SFO1 400.2604716 MHz
NUC1 1H
P1 15.00 usec
PLW1 15.21399975 W
F2 - Processing parameters
SI 65536
SF 400.2580000 MHz
WDW EM
SSB 0
LB 0.30 Hz
GB 0
PC 1.00
H
N
O
7.8 7.7 7.6 7.5 7.4 7.3 7.2 7.1 7.0 6.9 6.8 6.7 6.6 6.5 6.4 6.3 ppm
2.22
1.85
2.00
1.05
2.20
0.84
0.92
0.88

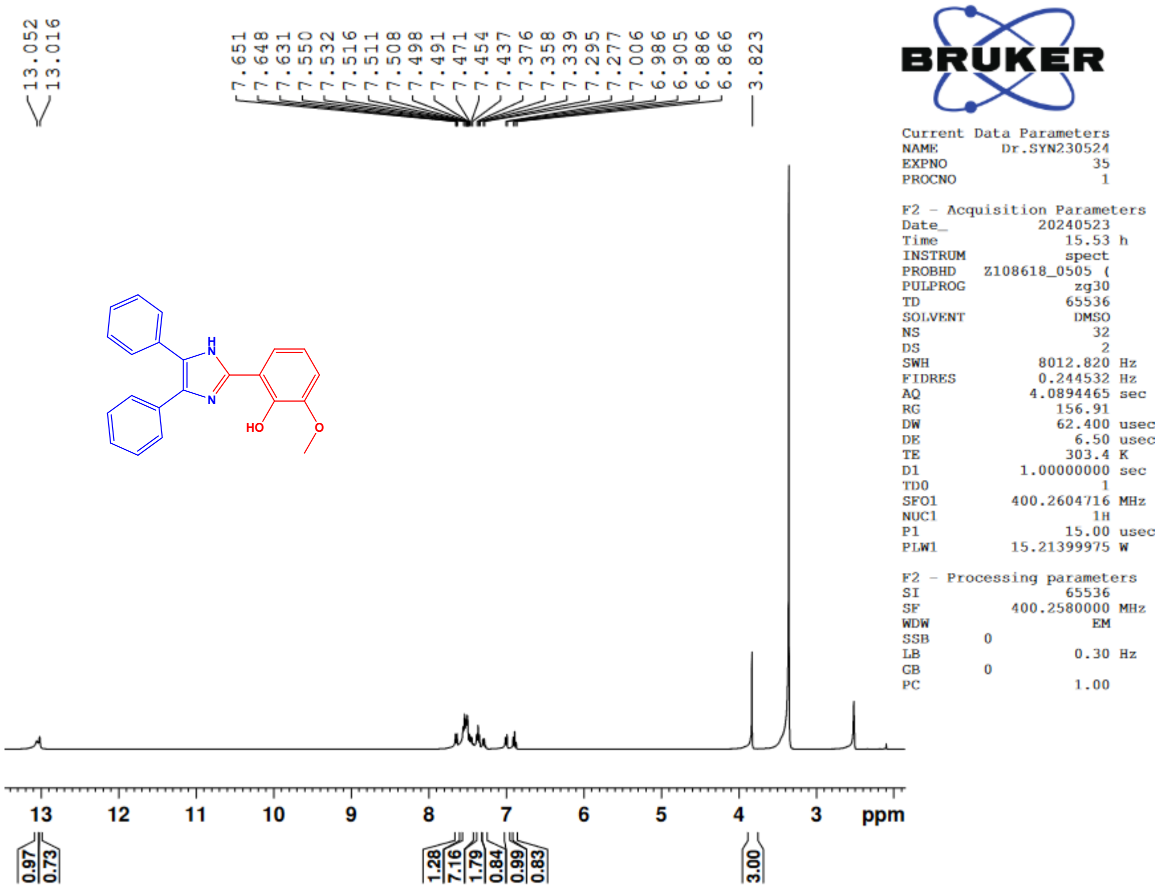
13.052
13.016
7.651
7.648
7.631
7.550
7.532
7.516
7.511
7.508
7.498
7.491
7.471
7.454
7.437
7.376
7.358
7.339
7.295
7.277
7.006
6.986
6.905
6.886
6.866
3.823
BRUKER
Current Data Parameters
NAME Dr.SYN230524
EXPNO 35
PROCNO 1
F2 - Acquisition Parameters
Date_ 20240523
Time 15.53 h
INSTRUM spect
PROBHD Z108618_0505 (
PULPROG zg30
TD 65536
SOLVENT DMSO
NS 32
DS 2
SWH 8012.820 Hz
FIDRES 0.244532 Hz
AQ 4.0894465 sec
RG 156.91
DW 62.400 usec
DE 6.50 usec
TE 303.4 K
D1 1.00000000 sec
TD0 1
SFO1 400.2604716 MHz
NUC1 1H
P1 15.00 usec
PLW1 15.21399975 W
F2 - Processing parameters
SI 65536
SF 400.2580000 MHz
WDW EM
SSB 0
LB 0.30 Hz
GB 0
PC 1.00
H
N
HO
O
13 12 11 10 9 8 7 6 5 4 3 ppm
0.97
0.73
1.28
7.16
1.79
0.84
0.99
0.83
3.00

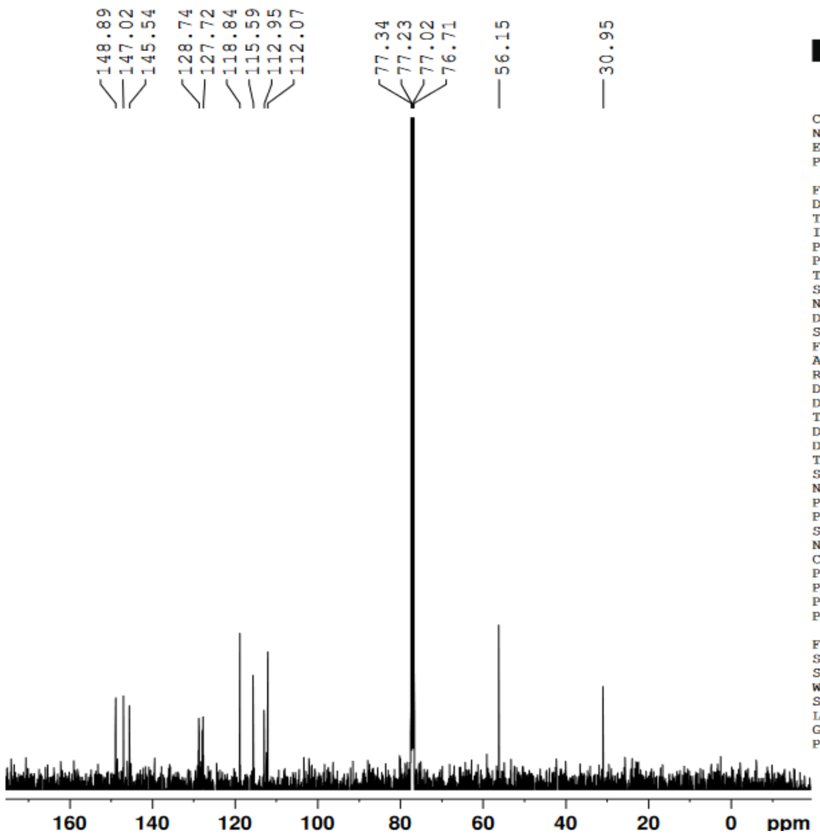

Current Data Parameters
NAME Dr.SYN280924
EXPNO 38
PROCNO 1

F2 - Acquisition Parameters
Date_ 20240929
Time 11.43 h
INSTRUM spect
PROBHD Z108618_0505 (
PULPROG zgpg30
TD 65536
SOLVENT CDCl3
NS 512
DS 4
SWH 24038.461 Hz
FIDRES 0.733596 Hz
AQ 1.3631488 sec
RG 199.6
DW 20.800 usec
DE 6.50 usec
TE 303.9 K
D1 2.00000000 sec
D11 0.03000000 sec
TD0 1
SFO1 100.6550186 MHz
NUC1 13C
P1 10.00 usec
PLW1 56.49300003 W
SFO2 400.2596010 MHz
NUC2 1H
CPDPRG[2 waltz16
PCPD2 90.00 usec
PLW2 15.21399975 W
PLW12 0.42261001 W
PLW13 0.21257000 W

F2 - Processing parameters
SI 32768
SF 100.6449542 MHz
WDW EM
SSB 0
LB 1.00 Hz
GB 0
PC 1.40

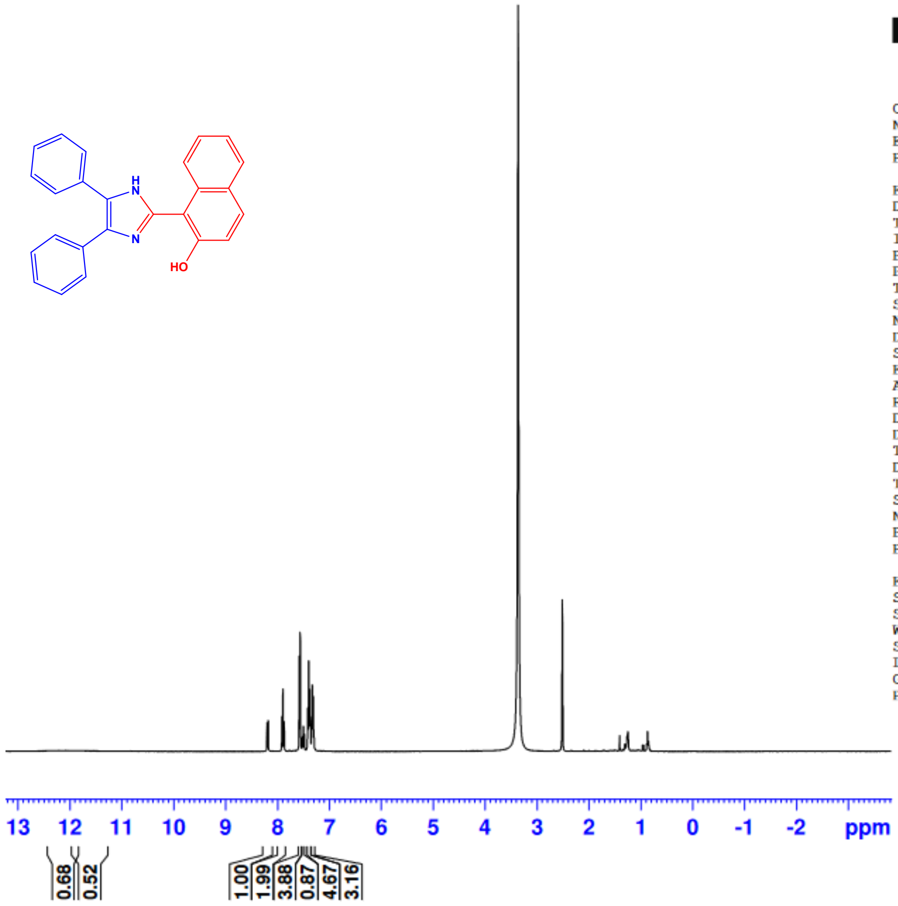

Current Data Parameters
NAME Dr.SYN170524 2
EXPNO 24
PROCNO 1

F2 - Acquisition Parameters
Date_ 20240517
Time 18.48 h
INSTRUM spect
PROBHD Z108618_0505 (
PULPROG zg30
TD 65536
SOLVENT DMSO
NS 32
DS 2
SWH 8012.820 Hz
FIDRES 0.244532 Hz
AQ 4.0894465 sec
RG 143.73
DW 62.400 usec
DE 6.50 usec
TE 303.5 K
D1 1.00000000 sec
TD0 1
SFO1 400.2604716 MHz
NUC1 1H
P1 15.00 usec
PLW1 15.21399975 W

F2 - Processing parameters
SI 65536
SF 400.2580000 MHz
WDW EM
SSB 0
LB 0.30 Hz
GB 0
PC 1.00

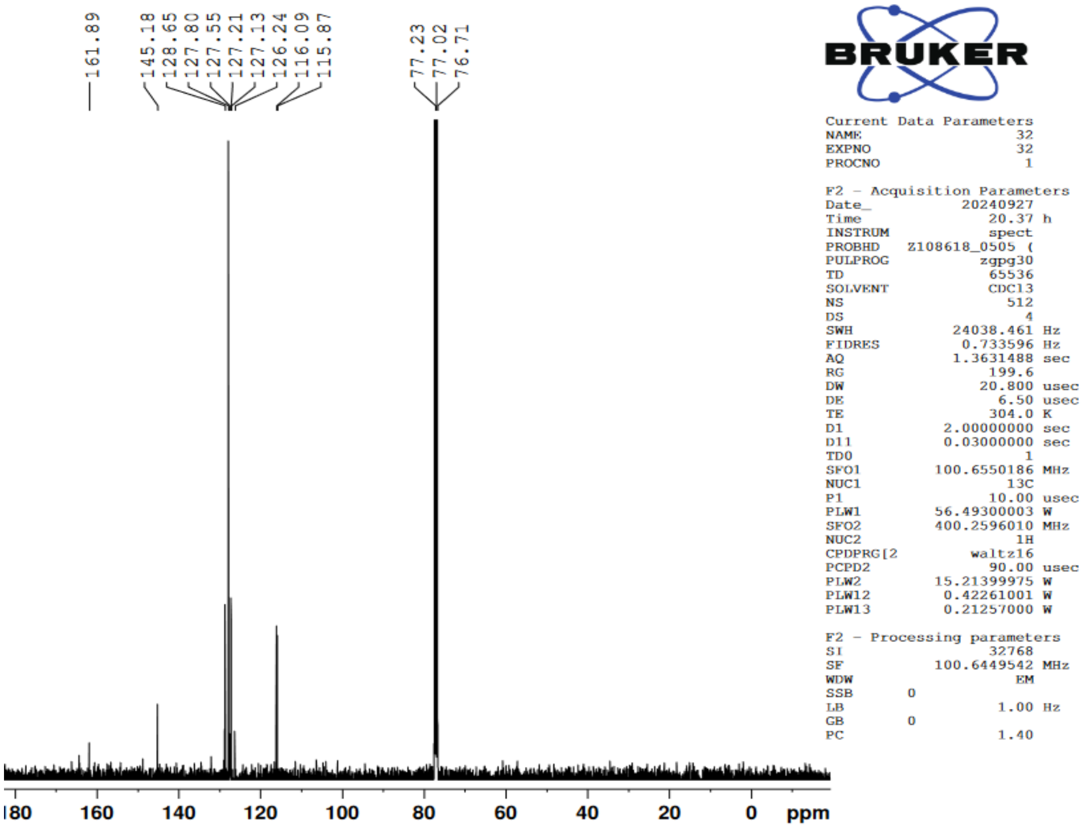
161.89
145.18
128.65
127.80
127.55
127.21
127.13
126.24
116.09
115.87
77.23
77.02
76.71
BRUKER
Current Data Parameters
NAME 32
EXPNO 32
PROCNO 1
F2 - Acquisition Parameters
Date_ 20240927
Time 20.37 h
INSTRUM spect
PROBHD Z108618_0505 (
PULPROG zgpg30
TD 65536
SOLVENT CDCl3
NS 512
DS 4
SWH 24038.461 Hz
FIDRES 0.733596 Hz
AQ 1.3631488 sec
RG 199.6
DW 20.800 usec
DE 6.50 usec
TE 304.0 K
D1 2.00000000 sec
D11 0.03000000 sec
TD0 1
SFO1 100.6550186 MHz
NUC1 13C
P1 10.00 usec
PLW1 56.49300003 W
SFO2 400.2596010 MHz
NUC2 1H
CPDPRG[2 waltz16
PCPD2 90.00 usec
PLW2 15.21399975 W
PLW12 0.42261001 W
PLW13 0.21257000 W
F2 - Processing parameters
SI 32768
SF 100.6449542 MHz
WDW EM
SSB 0
LB 1.00 Hz
GB 0
PC 1.40
180 160 140 120 100 80 60 40 20 0 ppm

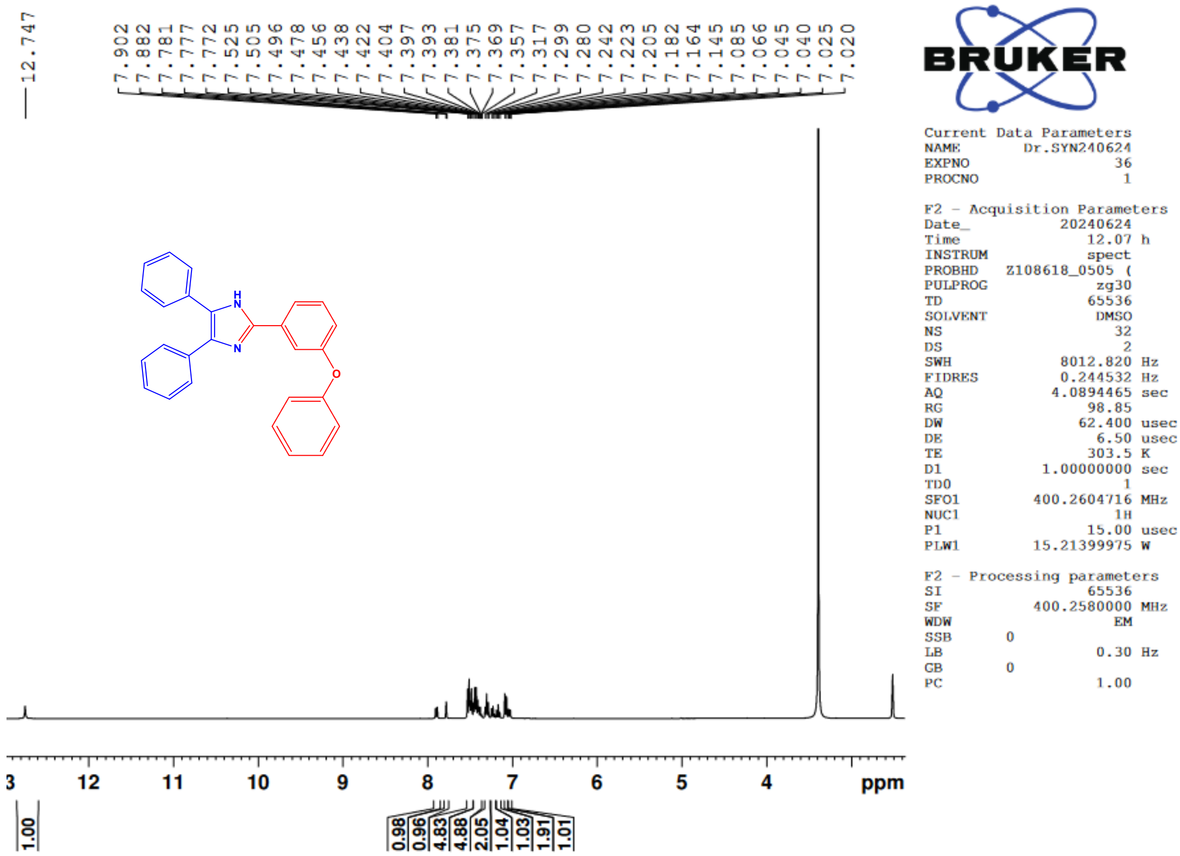
12.747
7.902
7.882
7.781
7.777
7.772
7.525
7.505
7.496
7.478
7.456
7.438
7.422
7.404
7.397
7.393
7.381
7.375
7.369
7.357
7.317
7.299
7.280
7.242
7.223
7.205
7.182
7.164
7.145
7.085
7.066
7.045
7.040
7.025
7.020
BRUKER
Current Data Parameters
NAME Dr.SYN240624
EXPNO 36
PROCNO 1
F2 - Acquisition Parameters
Date_ 20240624
Time 12.07 h
INSTRUM spect
PROBHD Z108618_0505 (
PULPROG zg30
TD 65536
SOLVENT DMSO
NS 32
DS 2
SWH 8012.820 Hz
FIDRES 0.244532 Hz
AQ 4.0894465 sec
RG 98.85
DW 62.400 usec
DE 6.50 usec
TE 303.5 K
D1 1.00000000 sec
TD0 1
SFO1 400.2604716 MHz
NUC1 1H
P1 15.00 usec
PLW1 15.21399975 W
F2 - Processing parameters
SI 65536
SF 400.2580000 MHz
WDW EM
SSB 0
LB 0.30 Hz
GB 0
PC 1.00
H
N
O
3 12 11 10 9 8 7 6 5 4 ppm
1.00
0.98
0.96
4.83
4.88
2.05
1.04
1.03
1.91
1.01

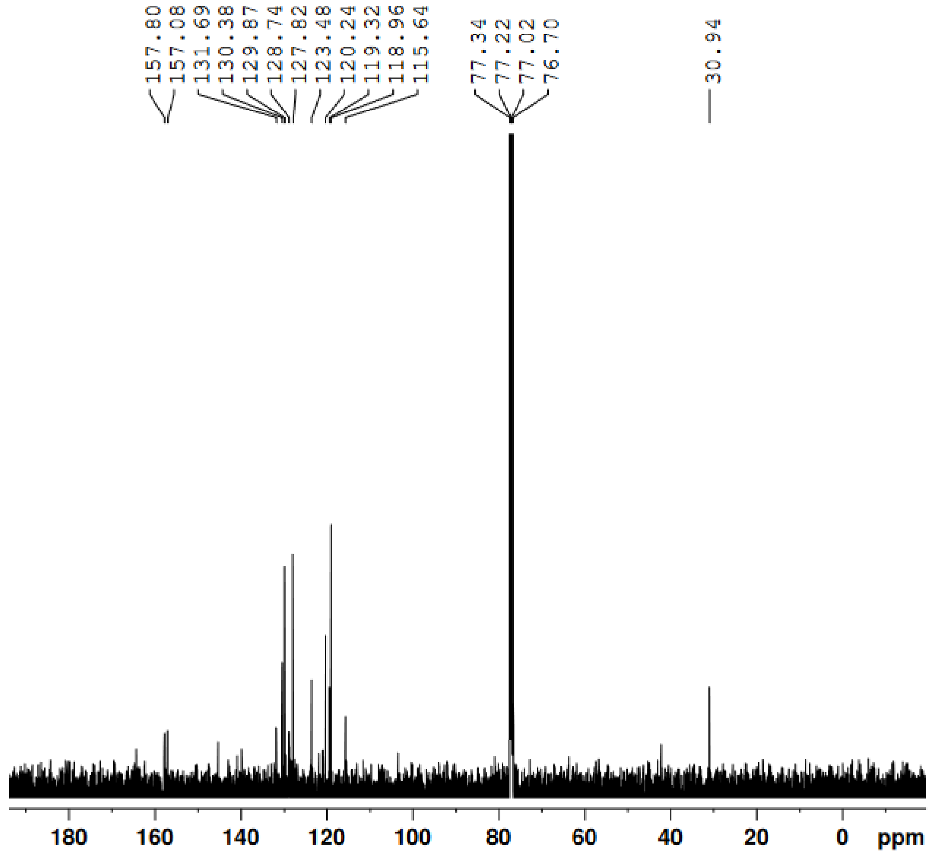

BRUKER

Current Data Parameters
NAME Dr.SYN191024
EXPNO 30
PROCNO 1

F2 - Acquisition Parameters
Date_ 20241019
Time 20.35 h
INSTRUM spect
PROBHD Z108618_0505 (
PULPROG zgpg30
TD 65536
SOLVENT CDCl3
NS 512
DS 4
SWH 24038.461 Hz
FIDRES 0.733596 Hz
AQ 1.3631488 sec
RG 156.91
DW 20.800 usec
DE 6.50 usec
TE 304.6 K
D1 2.00000000 sec
D11 0.03000000 sec
TD0 1
SFO1 100.6550186 MHz
NUC1 13C
P1 10.00 usec
PLW1 56.49300003 W
SFO2 400.2596010 MHz
NUC2 1H
CPDPRG[2 waltz16
PCPD2 90.00 usec
PLW2 15.21399975 W
PLW12 0.42261001 W
PLW13 0.21257000 W

F2 - Processing parameters
SI 32768
SF 100.6449542 MHz
WDW EM
SSB 0
LB 1.00 Hz
GB 0
PC 1.40

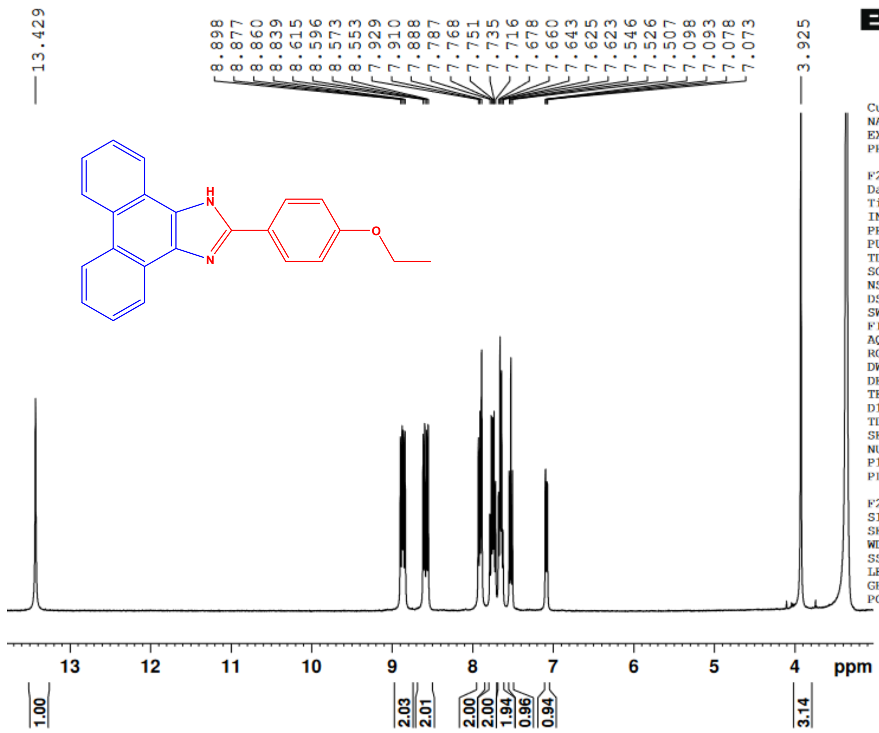

BRUKER

Current Data Parameters
NAME Dr.SYN270524
EXPNO 41
PROCNO 1

F2 - Acquisition Parameters
Date_ 20240527
Time 12.55 h
INSTRUM spect
PROBHD Z108618_0505 (
PULPROG zg30
TD 65536
SOLVENT DMSO
NS 32
DS 2
SWH 8012.820 Hz
FIDRES 0.244532 Hz
AQ 4.0894465 sec
RG 143.73
DW 62.400 usec
DE 6.50 usec
TE 304.0 K
D1 1.00000000 sec
TD0 1
SFO1 400.2604716 MHz
NUC1 1H
P1 15.00 usec
PLW1 15.21399975 W

F2 - Processing parameters
SI 65536
SF 400.2580000 MHz
WDW EM
SSB 0
LB 0.30 Hz
GB 0
PC 1.00

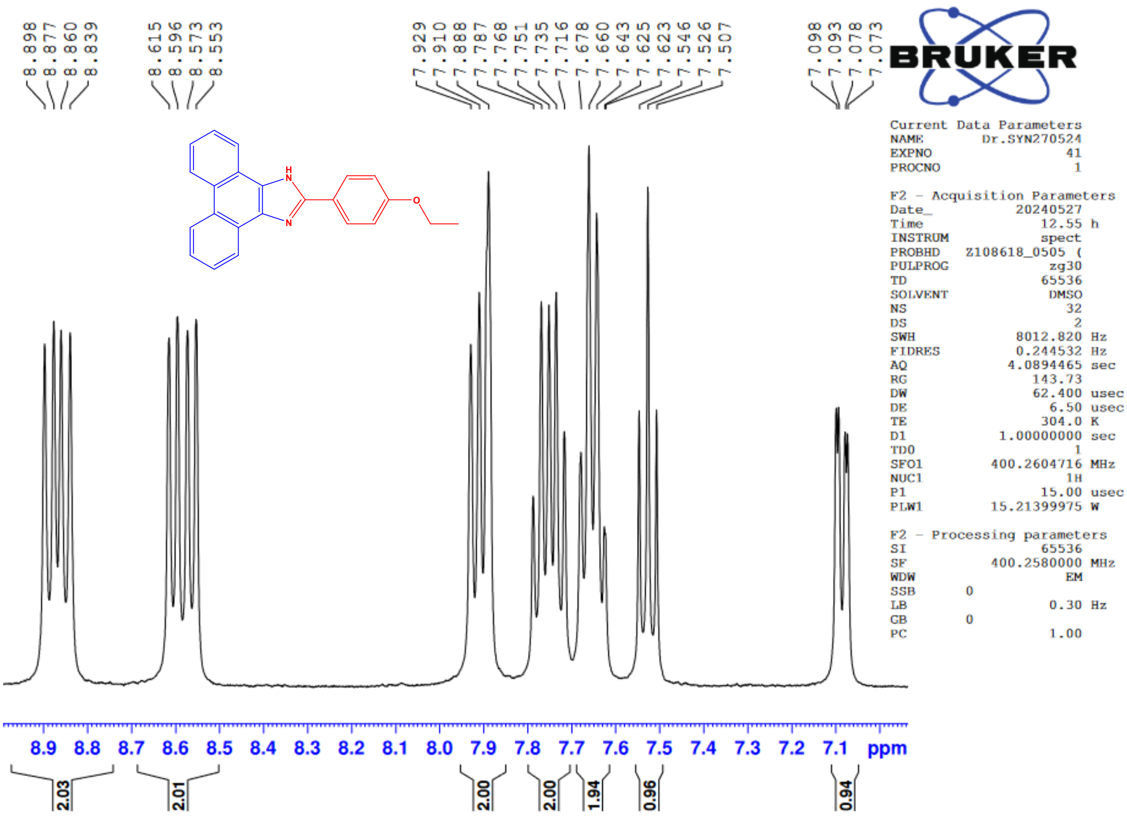
8.898
8.877
8.860
8.839
8.615
8.596
8.573
8.553
7.929
7.910
7.888
7.887
7.768
7.751
7.735
7.716
7.678
7.660
7.643
7.625
7.623
7.546
7.526
7.507
7.098
7.093
7.078
7.073
BRUKER
Current Data Parameters
NAME Dr.SYN270524
EXPNO 41
PROCNO 1
F2 - Acquisition Parameters
Date_ 20240527
Time 12.55 h
INSTRUM spect
PROBHD Z108618_0505 (
PULPROG zg30
TD 65536
SOLVENT DMSO
NS 32
DS 2
SWH 8012.820 Hz
FIDRES 0.244532 Hz
AQ 4.0894465 sec
RG 143.73
DW 62.400 usec
DE 6.50 usec
TE 304.0 K
D1 1.00000000 sec
TD0 1
SFO1 400.2604716 MHz
NUC1 1H
P1 15.00 usec
PLW1 15.21399975 W
F2 - Processing parameters
SI 65536
SF 400.2580000 MHz
WDW EM
SSB 0
LB 0.30 Hz
GB 0
PC 1.00
8.9 8.8 8.7 8.6 8.5 8.4 8.3 8.2 8.1 8.0 7.9 7.8 7.7 7.6 7.5 7.4 7.3 7.2 7.1 ppm
2.03
2.01
2.00
2.00
1.94
0.96
0.94

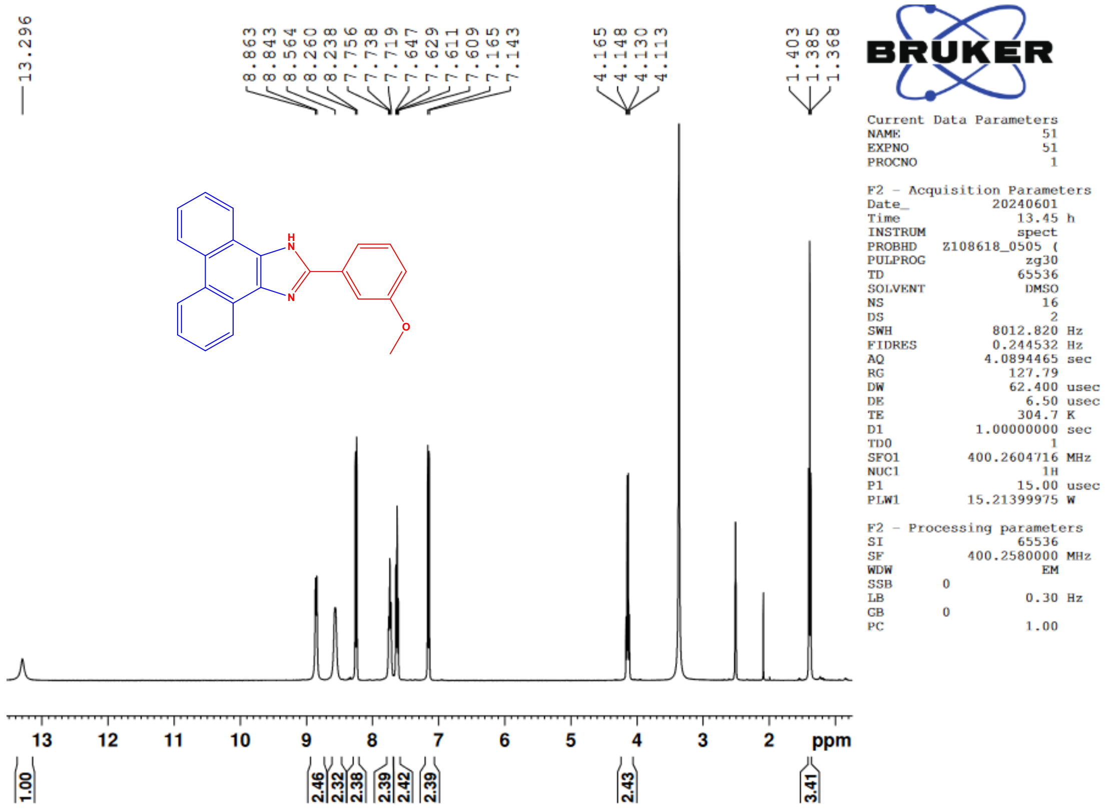
13.296
8.863
8.843
8.564
8.260
8.238
7.756
7.738
7.719
7.647
7.629
7.611
7.609
7.165
7.143
4.165
4.148
4.130
4.113
1.403
1.385
1.368
BRUKER
Current Data Parameters
NAME 51
EXPNO 51
PROCNO 1
F2 - Acquisition Parameters
Date_ 20240601
Time 13.45 h
INSTRUM spect
PROBHD Z108618_0505 (
PULPROG zg30
TD 65536
SOLVENT DMSO
NS 16
DS 2
SWH 8012.820 Hz
FIDRES 0.244532 Hz
AQ 4.0894465 sec
RG 127.79
DW 62.400 usec
DE 6.50 usec
TE 304.7 K
D1 1.00000000 sec
TD0 1
SFO1 400.2604716 MHz
NUC1 1H
P1 15.00 usec
PLW1 15.21399975 W
F2 - Processing parameters
SI 65536
SF 400.2580000 MHz
WDW EM
SSB 0
LB 0.30 Hz
GB 0
PC 1.00
13 12 11 10 9 8 7 6 5 4 3 2 ppm
1.00
2.46
2.32
2.38
2.39
2.42
2.39
2.43
3.41

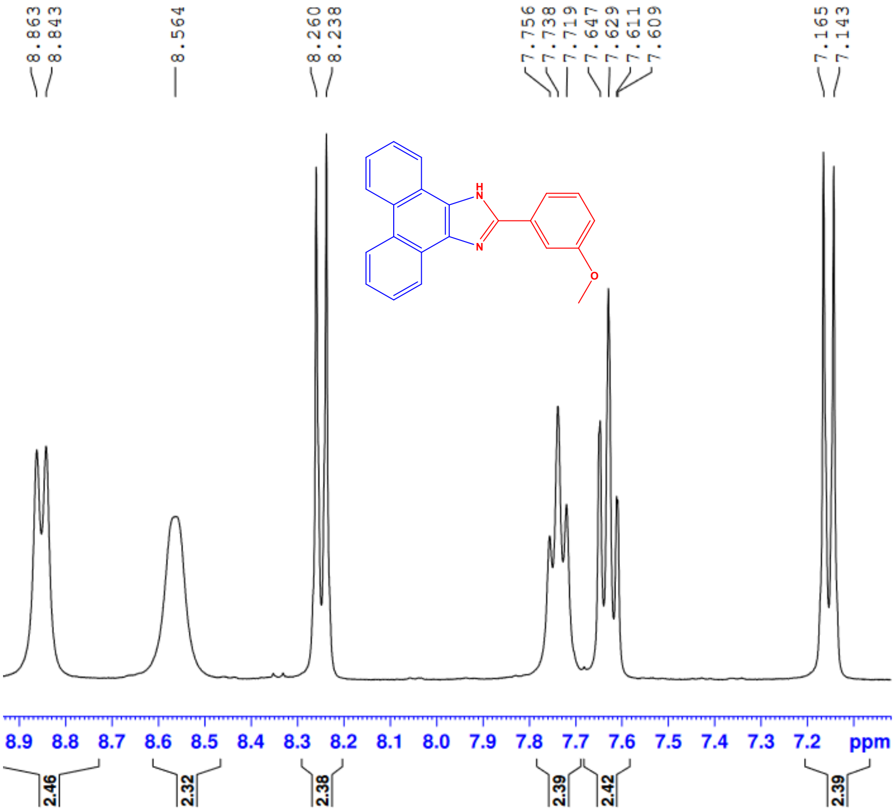

Current Data Parameters
NAME 51
EXPNO 51
PROCNO 1

F2 - Acquisition Parameters
Date_ 20240601
Time 13.45 h
INSTRUM spect
PROBHD Z108618_0505 (
PULPROG zg30
TD 65536
SOLVENT DMSO
NS 16
DS 2
SWH 8012.820 Hz
FIDRES 0.244532 Hz
AQ 4.0894465 sec
RG 127.79
DW 62.400 usec
DE 6.50 usec
TE 304.7 K
D1 1.00000000 sec
TD0 1
SFO1 400.2604716 MHz
NUC1 1H
P1 15.00 usec
PLW1 15.21399975 W

F2 - Processing parameters
SI 65536
SF 400.2580000 MHz
WDW EM
SSB 0
LB 0.30 Hz
GB 0
PC 1.00

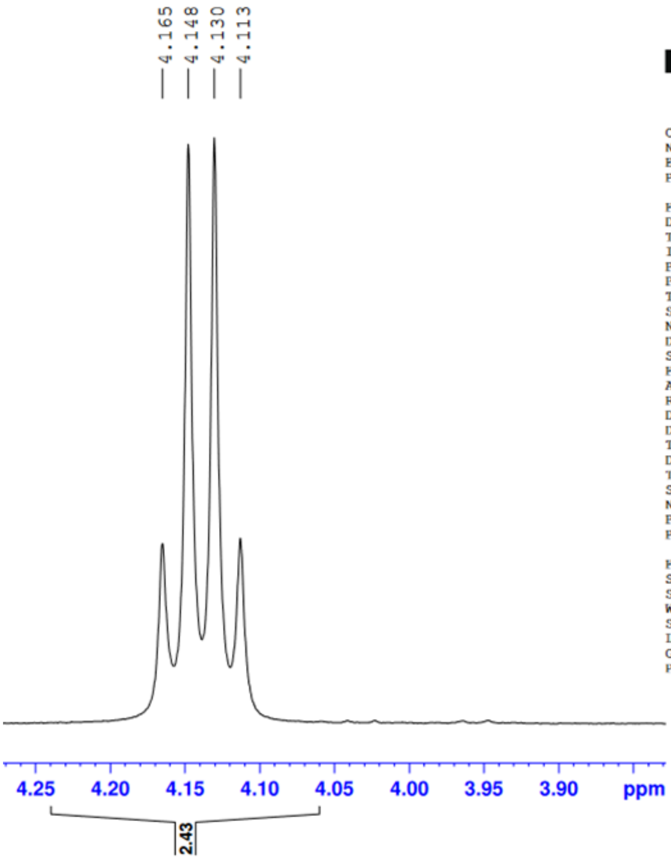

Current Data Parameters
NAME 51
EXPNO 51
PROCNO 1

F2 - Acquisition Parameters
Date_ 20240601
Time 13.45 h
INSTRUM spect
PROBHD Z108618_0505 (
PULPROG zg30
TD 65536
SOLVENT DMSO
NS 16
DS 2
SWH 8012.820 Hz
FIDRES 0.244532 Hz
AQ 4.0894465 sec
RG 127.79
DW 62.400 usec
DE 6.50 usec
TE 304.7 K
D1 1.00000000 sec
TD0 1
SFO1 400.2604716 MHz
NUC1 1H
P1 15.00 usec
PLW1 15.21399975 W

F2 - Processing parameters
SI 65536
SF 400.2580000 MHz
WDW EM
SSB 0
LB 0.30 Hz
GB 0
PC 1.00

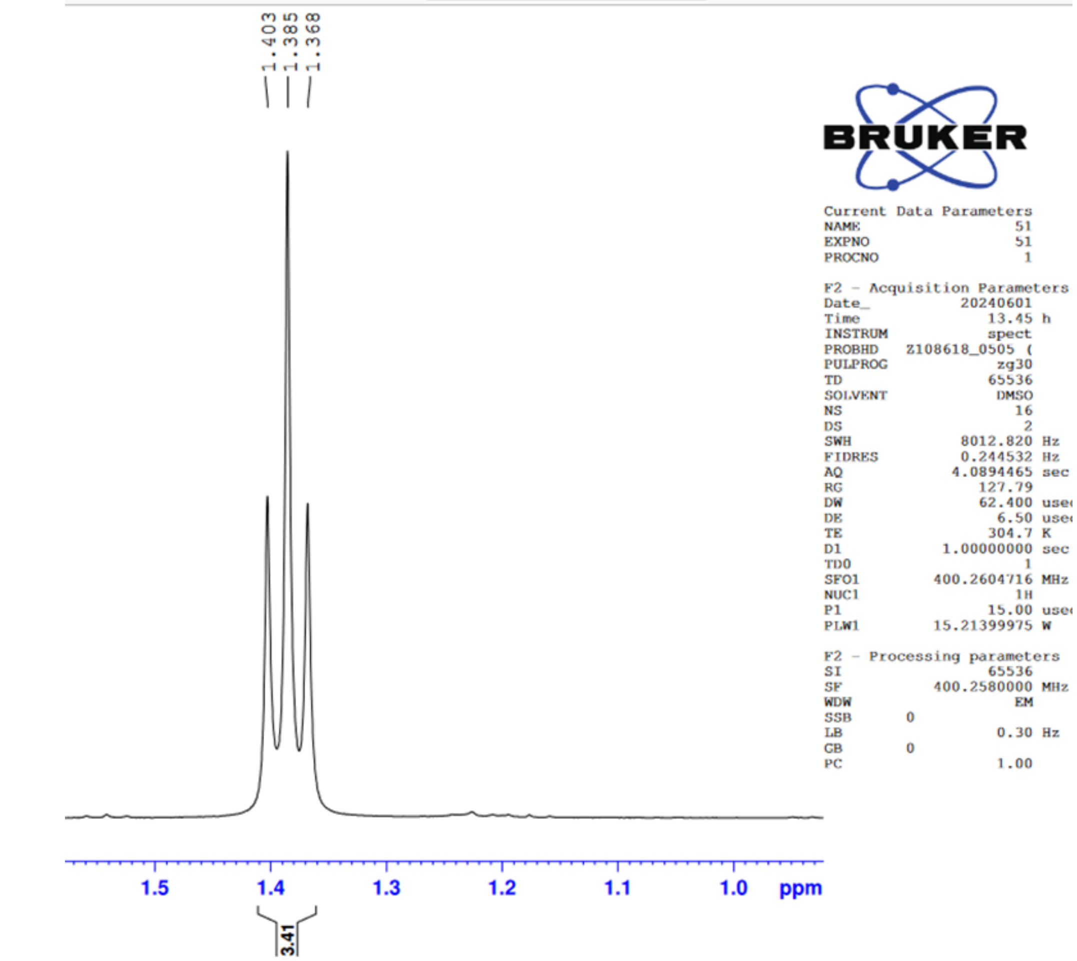
1.403
1.385
1.368
BRUKER
Current Data Parameters
NAME 51
EXPNO 51
PROCNO 1
F2 - Acquisition Parameters
Date_ 20240601
Time 13.45 h
INSTRUM spect
PROBHD Z108618_0505 (
PULPROG zg30
TD 65536
SOLVENT DMSO
NS 16
DS 2
SWH 8012.820 Hz
FIDRES 0.244532 Hz
AQ 4.0894465 sec
RG 127.79
DW 62.400 usec
DE 6.50 usec
TE 304.7 K
D1 1.00000000 sec
TD0 1
SFO1 400.2604716 MHz
NUC1 1H
P1 15.00 usec
PLW1 15.21399975 W
F2 - Processing parameters
SI 65536
SF 400.2580000 MHz
WDW EM
SSB 0
LB 0.30 Hz
GB 0
PC 1.00
1.5
1.4
1.3
1.2
1.1
1.0
ppm
3.41

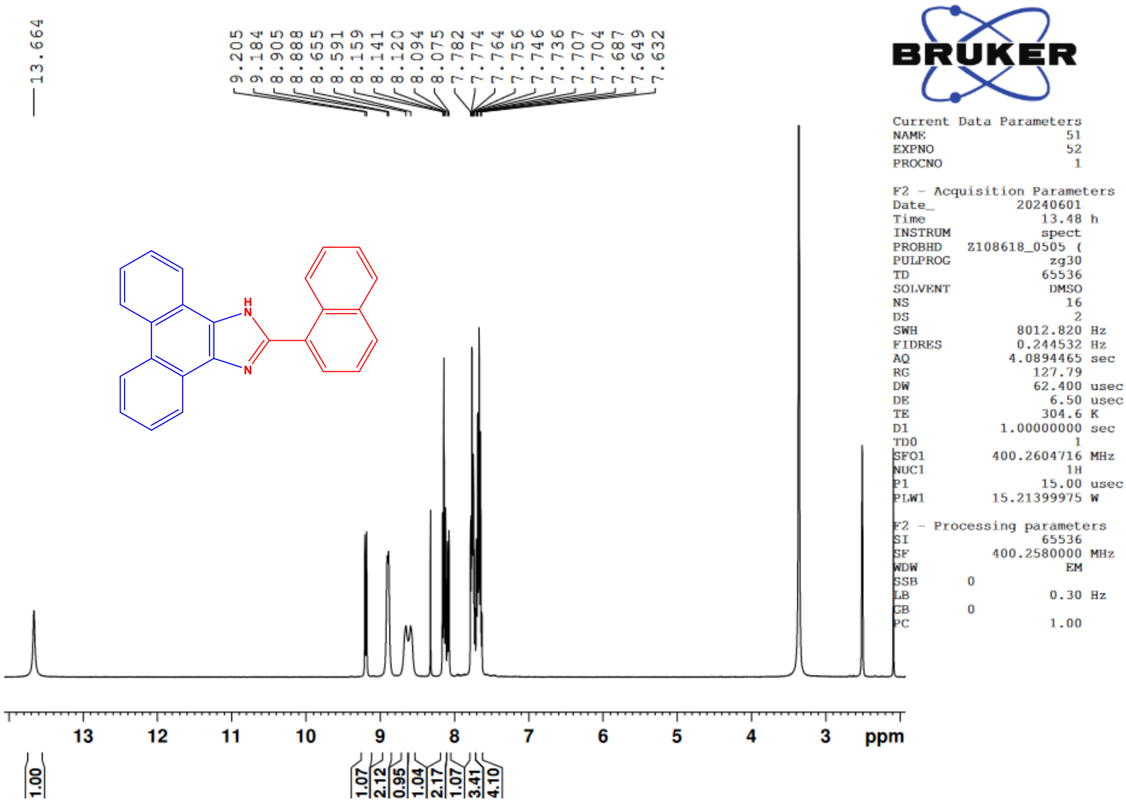
13.664
9.205
9.184
8.905
8.888
8.655
8.591
8.159
8.141
8.120
8.094
8.075
7.782
7.774
7.764
7.746
7.736
7.707
7.704
7.687
7.649
7.632
H
N
N
BRUKER
Current Data Parameters
NAME 51
EXPNO 52
PROCNO 1
F2 - Acquisition Parameters
Date_ 20240601
Time 13.48 h
INSTRUM spect
PROBHD Z108618_0505 (
PULPROG zg30
TD 65536
SOLVENT DMSO
NS 16
DS 2
SWH 8012.820 Hz
FIDRES 0.244532 Hz
AQ 4.0894465 sec
RG 127.79
DW 62.400 usec
DE 6.50 usec
TE 304.6 K
D1 1.00000000 sec
TD0 1
SFO1 400.2604716 MHz
NUC1 1H
P1 15.00 usec
PLW1 15.21399975 W
F2 - Processing parameters
SI 65536
SF 400.2580000 MHz
WDW EM
SSB 0
LB 0.30 Hz
GB 0
PC 1.00
13
12
11
10
9
8
7
6
5
4
3
ppm
1.00
1.07
2.12
0.95
1.04
2.17
1.07
3.41
4.10

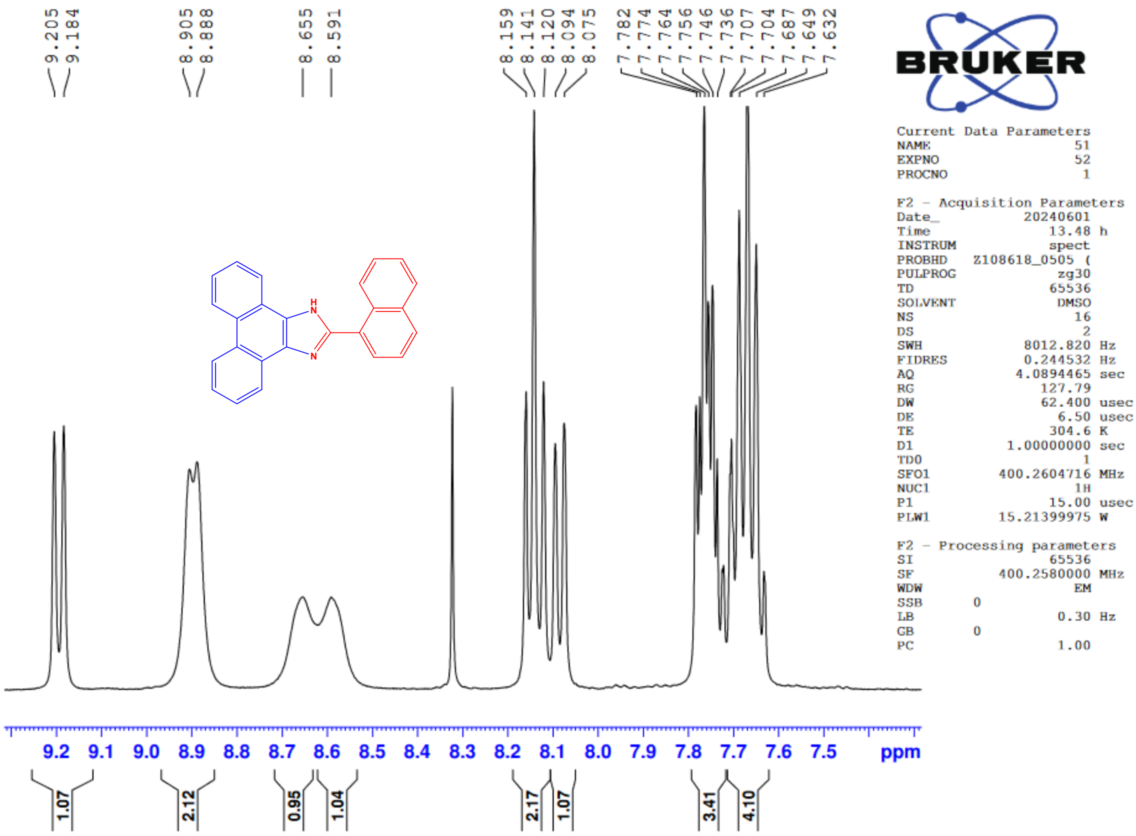
9.205
9.184
8.905
8.888
8.655
8.591
8.159
8.141
8.120
8.094
8.075
7.782
7.774
7.764
7.756
7.746
7.736
7.707
7.704
7.687
7.649
7.632
H
N
9.2 9.1 9.0 8.9 8.8 8.7 8.6 8.5 8.4 8.3 8.2 8.1 8.0 7.9 7.8 7.7 7.6 7.5 ppm
1.07
2.12
0.95
1.04
2.17
1.07
3.41
4.10
Current Data Parameters
NAME 51
EXPNO 52
PROCNO 1
F2 - Acquisition Parameters
Date_ 20240601
Time 13.48 h
INSTRUM spect
PROBHD Z108618_0505 (
PULPROG zg30
TD 65536
SOLVENT DMSO
NS 16
DS 2
SWH 8012.820 Hz
FIDRES 0.244532 Hz
AQ 4.0894465 sec
RG 127.79
DW 62.400 usec
DE 6.50 usec
TE 304.6 K
D1 1.00000000 sec
TD0 1
SFO1 400.2604716 MHz
NUC1 1H
P1 15.00 usec
PLW1 15.21399975 W
F2 - Processing parameters
SI 65536
SF 400.2580000 MHz
WDW EM
SSB 0
LB 0.30 Hz
GB 0
PC 1.00

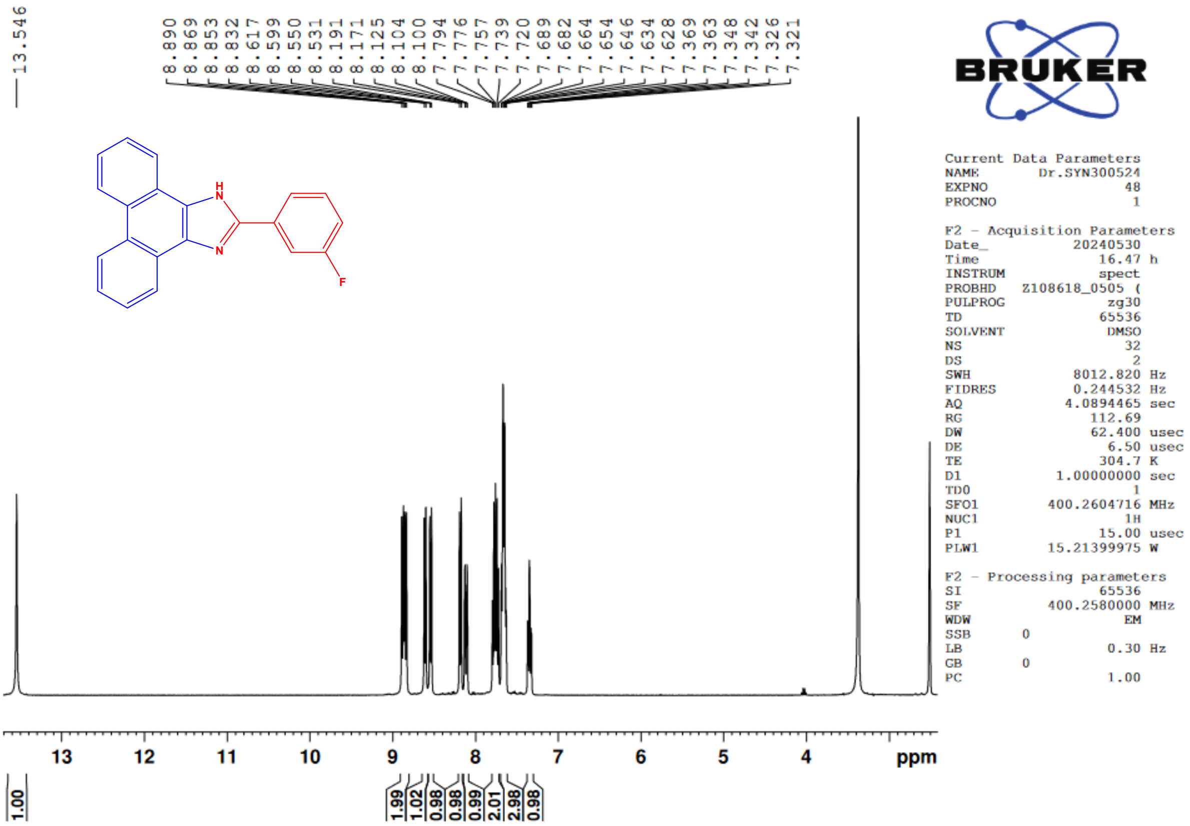
13.546
8.890
8.869
8.853
8.832
8.617
8.599
8.550
8.531
8.191
8.171
8.125
8.104
8.100
7.794
7.776
7.757
7.739
7.720
7.689
7.682
7.664
7.654
7.646
7.634
7.628
7.369
7.363
7.348
7.342
7.326
7.321
H
N
F
13 12 11 10 9 8 7 6 5 4 ppm
1.00
1.99
1.02
0.98
0.98
0.99
2.01
2.98
0.98
Current Data Parameters
NAME Dr.SYN300524
EXPNO 48
PROCNO 1
F2 - Acquisition Parameters
Date_ 20240530
Time 16.47 h
INSTRUM spect
PROBHD Z108618_0505 (
PULPROG zg30
TD 65536
SOLVENT DMSO
NS 32
DS 2
SWH 8012.820 Hz
FIDRES 0.244532 Hz
AQ 4.0894465 sec
RG 112.69
DW 62.400 usec
DE 6.50 usec
TE 304.7 K
D1 1.00000000 sec
TD0 1
SFO1 400.2604716 MHz
NUC1 1H
P1 15.00 usec
PLW1 15.21399975 W
F2 - Processing parameters
SI 65536
SF 400.2580000 MHz
WDW EM
SSB 0
LB 0.30 Hz
GB 0
PC 1.00

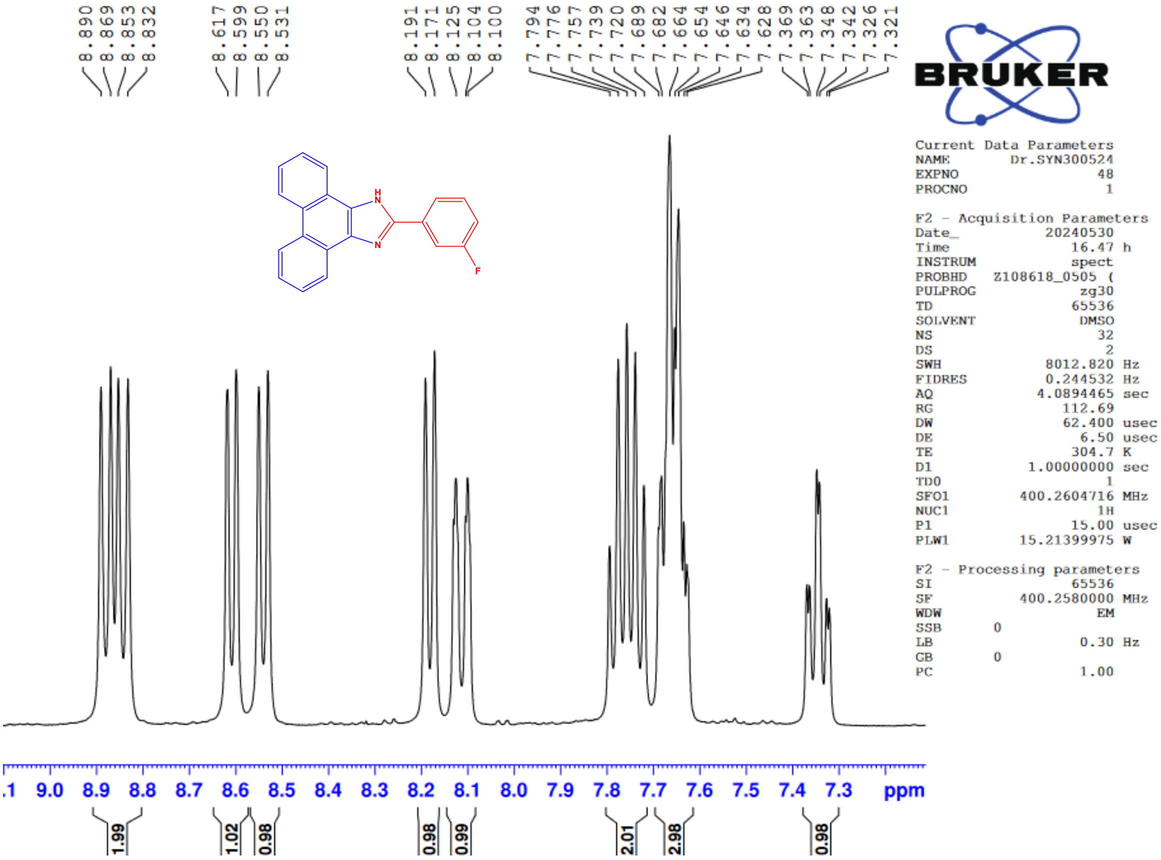
8.890
8.869
8.853
8.832
8.617
8.599
8.550
8.531
8.191
8.171
8.125
8.104
8.100
7.794
7.776
7.757
7.739
7.720
7.689
7.682
7.664
7.654
7.646
7.634
7.628
7.369
7.363
7.348
7.342
7.326
7.321
H
N
N
F
BRUKER
Current Data Parameters
NAME Dr.SYN300524
EXPNO 48
PROCNO 1
F2 - Acquisition Parameters
Date_ 20240530
Time 16.47 h
INSTRUM spect
PROBHD Z108618_0505 (
PULPROG zg30
TD 65536
SOLVENT DMSO
NS 32
DS 2
SWH 8012.820 Hz
FIDRES 0.244532 Hz
AQ 4.0894465 sec
RG 112.69
DW 62.400 usec
DE 6.50 usec
TE 304.7 K
D1 1.00000000 sec
TD0 1
SFO1 400.2604716 MHz
NUC1 1H
P1 15.00 usec
PLW1 15.21399975 W
F2 - Processing parameters
SI 65536
SF 400.2580000 MHz
WDW EM
SSB 0
LB 0.30 Hz
GB 0
PC 1.00
.1 9.0 8.9 8.8 8.7 8.6 8.5 8.4 8.3 8.2 8.1 8.0 7.9 7.8 7.7 7.6 7.5 7.4 7.3 ppm
1.99
1.02
0.98
0.98
0.99
2.01
2.98
0.98

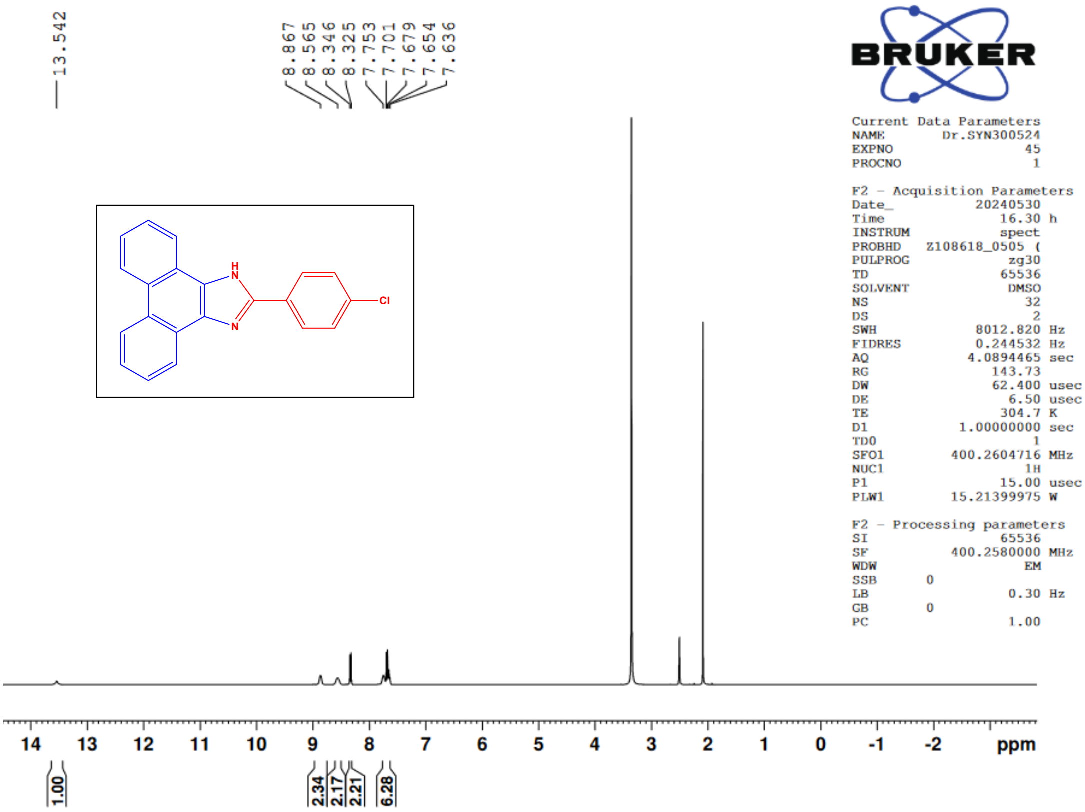
13.542
8.867
8.565
8.346
8.325
7.753
7.701
7.679
7.654
7.636
H
N
N
Cl
BRUKER
Current Data Parameters
NAME Dr.SYN300524
EXPNO 45
PROCNO 1
F2 - Acquisition Parameters
Date_ 20240530
Time 16.30 h
INSTRUM spect
PROBHD Z108618_0505 (
PULPROG zg30
TD 65536
SOLVENT DMSO
NS 32
DS 2
SWH 8012.820 Hz
FIDRES 0.244532 Hz
AQ 4.0894465 sec
RG 143.73
DW 62.400 usec
DE 6.50 usec
TE 304.7 K
D1 1.00000000 sec
TD0 1
SFO1 400.2604716 MHz
NUC1 1H
P1 15.00 usec
PLW1 15.21399975 W
F2 - Processing parameters
SI 65536
SF 400.2580000 MHz
WDW EM
SSB 0
LB 0.30 Hz
GB 0
PC 1.00
14 13 12 11 10 9 8 7 6 5 4 3 2 1 0 -1 -2 ppm
1.00
2.34
2.17
2.21
6.28

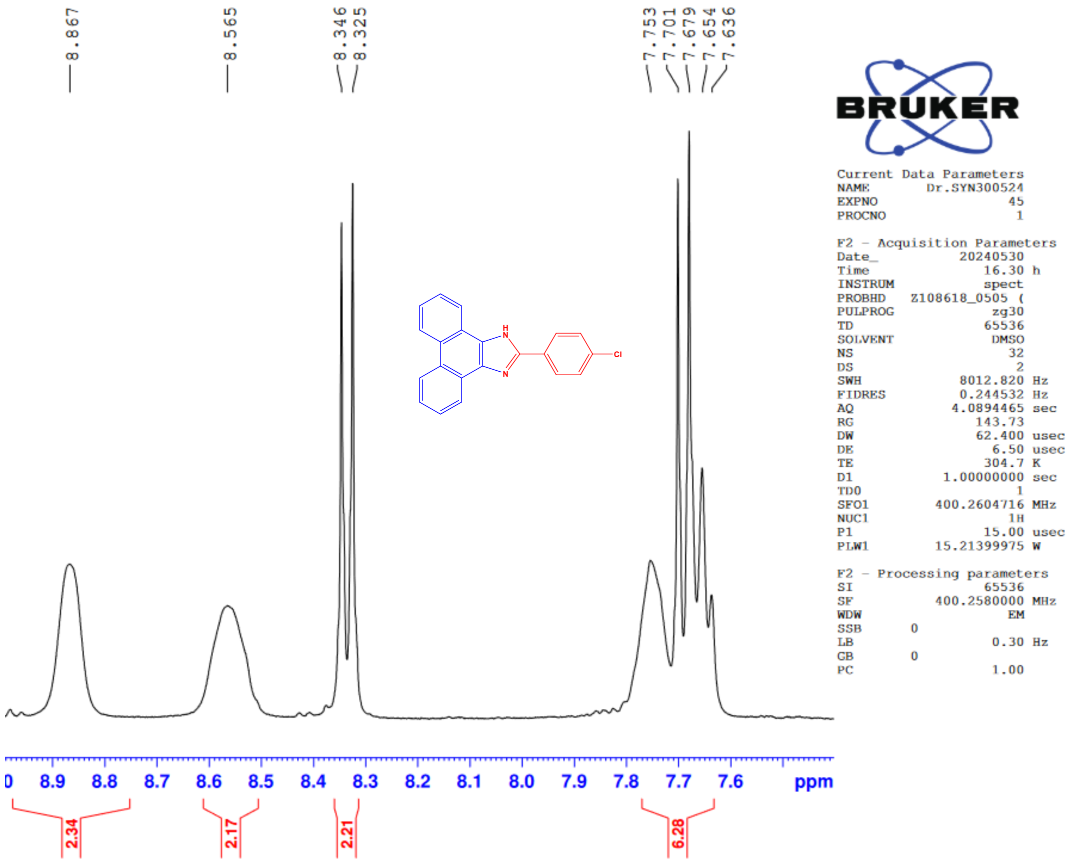
8.867
8.565
8.346
8.325
7.753
7.701
7.679
7.654
7.636
BRUKER
Current Data Parameters
NAME Dr.SYN300524
EXPNO 45
PROCNO 1
F2 - Acquisition Parameters
Date_ 20240530
Time 16.30 h
INSTRUM spect
PROBHD Z108618_0505 (
PULPROG zg30
TD 65536
SOLVENT DMSO
NS 32
DS 2
SWH 8012.820 Hz
FIDRES 0.244532 Hz
AQ 4.0894465 sec
RG 143.73
DW 62.400 usec
DE 6.50 usec
TE 304.7 K
D1 1.00000000 sec
TD0 1
SFO1 400.2604716 MHz
NUC1 1H
P1 15.00 usec
PLW1 15.21399975 W
F2 - Processing parameters
SI 65536
SF 400.2580000 MHz
WDW EM
SSB 0
LB 0.30 Hz
GB 0
PC 1.00
8.9 8.8 8.7 8.6 8.5 8.4 8.3 8.2 8.1 8.0 7.9 7.8 7.7 7.6 ppm
2.34
2.17
2.21
6.28

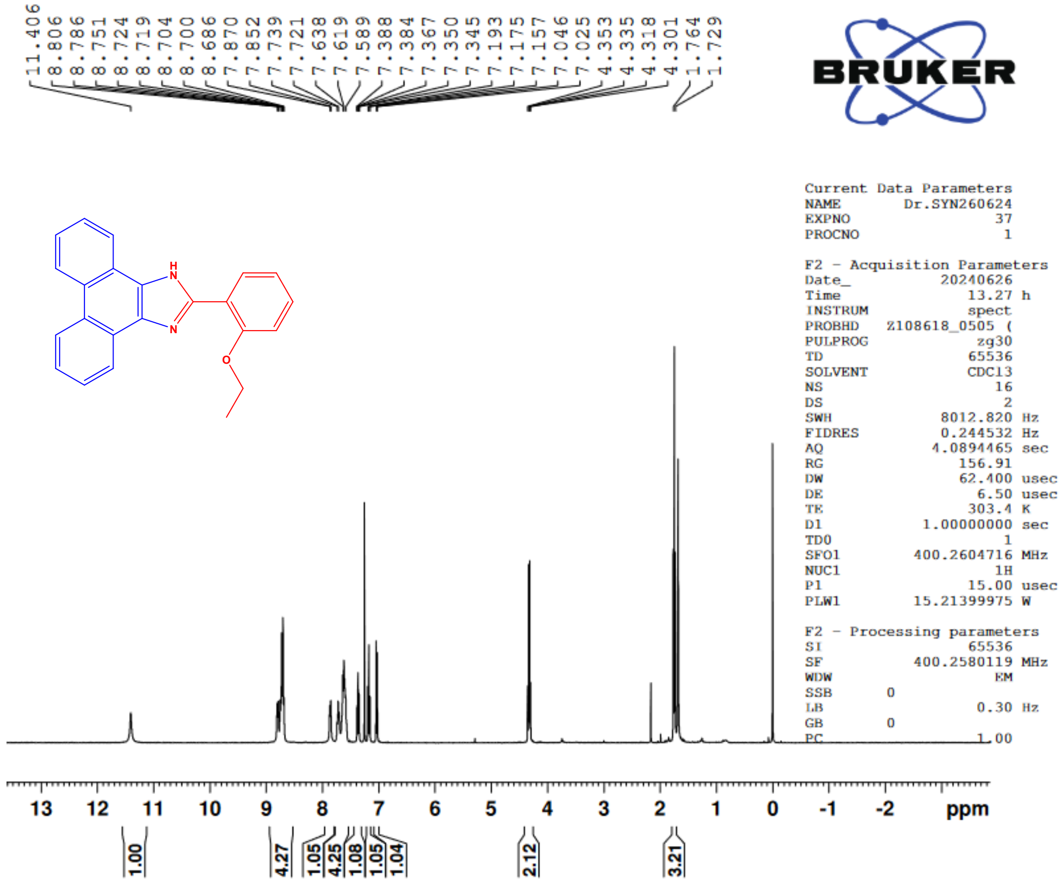
11.406
8.806
8.786
8.751
8.724
8.719
8.704
8.700
8.686
7.870
7.852
7.739
7.721
7.638
7.619
7.589
7.388
7.384
7.367
7.350
7.345
7.193
7.175
7.157
7.046
7.025
4.353
4.335
4.318
4.301
1.764
1.729
BRUKER
Current Data Parameters
NAME Dr.SYN260624
EXPNO 37
PROCNO 1
F2 - Acquisition Parameters
Date_ 20240626
Time 13.27 h
INSTRUM spect
PROBHD Z108618_0505 (
PULPROG zg30
TD 65536
SOLVENT CDCl3
NS 16
DS 2
SWH 8012.820 Hz
FIDRES 0.244532 Hz
AQ 4.0894465 sec
RG 156.91
DW 62.400 usec
DE 6.50 usec
TE 303.4 K
D1 1.00000000 sec
TD0 1
SFO1 400.2604716 MHz
NUC1 1H
P1 15.00 usec
PLW1 15.21399975 W
F2 - Processing parameters
SI 65536
SF 400.2580119 MHz
WDW EM
SSB 0
LB 0.30 Hz
GB 0
PC 1.00
13 12 11 10 9 8 7 6 5 4 3 2 1 0 -1 -2 ppm
1.00
4.27
1.05
4.25
1.08
1.05
1.04
2.12
3.21

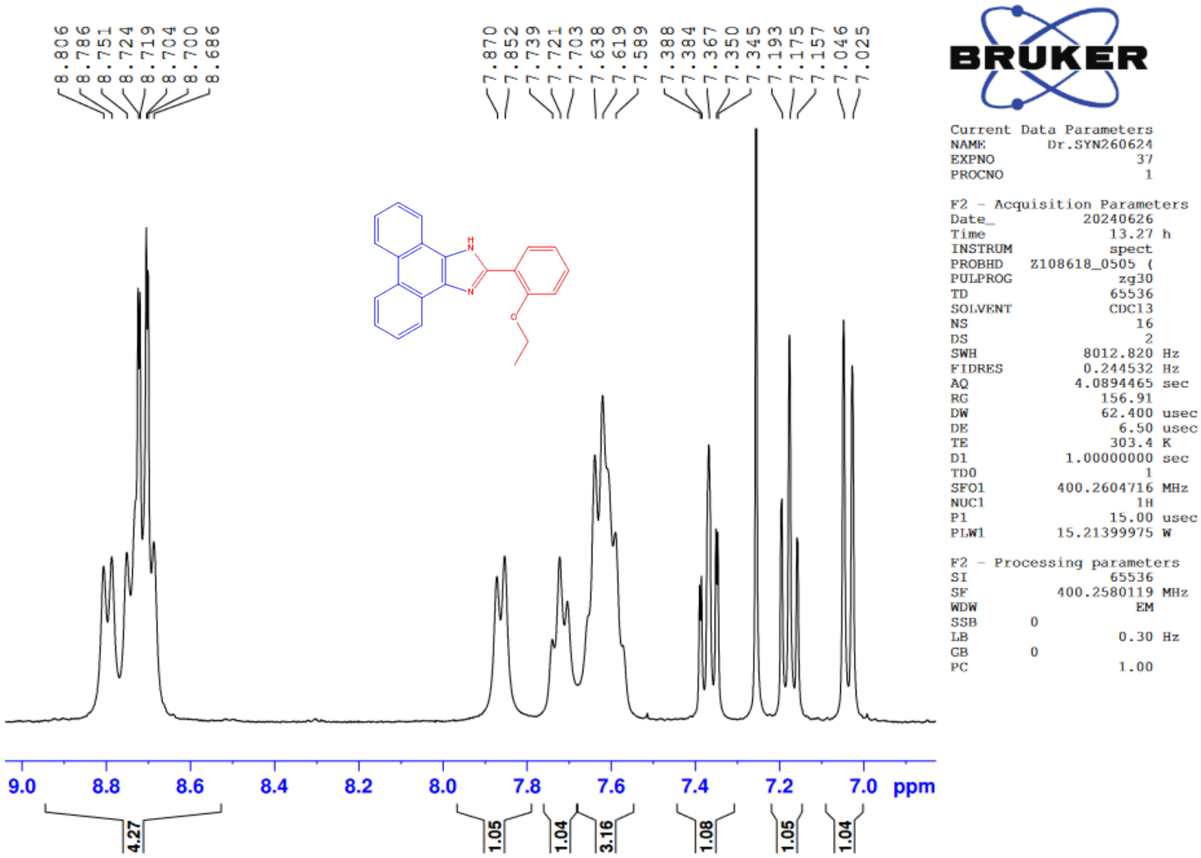
8.806
8.786
8.751
8.724
8.719
8.704
8.700
8.686
7.870
7.852
7.739
7.721
7.703
7.638
7.619
7.589
7.388
7.384
7.367
7.350
7.345
7.193
7.175
7.157
7.046
7.025
H
N
N
O
9.0 8.8 8.6 8.4 8.2 8.0 7.8 7.6 7.4 7.2 7.0 ppm
4.27
1.05
1.04
3.16
1.08
1.05
1.04
BRUKER
Current Data Parameters
NAME Dr.SYN260624
EXPNO 37
PROCNO 1
F2 - Acquisition Parameters
Date_ 20240626
Time 13.27 h
INSTRUM spect
PROBHD Z108618_0505 (
PULPROG zg30
TD 65536
SOLVENT CDCl3
NS 16
DS 2
SWH 8012.820 Hz
FIDRES 0.244532 Hz
AQ 4.0894465 sec
RG 156.91
DW 62.400 usec
DE 6.50 usec
TE 303.4 K
D1 1.00000000 sec
TD0 1
SFO1 400.2604716 MHz
NUC1 1H
P1 15.00 usec
PLW1 15.21399975 W
F2 - Processing parameters
SI 65536
SF 400.2580119 MHz
WDW EM
SSB 0
LB 0.30 Hz
GB 0
PC 1.00

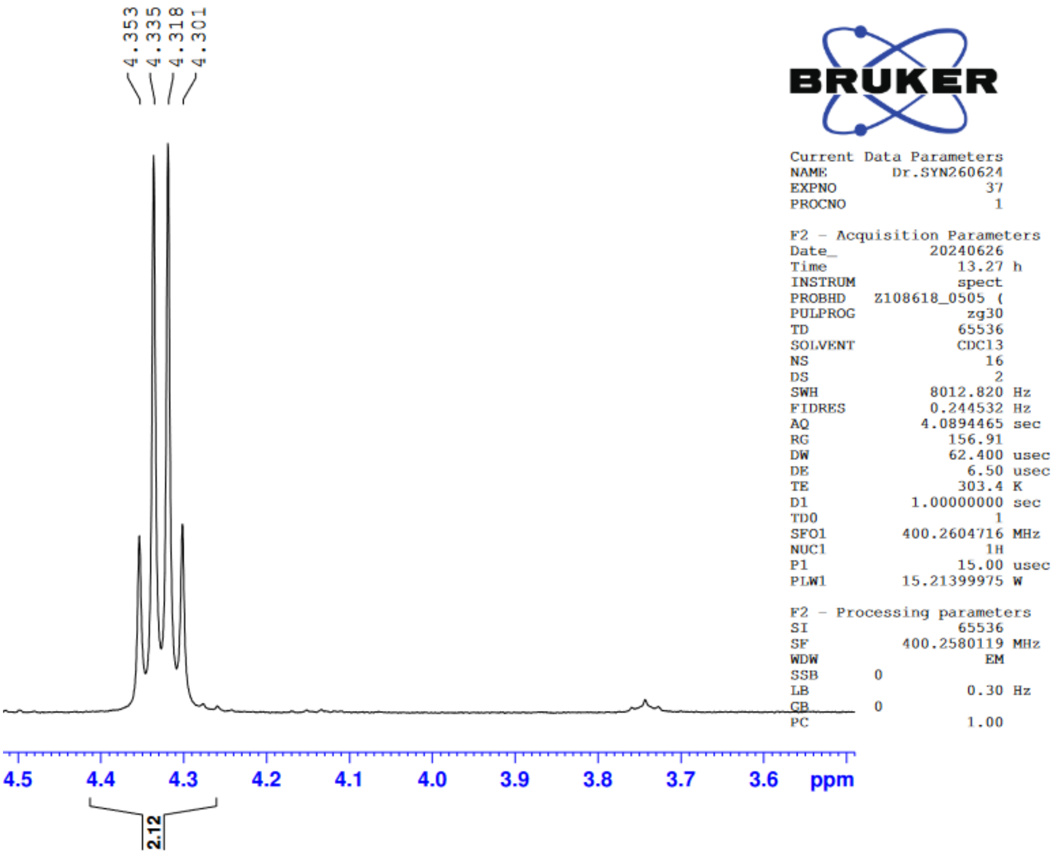
4.353
4.335
4.318
4.301
4.5 4.4 4.3 4.2 4.1 4.0 3.9 3.8 3.7 3.6 ppm
2.12
BRUKER
Current Data Parameters
NAME Dr.SYN260624
EXPNO 37
PROCNO 1
F2 - Acquisition Parameters
Date_ 20240626
Time 13.27 h
INSTRUM spect
PROBHD Z108618_0505 (
PULPROG zg30
TD 65536
SOLVENT CDCl3
NS 16
DS 2
SWH 8012.820 Hz
FIDRES 0.244532 Hz
AQ 4.0894465 sec
RG 156.91
DW 62.400 usec
DE 6.50 usec
TE 303.4 K
D1 1.00000000 sec
TD0 1
SFO1 400.2604716 MHz
NUC1 1H
P1 15.00 usec
PLW1 15.21399975 W
F2 - Processing parameters
SI 65536
SF 400.2580119 MHz
WDW EM
SSB 0
LB 0.30 Hz
GB 0
PC 1.00

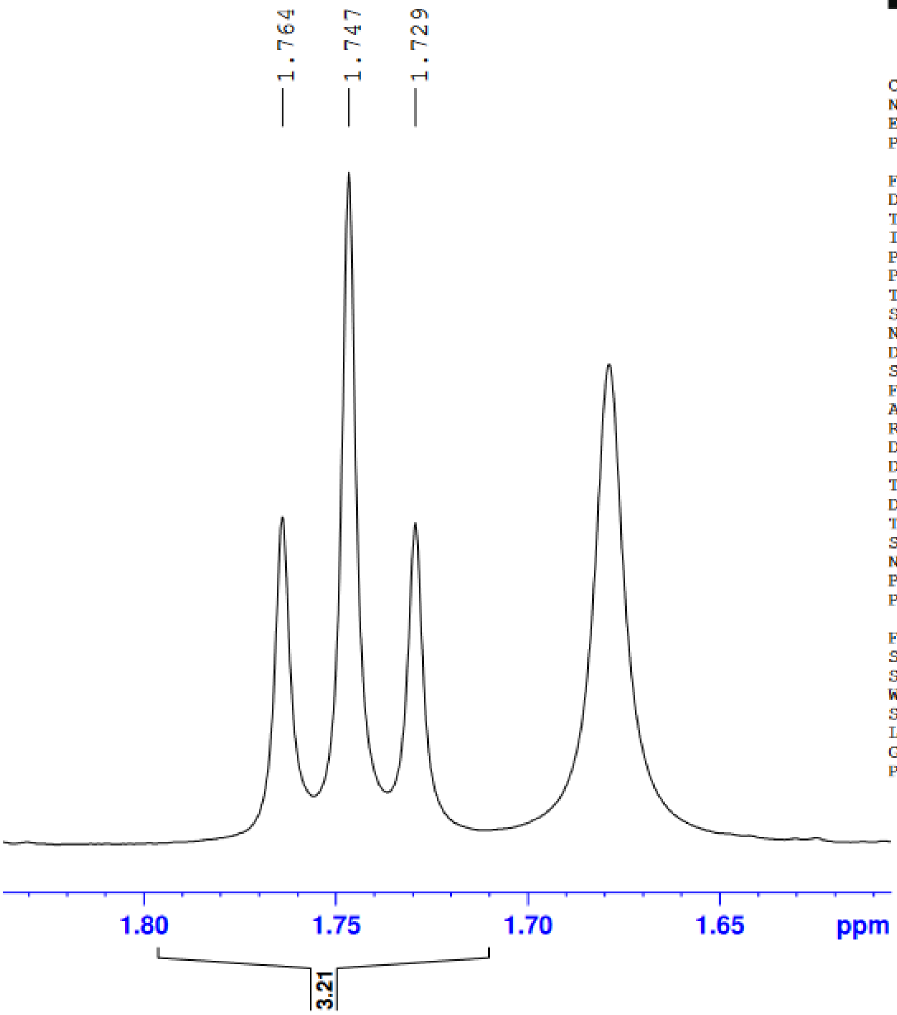

Current Data Parameters
NAME Dr.SYN260624
EXPNO 37
PROCNO 1

F2 - Acquisition Parameters
Date_ 20240626
Time 13.27 h
INSTRUM spect
PROBHD Z108618_0505 (
PULPROG zg30
TD 65536
SOLVENT CDCl3
NS 16
DS 2
SWH 8012.820 Hz
FIDRES 0.244532 Hz
AQ 4.0894465 sec
RG 156.91
DW 62.400 usec
DE 6.50 usec
TE 303.4 K
D1 1.00000000 sec
TD0 1
SFO1 400.2604716 MHz
NUC1 1H
P1 15.00 usec
PLW1 15.21399975 W

F2 - Processing parameters
SI 65536
SF 400.2580119 MHz
WDW EM
SSB 0
LB 0.30 Hz
GB 0
PC 1.00
